# On the Origin and Spread of the Scab Disease of Apple: Out of Central Asia

**DOI:** 10.1371/journal.pone.0001455

**Published:** 2008-01-16

**Authors:** Pierre Gladieux, Xiu-Guo Zhang, Damien Afoufa-Bastien, Rosa-Maria Valdebenito Sanhueza, Mohamed Sbaghi, Bruno Le Cam

**Affiliations:** 1 UMR077, INRA, Angers, France; 2 Department of Plant Pathology, Shandong Agricultural University, Taian, China; 3 National Research Centre for Grape and Wine, EMBRAPA, Bento Gonçalves, Brazil; 4 Centre Régional de la Recherche Agronomique de Kenitra, INRA, Kenitra, Morocco; Institut Pasteur, France

## Abstract

**Background:**

*Venturia inaequalis* is an ascomycete fungus responsible for apple scab, a disease that has invaded almost all apple growing regions worldwide, with the corresponding adverse effects on apple production. Monitoring and predicting the effectiveness of intervention strategies require knowledge of the origin, introduction pathways, and population biology of pathogen populations. Analysis of the variation of genetic markers using the inferential framework of population genetics offers the potential to retrieve this information.

**Methodology/Principal Findings:**

Here, we present a population genetic analysis of microsatellite variation in 1,273 strains of *V. inaequalis* representing 28 orchard samples from seven regions in five continents. Analysis of molecular variance revealed that most of the variation (88%) was distributed within localities, which is consistent with extensive historical migrations of the fungus among and within regions. Despite this shallow population structure, clustering analyses partitioned the data set into separate groups corresponding roughly to geography, indicating that each region hosts a distinct population of the fungus. Comparison of the levels of variability among populations, along with coalescent analyses of migration models and estimates of genetic distances, was consistent with a scenario in which the fungus emerged in Central Asia, where apple was domesticated, before its introduction into Europe and, more recently, into other continents with the expansion of apple growing. Across the novel range, levels of variability pointed to multiple introductions and all populations displayed signatures of significant post-introduction increases in population size. Most populations exhibited high genotypic diversity and random association of alleles across loci, indicating recombination both in native and introduced areas.

**Conclusions/Significance:**

*Venturia inaequalis* is a model of invasive phytopathogenic fungus that has now reached the ultimate stage of the invasion process with a broad geographic distribution and well-established populations displaying high genetic variability, regular sexual reproduction, and demographic expansion.

## Introduction

Biological invasions [1] by plant-pathogenic fungi are an unfortunate side effect of globalization, climate change, and more generally of the domestication of nature [2]–[4]. The Irish potato famine oomycete *Phytophthora infestans* and the chestnut blight ascomycete *Cryphonectria parasitica* are notorious examples of invasive phytopathogenic fungi that caused devastating epidemics [5], [6]. Of course, invasions do not always have tragic consequences, but invasive phytopathogenic fungi have had and continue to have diffuse and pernicious impact on agrosystems, ecosystems, and human populations dependent on them [4], [7], [8]. Because attempts to eradicate established invasive phytopathogenic fungi have met little success, the highest priorities should be given to preventing the introduction and limiting the spread and impact of established invaders [9]. The implementation of sound risk-based phytosanitary programs requires a genuinely interdisciplinary approach to seek out and utilize all available information (i) on origins, past and present introduction pathways, and population biology of invasive phytopathogenic fungi; (ii) on the interactions between social, economic, and natural processes; and (iii) on mitigation or alleviation technologies [9], [10]. In this paper, we focus on the first point, which has important applications for monitoring and predicting the effectiveness of intervention strategies [11]. The origin and introduction routes of many invasive phytopathogenic fungi are unknown, even for those causing major economic and ecological impact. One reason is that many introductions occurred when very little attention was paid to risks associated with the disease, as neither the nature of the cause of diseases nor the way in which they spread were understood [7], [8]. Some invasive phytopathogenic fungi spread so long ago that it probably does not come to mind that they are invasive [7]; others have so broad distributions that they are listed as cosmopolitan, though they were initially restricted to a specific area [12].

Although some invasive phytopathogenic fungi can naturally move over broad geographic areas (e.g., *Claviceps africana*, [13]) or even overseas (e.g., *Aspergillus sydowii* [14] or *Hemileia vastatrix* [15]), most long-distance movements are assisted by human activities [12]. Introductions can be deliberate as in the case of biocontrol agents or the unintended consequence of decisions involving the use of nonindigenous species in agriculture and forestry, alteration of habitat, or movements of goods and people [10], [12], [16]. The domestication and spread of agricultural food crops provided opportunities for invasions by phytopathogenic fungi. The spread of agriculture and the globalization of travel and trade were associated with extensive movements of crop species and plant products that allowed accidental transportations of fungal pathogens far from their native range [17].

In the absence of detailed information on the origin, introduction pathways and population biology of invasive phytopathogenic fungi, analysis of the variation of molecular markers in the framework of population genetics theory can serve as a powerful alternative. In the vocabulary of population genetics, bioinvasions are rapid range expansions involving four steps: movement, arrival, establishment, and spread [12], [18]. The rationale behind population genetics inference is that each of these steps leaves an imprint in the distribution of genetic variation within and among populations (i.e., the population structure) that can help distinguish among possible competing hypotheses on the history of the bioinvasion process and the population biology of the invasive species.

The first hurdle for invasive phytopathogenic fungi is to arrive in the new range by exploiting introduction pathways [16]. Various source populations can contribute to the genetic makeup of introduced populations, and several methods exist that allow determining historical source and sink patterns of migration among populations. For instance, Fisher et al. [19] and Fisher et al. [20] used measures of genetic similarity among genotypes and assignment methods to infer the source populations for isolates of the human pathogen *Coccidioides immitis* collected outside the endemic area of the fungus. For the wheat pathogens *Mycosphaerella graminicola* and *Phaeosphaeria nodorum*, Banke and Mc Donald [21] and Stukenbrock et al. [22] used coalescent-based estimates of gene flow to analyze historical patterns of global migration into or among new territories.

Introduction events may involve a population bottleneck because the number of initial colonists is often small [18], [23]. Loss of alleles and reduction in genetic variation can also occur during the early stages of establishment because of random genetic drift due to small population size and selective pressure exerted by novel environments [16], [24]. Thus, a newly established population is likely to be much less variable than the older population(s) from which it is derived, and populations from the centre of origin of the invasive phytopathogenic fungi are expected to be the most variable [25]. *Ceratocystis fimbriata* and *Phytophthora ramorum*, causal agents of canker stain of plane tree and sudden oak death, are examples of invasive phytopathogenic fungi that have very limited variation in their area of introduction [26], [27]. For other invasive phytopathogenic fungi such as *Phaeosphaeria nodorum* or *Sphaeropsis sapinea*, responsible for leaf and glume blotch of wheat and pine pitch canker, population genetics studies found more substantial levels of variation, pointing toward multiple introduction events [8], [22]. Genetic bottlenecks may also transitorily throw populations out of mutation-drift equilibrium [28]. Rivas et al. [29] used this approach to show that African, Latin American, and Caribbean populations of the causal agent of black leaf streak disease of bananas (*M. fijiensis*) had been recently founded.

Moving a fungus from its native biogeographic range to a novel environment can also change its population structure and reproductive mode [30]. Random association among alleles from different loci is a reasonable null hypothesis for fungi known to have a sexual stage [31]. However, even for a normally recombining population, factors such as foundation by a limited number of individuals, random genetic drift in small populations, or immigration of individuals from populations with different allele frequencies can artificially create nonrandom associations between unlinked markers (i.e., linkage disequilibrium) at the time of colonization of a new territory. This spurious linkage disequilibrium should quickly dissipate with periodic recombination and population growth [32], provided the introduced population has not lost sexual competence. Indeed, chance effects such as establishment of only one mating type, mating-type linkage to avirulence genes, or introduction into an environment not being conducive to meiospore development can result in the establishment of nonsexual populations [33]. A well-documented illustration of this phenomenon is the movement of *P. infestans* outside Mexico: the founding of European and North American populations by a single genotype of the A1 mating type rendered reproduction exclusively mitotic for 120 years [34].

Following movement, arrival, and establishment of a viable population, the final step in a biological invasion is the spread to additional locations within the new territory [12]. The dispersal mode can range between rare, unpredictable long-distance founder events to a gradual expansion [29]. Depending on factors such as dispersal abilities and reproduction mode of the invasive phytopathogenic fungus, or density and susceptibility of hosts, populations can experience a rapid expansion, producing an increase in effective population size and deviation from mutation-drift equilibrium [35]. Fisher et al. [19] used this feature to show that South American populations of the human pathogen *Coccidiodes immitis* had undergone rapid population growth, indicating an epidemic increase in postcolonization population size.

The fungal pathogen *Venturia inaequalis* is the agent of the scab disease of apple, the most important disease in apple production. *Venturia inaequalis* is a heterothallic haploid ascomycete that reproduces both sexually and asexually [36]. During winter, the fungus grows as a saprobe in dead apple leaves and produces meiospores (ascospores). In spring, when temperature and moisture conditions are favorable, ascospores are released and dispersed by wind to initiate epidemics. When an ascospore lands on a susceptible fruit or leaf, it germinates and proceeds to form lesions producing mitospores (conidia) that are blown by wind or splashed by rain to cause secondary infections. Both ascospores and conidia have limited dispersal capacities: conidia are only dispersed over a few meters, ascospores do not spread over a hundred meters, and wind distribution of infected leaves probably does not exceed a few kilometers. The only way to achieve long-distance dispersal is man-mediated transportation of infected fruits or plants. Based on this feature, the population structure of the pathogen is expected to mirror historical movements of its host [37].

The history of apple is well documented. It is now widely accepted that the centre of origin of apple (*Malus*×*domestica*) is in the mountain ranges of Central Asia [38], [39]. As early as Neolithic times (5,000–8,000 years before present), this region was crossed by the famous Silk Roads stretching from Rome in Italy through Samarkand in Uzbekistan to Luoyang in China [40]. Travelers, ably assisted by their domesticated animals, progressively began to domesticate and transport apples westward. Apple cultivation likely began in the region between the Caspian and Black seas, and it had reached the Near East by 3,000 years before present [41]. The Romans introduced and spread apple across the European and Mediterranean areas and European settlers transported it into newfound lands during the last 500 years. Apple is now grown in all temperate regions [42].

Today, *V. inaequalis* has invaded all apple-growing regions. The disease has a negative economic impact due to yield losses, the cost of breeding programs aimed at producing resistant varieties and the use of fungicide inputs, with the corresponding environmental and health hazards. Despite this detrimental effect, the population biology of the fungus outside Europe is virtually unknown [43]–[45] and, unlike apple, its origin and introduction pathways are not documented. The present study was conducted to make up for this lack of knowledge. We used multilocus microsatellite typing [46] to describe the population structure of a set of samples from Central Asia, Europe, North Africa, and newfound lands (North and South America, Australasia, South Africa). Our analyses revealed that *V. inaequalis* emerged in Central Asia and followed its host into Europe along the Silk Roads and more recently into newfound lands with the expansion of apple growing. *Venturia inaequalis* appeared as a model of invasive phytopathogenic fungus that has reached the ultimate stage of the invasion process with a broad geographic distribution and well-established populations displaying high genetic variability, regular sexual reproduction, and demographic expansion [*Nota bene*: because this study is the fruit of an international collaborative effort, abstracts in Chinese, Portuguese, French and Moroccan are available as Supplementary Information (Text S1)].

## Materials and Methods

### Sample collection

We collected 1,273 individual fungal strains of *V. inaequalis* from *M.*×*domestica* on 28 locations representing seven regions in five continents: Central Asia (Xinjiang Province of China, Iran, Azerbaijan), Europe (France, Sweden, Spain), North Africa (Morocco), South Africa, North America (Canada, USA), South America (Brazil), and Australasia (New Zealand) (Table 1, Figure S1). All samples represented single orchards, except the sample from Canada that originated from several locations and various host cultivars. In each orchard, infected leaves were sampled randomly and we collected only one leaf per apple tree.

**Table 1:** Origin of *Venturia inaequalis* samples.

Sample ID	Country	State/Province	Location	GPS Position	Host Cultivar	Year of collection	Provider/Collector
CN_1_	China	Xinjiang	Kazakhstan Border	44°15N, 80°39E	Golden Delicious	2005	PG, BLC & XGZh
CN_2_	China	Xinjiang	Kazakhstan Border	44°15N, 80°39E	Fuji	2005	PG, BLC & XGZh
CN_3_	China	Xinjiang	Kazakhstan Border	44°15N, 80°39E	Royal Gala	2005	PG, BLC & XGZh
CN_4_	China	Xinjiang	Xinyuan	43°25N, 83°32E	…	2005	PG, BLC & XGZh
CN_5_	China	Xinjiang	Yili suburbs	43°56N, 81°18E*	Golden Delicious	2005	PG, BLC & XGZh
CN_6_	China	Xinjiang	Yili suburbs	43°56N, 81°18E*	New Century	2005	PG, BLC & XGZh
CN_7_	China	Xinjiang	Yili suburbs	43°56N, 81°18E*	Royal Gala	2005	PG, BLC & XGZh
IR_1_	Iran	Khorasan	Bojnurd	37°54N, 55°57E	Golden Delicious	2004	Farhad Shokoohifar & Mehdi Alavi
IR_2_	Iran	Khorasan	Esfarayen	37°04N, 58°00E	Golden Delicious	2004	Farhad Shokoohifar & Mehdi Alavi
IR_3_	Iran	Khorasan	…	…	Golden Delicious	2004	Farhad Shokoohifar & Mehdi Alavi
AZ	Azerbaijan	Quba	Quba	40°22N, 49°53E*	Starkrimson	2005	Fabien Martel
F_1_	France	Basse-Normandie	Le Sacq	48°54N, 01°04E	Bisquet	2005	BLC & PG
F_2_	France	Rhônes-Alpes	Loriol	44°40N, 04°49E	Fuji	2005	Christelle Gomez
F_3_	France	Rhônes-Alpes	Saint Marcel les Valence	45°00N, 05°00E	Mutsu	2005	Laurent Brun
F_4_	France	Pays de la Loire	Aviré	47°42N, 00°47W	Royal Gala	2005	BLC
F_5_	France	Pays de la Loire	Aviré	47°42N, 00°47W	Jonagold	2005	BLC
F_6_	France	Rhônes-Alpes	Saint Marcel les Valence	45°00N, 05°00E	Smoothee	2005	Laurent Brun
F_7_	France	Nord Pas de Calais	Villeneuve d'Ascq	50°41N, 03°08E	Royal Gala	2005	BLC
F_8_	France	Nord Pas de Calais	Villeneuve d'Ascq	50°41N, 03°08E	Golden Delicious	2005	BLC
SE	Sweden	Öresund	Alnarp	55°40N, 13°05W	Royal Gala	2005	Hilde Nybom
SP_1_	Spain	Catalonia	Girona	42°07N, 02°43E	Royal Gala	2005	Marta Pujol
SP_2_	Spain	Catalonia	Girona	42°07N, 02°43E	Golden Delicious	2005	Marta Pujol
SP_3_	Spain	Catalonia	Girona	42°07N, 02°43E	Wellspur	2005	Marta Pujol
MA_1_	Morocco	Gharb	Moghrane	34°25N, 06°26W	Chimère	2005	PG, BLC & MS
MA_2_	Morocco	Gharb	Moghrane	34°25N, 06°26W	Anna	2005	PG, BLC & MS
MA_3_	Morocco	Gharb	Mechraâ Bel Ksiri	34°34N, 06°00W	Anna	2005	PG, BLC & MS
MA_4_	Morocco	Gharb	Mechraâ Bel Ksiri	34°34N, 06°00W	Dorsett	2006	PG, BLC & MS
MA_5_	Morocco	Gharb	Mechraâ Bel Ksiri	34°34N, 06°00W	Royal Gala	2006	PG, BLC & MS
US_1_	U.S.A.	Illinois	Geneva	41°00N, 88°00W	Cortland	2006	Mickael Malnoy
US_2_	U.S.A.	Illinois	Geneva	41°00N, 88°00W	Mac Intosh	2006	Mickael Malnoy
US_3_	U.S.A.	Indiana	Lafayette	40°25N, 86°53W	Red Delicious	2006	John Hartman
US_4_	U.S.A.	Kentucky	Versailles	38°03N, 84°44W	Royal Gala	2006	John Hartman
CA	Canada	Québec	Various	45°19N, 73°19W*	Various	Various	Odile Carisse
BR_1_	Brazil	Rio do sul	São João	29°20S, 50°24W	Golden Delicious	2004	BLC & RMVS
BR_2_	Brazil	Rio do sul	Cambará	29°14S, 50°16W	Belgolden	2004	BLC & RMVS
BR_3_	Brazil	Rio do sul	Ana Rech	29°05S, 51°00W	Royal Gala	2004	BLC & RMVS
BR_4_	Brazil	Rio do sul	Ana Rech	29°05S, 51°00W	Fuji	2004	BLC & RMVS
BR_5_	Brazil	Rio do sul	Vacaria	28°30S, 50°49W	Royal Gala	2004	BLC & RMVS
BR_6_	Brazil	Rio do sul	Fischer Company	27°04S, 50°56W	Royal Gala & Fuji	2004	BLC & RMVS
BR_7_	Brazil	Rio do sul	EPAGRI Institute	27°04S, 50°56W	Royal Gala	2004	BLC & RMVS
SA	South Africa	Western Cape	Grabow	33°58S, 18°30E*	Golden Delicious	2005	Iwan Labuschagné
NZ_1_	New Zealand	Hawke's Bay	…	39°40S, 176°53E*	Royal Gala	2005	Kim Plummer
NZ_2_	New Zealand	Hawke's Bay	…	39°40S, 176°53E*	Fuji	2006	Vincent Bus

* for the samples from Azerbaijan, Canada, New Zealand and South Africa the geographic positions reported are those of, respectively, Baku, Saint Jean sur Richelieu, Havelock North and Cape Town. For the samples CN_5_, CN_6_ and CN_7_ the position reported is the one of Yili.

Host resistance can induce selective and/or demographic sweeps in fungal populations, leading to lineages highly divergent from populations found on susceptible cultivars [47]–[49]. To avoid confounding geographic structure with possible associations between host cultivars and fungal genotypes (i) we sampled on cultivars with no known effective resistance; (ii) we minimized the total number of cultivars sampled by focusing as much as possible on the commercially leading cultivars Fuji, Royal Gala, and Golden Delicious; and (iii) we collected samples on different cultivars at several locations and checked for the absence of associations between host and *V. inaequalis* genotypes by calculating pairwise *φ_ST_* between samples (an analog of Wright's *F_ST_* fixation index) [50] using Genalex [51]. As pairwise *φ_ST_* values were low or nonsignificantly different from zero (Table S2), samples from the same location were pooled for all subsequent analyses, except the samples from Mechraâ Bel Ksiri in Morocco. We obtained a total of 29 samples.

### Microsatellite Multilocus Typing

DNA was extracted from monoconidial isolates or directly from infected leaf symptoms according to a protocol described in previous studies [48], [49]. Samples were genotyped at 12 microsatellite loci: *1tc1a, 1tc1b, 1tc1g, 1aac3b* [45], *Vitc1/2, Vitcca7/P, Vitg11/70, Vicacg8/42, Vica9/152, Viga7/116, Vica9/X* [52], and *M42* [49]. Polymerase chain reaction was performed with the fluorescently labeled primers and conditions described previously [52]. Alleles were scored against a fluorescently labeled size standard in an ABI 310 automated sequencer (Applied Biosystems, Foster City, California). Our data set is accessible via the Internet at http://www.multilocus.net/ (Table S1).

### Data analysis

#### Genetic variation within samples

The number of haplotypes was calculated using Arlequin 3.00 [53], and it was used to quantify the clonal fraction [54]. We treated multilocus haplotypes repeated multiple times as clones. For all subsequent analyses, we used a data set in which each multilocus haplotype was represented only once in each sample [55].

Expected heterozygosity [56], allelic richness, and unique allele richness were computed using scripts written in Matlab (The Mathworks, Natick, Massachusetts). Unique allele richness represents the number of alleles that are unique to a particular sample in comparisons with all other samples, averaged across loci. To account for differences in sample size, samples were standardized to a uniform size equal to the size of the smallest sample (South Africa: 12 individuals) using random draws with replacement (nonparametric bootstrapping) [57], [58]. For each sample, expected heterozygosity, allelic richness, and unique allele richness indices were calculated as the average value of 100 bootstrap replicates [54]. We examined correlations between these variability indices and geographical distance calculated as the arc surface distance from the most eastern Chinese sample. Because the variables tested may not be distributed normally, all correlations were nonparametrically tested using Spearman *r* available in Graphpad (GraphPad Software Inc., San Diego, California).

Associations of alleles among different loci were examined in each sample using the index of association (*I_A_*) statistic, which is a generalized measure of multilocus linkage disequilibrium [59]. The null hypothesis of random association of alleles (*I_A_* = 0), consistent with random mating, was tested using the program Multilocus [60] by comparing the observed value of the statistic to that obtained after 1,000 randomizations to simulate recombination.

#### Genetic variation among samples

We compared levels of genetic variation among regional groups of samples. To account for differences in group sizes, we used nonparametric bootstrapping to standardize group sizes to the size of the smallest group (South Africa: 12 individuals) using a script written in Matlab [54], [61]. Expected heterozygosity, allelic richness, and unique allele richness were calculated as the average value of 100 bootstrap replicates. The level of genetic variation among groups was compared in SPSS 10 (SPSS Inc., Chicago, Illinois) using a one-way ANOVA followed by a post-hoc Tukey test.

One-way and two-way hierarchical analyses of molecular variance (AMOVA) were used to partition microsatellite variation among regions, among samples, and within samples [50]. Only regions with more than two samples were included in analyses. Genalex [51] was used to compute and test the statistical significance of *φ-*statistics based on 1,000 permutations.

#### Demographic history

We used three methods designed to detect historical changes in population size from deviations from mutation-drift equilibrium. The first method, implemented in the program Bottleneck [62], compares the expected heterozygosity estimated from allele frequencies with that estimated from the number of alleles and the sample size, which are expected to be identical in a neutral locus in a population at mutation-drift equilibrium. Inferences about historical demographics are based on the prediction that populations that have experienced a recent reduction of their effective size see their expected heterozygosity estimated from allele frequencies reduce faster than that estimated under a given mutation model at mutation-drift equilibrium; the contrary is expected for growing populations [28]. The tests were performed under the stepwise-mutation model (SMM) as well as under a two-phase model (TPM), allowing for 30% of multistep changes. Significance was tested using two-sided Wilcoxon signed rank tests.

The second method relies on the notion that variance- and homozygosity-based estimates of the population mutation rate *θ* are expected to be equal in a neutral locus in a population at mutation-drift equilibrium [63], [64]. The deviation between the two estimators, measured by the imbalance index *β* (equation 7 in reference [64]), can be used to detect population expansion: Ln *β* is expected to be negative for populations that have recently expanded from equilibrium initial conditions and positive for populations that have recently expanded following a bottleneck. 95% confidence intervals estimated by bootstrapping over loci were computed using a script written in Matlab [35].

The third method uses the principle that the variance of the variance in allele lengths is expected to be larger in a constant-sized than in a growing population, assuming that the loci follow an SMM. This difference was quantified using the interlocus *g* statistic [65] and significance was tested using the fifth-percentile cutoffs of Reich et al. [66]. Since the *β* and *g* statistics assume that loci evolve under the SMM, loci *Vica9/152* and *Vicacg8/42* were excluded from the calculations.

#### Clustering and assignment methods

We used four different methods to determine the optimal number of populations present in our data set, to assess the level of differentiation, to infer the geographic ancestral relationships among these populations, and to identify recently founded populations, as these are expected to cluster with their source population.

First, we calculated principal coordinates on Cavalli-Sforza and Edwards' chord distance among samples [67]. The chord distance matrix was built using the Microsatellite analyzer (MSA 4.00) software [68], and principal component analysis was performed under Genalex.

Second, we used the Bayesian clustering algorithm implemented in Structure 2.1 [69], [70]. This method relies on the Bayesian Monte Carlo Markov Chain (MCMC) approach to cluster individuals into *K* distinct populations that minimize Hardy-Weinberg disequilibrium and gametic phase disequilibrium between loci within groups. The model allowed individuals to have mixed ancestry and correlation of allele frequencies. Uniform priors were assumed and the MCMC scheme was run for 500,000 iterations after an initial burn-in period of 50,000. We ran Structure for *K* ranging from 1 to 13 and we performed at least six repetitions to check for convergence of likelihood values for each value of *K*. Convergence of the MCMC could not be achieved for *K* values higher than 13. The number of populations that best represents the observed data under the model implemented was determined by maximizing the estimated Ln likelihood of the data for different values of *K*.

Third, we used the Bayesian clustering algorithm implemented in Baps 4 [71] to identify the optimal number *K* of partitions among groups of samples. By contrast to the individual-based algorithm applied in Structure, we used the group-level option in Baps such that clusters are formed by assembling whole samples. Baps 4 relies on stochastic optimization to infer the posterior mode of the genetic structure. The program was run for *K* ranging from 1 to 29 with five replicates for each value of *K* to ensure that the stochastic optimization algorithm had not ended up in different solutions in separate runs. Goodness-of-fit levels of the clustering solutions to the data set are compared in terms of natural logarithm of the marginal likelihood of the data. We also used Baps to perform an admixture analysis aiming at estimating individual coefficients of ancestry with regard to the inferred clusters of samples. For this analysis, we used 1,000 iterations to estimate the admixture coefficients for the individuals, we used 200 reference individuals from each cluster, and we repeated the admixture analysis 50 times per individual.

Fourth, we used Geneclass 2.0 [72] to assign individuals to regional groups of samples. The probability of individuals coming from each area was calculated using the standard criterion described by Rannala and Mountain [73] and by simulating 1,000 individuals per regional group of samples using the method of Paetkau et al. [74]. Individuals were assigned to a regional group when this group had the highest probability of being the source of this individual.

#### Gene flow and effective population size

We used the program Migrate 2.0 [75] to assess long-term gene flow and effective population sizes and to determine which migration route was most supported by the data. These analyses were performed on regional groups of samples.

Migrate uses an expansion of the coalescent theory to estimate migration rates between populations (*N_e_m*) and *θ* (2*N_e_μ*), where *N_e_* is the effective population size, *m* is the constant migration rate between population pairs, and *μ* is the mutation rate per generation at the locus considered. Likelihood surfaces for each parameter were estimated by simulating genealogies using an MCMC approach. The computations were carried out under a Brownian motion approximation of the SMM, with the loci *Vica9/152* and *Vicacg8/42* excluded from the data set. We evaluated two migration models: a full migration model with unrestricted migration among all groups (Model 1) and a migration model with the Central Asian group exchanging migrants only with European group and unrestricted migration among all non-Central Asian groups (Model 2). The models were run three times to confirm convergence of parameter estimates, and only the results of the run that yielded the highest Ln likelihood value are presented. The runs consisted of two replicates of 10 short chains (with 10,000 genealogies sampled) and three long chains (with 100,000 genealogies sampled), with the first 10,000 genealogies discarded. A likelihood ratio test was used to compare the likelihoods of all models [75].

## Results

### Polymorphism and multilocus linkage disequilibrium

Among the 1,273 individuals analyzed, we found 1,180 unique haplotypes based on 12 microsatellite loci, representing a total of 221 different alleles. The number of alleles at each locus ranged from 6 at *1aac3b* to 32 at *1tc1g*, with an average value of 18.4 (±7.1 SD).

**Figure 1:**
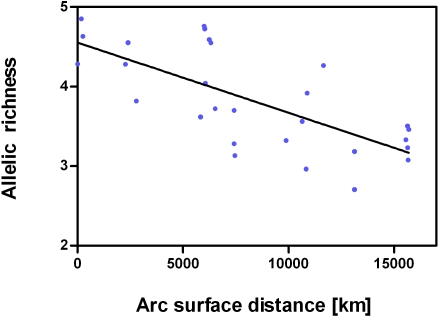
Scatterplot of allelic richness and arc surface distance from the most eastern Chinese sample. A least-square regression line represents the relationship between the two variables. Significance of the correlation was tested using Spearman's *r* (*r* = −0.74, *P*<0.0001).

Estimates of variation indices for each sample are reported in Table 2. Allelic richness ranged from 2.71 to 4.85 (mean±SD: 3.84±0.62), expected heterozygosity ranged from 0.43 to 0.65 (mean±SD: 0.56±0.06), and unique allele richness ranged from 0.01 to 0.29 (mean±SD: 0.12±0.09). All three variation indices were negatively correlated with arc surface distance from the most eastern Chinese sample (*A*: *r* = −0.74, *H_E_*: *r* = −0.66, *P*<0.0001; *n_ua_*: *r* = −0.58, *P* = 0.0013) (Figure 1; Figure S2).

**Table 2:** Polymorphism summary and test for multilocus linkage disequilibrium in *Venturia inaequalis* samples.

Samples	*n* a	*K* b	*CF* c	*A* d,g	*H_E_* e,g	*n_ua_* f,g	*I_A_* h	*P* h
**Central Asia**								
CN_1,2,3_	103	99	0.04	4.63±0.41	0.63±0.03	0.25±0.13	−0.05	0.796
CN_4_	40	39	0.03	4.29±0.38	0.59±0.04	0.13±0.11	0.04	0.366
CN_5,6,7_	120	115	0.04	4.85±0.36	0.64±0.03	0.29±0.14	0.12	0.025
IR_1_	47	47	0.00	4.56±0.31	0.62±0.03	0.18±0.13	0.03	0.385
IR_2_	31	29	0.06	4.28±0.35	0.59±0.03	0.08±0.08	0.15	0.143
IR_3_	18	18	0.00	4.13±0.40	0.59±0.04	0.24±0.11	0.08	0.322
AZ	19	19	0.00	3.82±0.40	0.58±0.04	0.19±0.09	0.19	0.141
**Europe**								
F_1_	33	29	0.12	4.04±0.34	0.60±0.04	0.06±0.06	0.25	0.04
F_2_	29	29	0.00	4.73±0.44	0.65±0.03	0.28±0.13	0.03	0.382
F_3,6_	48	48	0.00	4.76±0.40	0.65±0.03	0.26±0.13	0.05	0.254
F_4,5_	47	47	0.00	4.59±0.29	0.63±0.03	0.14±0.09	0.14	0.052
F_7,8_	42	42	0.00	3.62±0.28	0.55±0.03	0.04±0.05	0.14	0.033
SE	26	25	0.04	3.72±0.29	0.59±0.03	0.14±0.09	0.10	0.212
SP_1,2,3_	82	75	0.09	4.55±0.30	0.63±0.02	0.10±0.09	0.03	0.276
**Morocco**								
MA_1,2_	55	32	0.42	3.13±0.36	0.47±0.05	0.03±0.05	0.97	<0.001
MA_3,4_	61	51	0.16	3.28±0.27	0.50±0.04	0.03±0.05	0.19	0.027
MA_5_	25	23	0.08	3.70±0.28	0.60±0.03	0.08±0.07	0.20	0.074
**North America**								
US_1,2_	45	45	0.00	4.27±0.28	0.60±0.03	0.08±0.08	−0.02	0.582
US_3_	29	29	0.00	3.56±0.28	0.53±0.03	0.08±0.08	−0.05	0.657
US_4_	33	33	0.00	3.92±0.33	0.55±0.03	0.16±0.09	0.12	0.162
CA	34	29	0.15	3.32±0.32	0.51±0.03	0.11±0.08	0.28	0.024
**Brazil**								
BR_1_	28	27	0.04	3.08±0.26	0.46±0.04	0.07±0.07	0.25	0.053
BR_2_	39	39	0.00	3.23±0.23	0.52±0.03	0.12±0.08	−0.10	0.875
BR_3,4_	58	57	0.02	3.46±0.24	0.53±0.03	0.04±0.05	0.01	0.448
BR_5_	58	42	0.28	3.51±0.25	0.55±0.03	0.01±0.03	−0.06	0.789
BR_6,7_	45	44	0.02	3.33±0.26	0.51±0.03	0.01±0.02	0.02	0.392
**South Africa**								
SA	12	12	0.00	2.97±0.31	0.47±0.06	0.20±0.10	0.67	0.010
**New Zealand**								
NZ_1_	27	24	0.11	2.71±0.25	0.43±0.04	0.01±0.03	0.28	0.027
NZ_2_	39	39	0.00	3.19±0.23	0.49±0.04	0.07±0.06	−0.08	0.797

a sample size.

b number of haplotypes.

c clonal fraction.

d allelic richness.

e expected heterozygosity [56].

f unique allele richness.

g for each sample, values (±standard deviation) are averaged across loci and across 100 resampled datasets of 12 individuals.

h index of association [59]. The null hypothesis of random association of alleles (*I_A_* = 0), consistent with random mating, was tested using the program Multilocus [60] by comparing the observed value of the statistic to that obtained after 1000 randomization to simulate recombination.

Bootstrap analysis revealed significant differences in *A*, *H_E_* and *n_ua_* among regional groups of samples (ANOVA, *P*<0.001). Central Asian *V. inaequalis* showed significantly higher values for all three measures of variation (*A* = 5.03, *H_E_* = 0.65, *n_ua_* = 1.12, *P*<0.001) than all other regional groups of samples (Table 3). The only nonsignificant comparison was between Central Asian and European groups for *H_E_* (*P* = 0.898). Unique allele richness was between two and five times higher in the Central Asian group than in any other group. Outside Central Asia, variation measures tended to show the highest variation in Europe (*A* = 4.82, *H_E_* = 0.66, *n_ua_* = 0.66); intermediate levels of variation in North America (*A* = 4.23, *H_E_* = 0.59, *n_ua_* = 0.67); and the lowest variation in Morocco, Brazil, South Africa, and New Zealand (*A*≤3.71, *H_E_*≤0.55, *n_ua_*≤0.14).

**Table 3:** Polymorphism summary for regional groupings of *Venturia inaequalis* samples based on standardization to a group size of 12 individuals.

Regions^5^	*A* 1,5	*SD* 4	*H_E_* 2,5	*SD* 4	*n_ua_* 3,5	*SD* 4
Central Asia	5.03 a	0.37	0.65 a	0.03	1.12 a	0.23
Europe	4.82 b	0.33	0.66 a	0.02	0.66 b	0.24
Morocco	3.71 d	0.32	0.55 c	0.04	0.42 c	0.14
North America	4.23 c	0.35	0.59 b	0.03	0.67 b	0.20
Brazil	3.57e	0.27	0.54 c	0.04	0.32 d	0.13
South Africa	3.32 f	0.25	0.50 d	0.03	0.24 e	0.12
New Zealand	2.91 g	0.35	0.47 e	0.06	0.42 c	0.14

1 allelic richness.

2 expected heterozygosity [56].

3 unique allele richness.

4 standard deviation.

5 for each group, values are averaged across loci and across 100 resampled regional datasets of 12 individuals. Values followed by the same letter are not significantly different (post- hoc Tukey test, *P*≤0.05).

Overall, the proportion of haplotypes repeated multiple times was low (Table 2). Thirteen samples had no repeated haplotypes and mean clonal fraction was 5.2%. On average, the clonal fraction was the highest in the Moroccan group (17.3%). The hypothesis of random mating was not rejected in 21 out of the 29 clone-corrected samples analyzed using the *I_A_* statistic (significance level: 0.05).

A hierarchical analysis of molecular variance (AMOVA) was performed to describe the distribution of population substructure at different geographic scales (Table 4). AMOVA revealed that, while most of the variation (88%) was distributed within samples, a significant proportion of the variation was also attributable to differences among regions (8%). Only 4% of the variation was partitioned among samples within regions. When each region was analyzed separately, population subdivision within regions was low, albeit significant, and the same order of magnitude was observed in all regions (*φ_ST_* = 0.027–0.084, *P*<0.001).

**Table 4:** Hierarchical analysis of molecular variance of worldwide samples of *Venturia inaequalis*.

Dataset	Number of samples	Percentage of variation			*φ*-statistics
		Among groups	Among samples within groups	Among samples	
Central Asia	7	…	3	97	*φ_ST_* = 0.027***
Europe	7	…	4	96	*φ_ST_* = 0.040***
Morocco	3	…	8	92	*φ_ST_* = 0.084***
North America	4	…	4	96	*φ_ST_* = 0.041***
Brazil	5	…	4	96	*φ_ST_* = 0.039***
All Regions	26	8	4	88	*φ_CT_* = −0.079***
					*φ_SC_* = −0.040***
					*φ_ST_* = −0.115***

Only regions with more than two samples were included in analyses. Statistical significance of *φ*-statistics was obtained using 1000 random permutations [50].

*** *P*<0.001.

### Demographic history

We used three different approaches to infer the demographic history of *V. inaequalis* populations: the test for expected heterozygosity excess/deficiency implemented in the Bottleneck program [28], the imbalance index Ln *β* [63], and the interlocus *g* statistic [65].

Most samples had more loci exhibiting an expected heterozygosity deficit than an expected heterozygosity excess: 27/29 samples under the SMM and 22/29 samples under the TPM showed a majority of loci with expected heterozygosity deficit (Table 5). A two-sided Wilcoxon signed rank test revealed that 13 (resp. 5) samples exhibited a pattern of expected heterozygosity that deviated significantly from mutation-drift equilibrium under the SMM (resp. TPM). Although significance was not observed for a majority of samples especially for the TPM, which may be more realistic for microsatellite loci, it is clear from the results that the trend is consistent with expectations for recent population expansion. The general lack of significance may be explained by the use of an insufficient number of loci that could compromise the power of the test [28].

**Table 5:** Tests for mutation-drift equilibrium in 29 samples of *Venturia inaequalis*.

Samples	*D/E* a		*g* b	Ln *β* c
	*SMM*	*TPM*		
**Central Asia**				
CN_1,2,3_	10/2**	10/2**	0.75	2.53* (1.60–3.28)
CN_4_	11/1**	10/2**	0.75	2.42* (1.56–3.10)
CN_5,6,7_	12/0***	12/0***	0.78	2.58* (1.61–3.33)
IR_1_	7/5	7/5	0.60	2.82* (1.96–3.50)
IR_2_	10/2**	8/4	0.64	2.79* (1.78–3.57)
IR_3_	10/2**	9/3	0.81	2.61* (1.84–3.22)
AZ	8/4	8/4	0.44	2.50* (1.43–3.38)
Mean±SD			0.68±0.13	2.61±0.15
**Europe**				
F_1_	5/7	5/7	0.65	2.71* (1.89–3.32)
F_2_	8/4	7/5	0.55	2.68* (1.66–3.33)
F_3,6_	10/2**	8/4	0.59	2.74* (1.81–3.52)
F_4,5_	8/4	6/6	0.68	2.55* (1.56–3.29)
F_7,8_	8/4	7/5	1.17	2.81* (1.84–3.47)
SE	8/4	8/4	0.86	2.44* (1.75–2.91)
SP_1,2,3_	10/2**	4/8	0.71	2.43* (1.64–2.94)
Mean±SD			0.75±0.21	2.63±0.15
**Morocco**				
MA_1,2_	8/3*	8/3	1.53	3.93* (3.06–4.64)
MA_3,4_	10/2*	6/6	1.47	3.62* (2.67–4.21)
MA_5_	4/8	2/10	1.66	2.74* (1.73–3.40)
Mean±SD			1.55±0.10	3.45±0.66
**North America**				
US_1,2_	10/2**	10/2*	0.55	2.89* (2.09–3.46)
US_3_	7/5	7/5	0.76	3.20* (2.51–3.69)
US_4_	9/3*	8/4	0.77	3.26* (2.16–3.95)
CA	8/4	8/4	0.69	3.41* (2.75–3.93)
Mean±SD			0.69±0.10	3.17±0.20
**Brazil**				
BR_1_	11/1**	10/2**	1.59	2.75* (1.77–3.53)
BR_2_	8/4	8/4	1.13	3.00* (2.06–3.74)
BR_3,4_	8/4	7/5	0.85	2.92* (2.08–3.49)
BR_5_	10/2*	9/3	0.70	2.93* (2.16–3.55)
BR_6,7_	7/5	5/7	0.74	2.94* (2.08–3.66)
Mean±SD			1.00±0.37	2.91±0.08
**South Africa**				
SA	8/4	7/5	0.36	2.99* (2.46–3.42)
**New Zealand**				
NZ_1_	7/5	7/5	1.46	3.28* (1.69–4.03)
NZ_2_	8/4	6/6	0.67	3.29* (2.43–3.92)
Mean±SD			1.07±0.56	3.30±0.02

a Number of loci exhibiting heterozygosity deficiency (D) or excess (E) under two mutation models: stepwise mutation model (SMM) and two-phase model (TPM) with 30% of multistep changes [28]. Probability of deviation from mutation-drift equilibrium was assessed using a two-sided Wilcoxon test.

b *g* is the interlocus statistic [65]. Significance was determined using the fifth-percentile cutoffs of [66].

c Ln *β* is the imbalance index [63]. To determine whether Ln *β* was significantly different from 1, we computed 95% confidence intervals by bootstrapping over loci.

* *P*<0.05.

** *P*<0.01.

*** *P*<0.001.

The imbalance index Ln *β* was significantly higher than 1 in all samples (Table 5), suggesting that *V. inaequalis* populations have recently expanded following a bottleneck [63]. On average, the imbalance was strongest in samples from Morocco (3.45±0.66 SD), lowest in Europe (2.63±0.15 SD) and Central Asia (2.61±0.15 SD), and intermediate in newfound lands (2.91±0.08 SD in Brazil, 2.99 in South Africa, 3.17±0.20 SD in North America and 3.30±0.02 SD in New Zealand). This result is consistent with the bottleneck event predating population expansion being most ancient in Central Asia and Europe, most recent in Morocco, and intermediate in newfound lands [65].

The interlocus *g* statistic was lower than 1 in 22 samples (Table 5), which is consistent with population expansion [64], but none of the values were low enough to be significant at 0.05 according to Table 1 of Reich et al. [66]. This result may reflect the lower power of the *g* test to detect recent expansions, particularly when variation in mutation rate across loci is extensive [35], [64] as it may be the case with our data set, which combines dinucleotide and trinucleotide loci.

### Clustering and assignment methods

**Figure 2:**
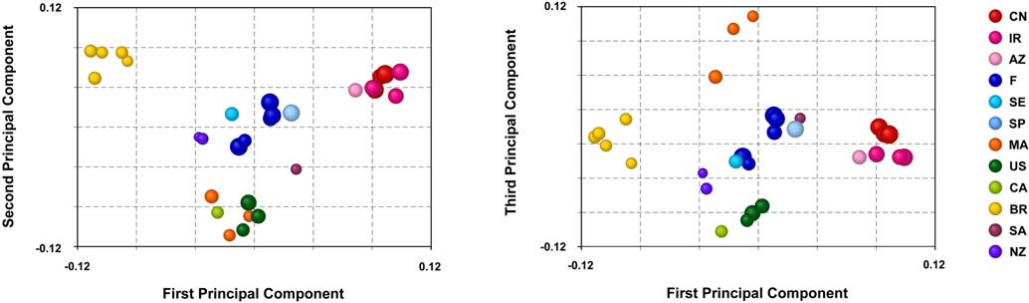
Principal component analysis of a matrix of chord distance [67] among 29 samples of multilocus microsatellite haplotypes of *Venturia inaequalis.* The first, second and third principal components account for 23.2%, 20.9% and 18.7% of variance respectively. For each samples, the diameter of the disk is proportional to allelic richness. CN = China, IR = Iran, AZ = Azerbaijan, F = France, SE = Sweden, SP = Spain, MA = Morocco, US = USA, CA = Canada, BR = Brazil, SA = South Africa, NZ = New Zealand.

In a principal component analysis, the first three principal components accounted for 23.2%, 20.9%, and 18.7% of the variance, respectively. The first two principal components revealed four distinct clusters of samples: a Central Asian cluster, a Brazilian cluster, a cluster formed of Moroccan and North American samples, and a central cluster containing samples from Europe and New Zealand (Figure 2). The third principal component clearly separated Moroccan from North American samples and, along with the first principal component, tended to separate the samples from New Zealand and Europe. The South African sample was placed at the margin of the European cluster in the first and third principal components, and in between the European and North American/Moroccan clusters in the second principal component.

**Figure 3:**
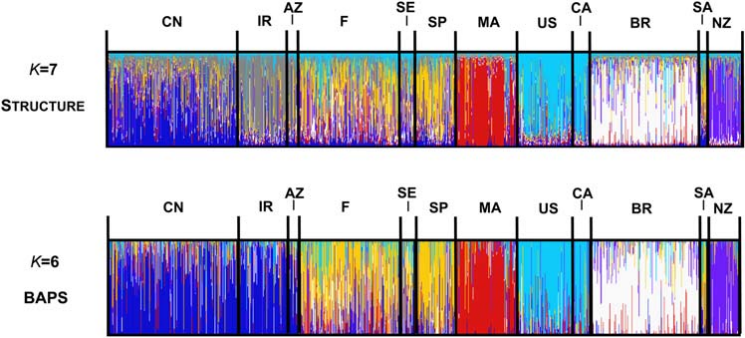
Population structure of *Venturia inaequalis* inferred from 1180 multilocus microsatellite haplotypes using the programs Baps [71] and Structure [69], [70]. Each haplotype is represented by a line partitioned into *K* segments that represent the haplotype's estimated membership fractions in *K* clusters. *K* = 6 and *K* = 7 are the population structure models that best fitted the data using Baps and Structure. CN = China, IR = Iran, AZ = Azerbaijan, F = France, SE = Sweden, SP = Spain, MA = Morocco, US = USA, CA = Canada, BR = Brazil, SA = South Africa, NZ = New Zealand.

Structure analysis was performed without prior information on the geographic origin of samples, with the number of clusters (*K*) varying from 1 to 13. The highest Ln likelihood of the data was obtained for *K* = 7 (Figure S3). The data set was partitioned into clusters corresponding roughly to geography (Figure 3): haplotypes from China, Iran/Azerbaijan, Europe, Morocco, North America, Brazil, and New Zealand tended to be classified in separate clusters. The only noticeable exception was the sample from South Africa, which was assigned in the same cluster as European haplotypes. Overall, individuals from New Zealand, Morocco, Brazil, and North America showed high ancestry fractions in only one group, whereas individuals from Central Asia and Europe/South Africa tended to exhibit more fractional memberships (Figure 3; Table S3).

The clustering algorithm implemented in Baps 4 clearly supported six clusters: five clusters corresponded to the Central Asian, Moroccan, North American, Brazilian, New Zealander groups, and the sixth cluster grouped South African and European samples. Unlike analysis with Structure, Baps did not separate China from Iran/Azerbaijan. Using the admixture analysis implemented in this program, we found lower levels of admixture than with Structure for the same number of clusters (Figure 3, Table S3). As in Structure analysis, the highest levels of admixture were observed in the Central Asian and European/South African groups.

**Figure 4:**
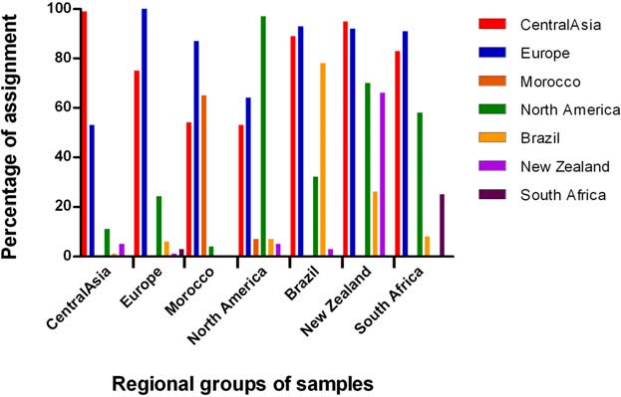
Percentage of haplotypes of *Venturia inaequalis* assigned to 7 regional populations using the assignement method implemented in Geneclass 2 [72].

The exclusion-based method implemented in Geneclass 2 produced an accurate assignment rate of 76% (±27 SD) (Figure 4). The rate of accurate assignment was higher for Central Asian, European, and North American samples (>98%) than for other groups of samples (from 25% in South Africa to 78% in Brazil). Overall, the rate of misassignment was high: many individuals tended to be assigned with high probability in multiple groups, which is consistent with a low level of differentiation among groups [76], [77]. In particular, all groups showed high rates of misassignment in the Central Asian and European groups (on average 75%±18 SD and 81%±17 SD, respectively) and the South African and New Zealander groups displayed high rate of misassignment in the North American group (71% and 58%, respectively).

### Gene flow and effective population size

The migration model with the Central Asian population exchanging migrants only with the European population and unrestricted migration among all non-Central Asian populations (Model 2; Ln(L) = −32266) was found to have significantly higher likelihood than the full model (Model 1; Ln(L) = −37466).

For Model 2, parameter estimates for migration rates and effective population sizes (based on *θ*) varied by population (Table 6). *θ* values indicated that Central Asia (*θ* = 1.12) and Europe (*θ* = 0.91) had higher effective population sizes than Brazil (*θ* = 0.60), North America (*θ* = 0.36), Morocco (*θ* = 0.36), New Zealand (*θ* = 0.19), and South Africa (*θ* = 0.04). This pattern is mostly consistent with what could be expected from measures of allelic richness and expected heterozygosity, except for the Brazilian group that showed unexpectedly high *θ* estimates. Migration rates among regions were generally high (*N_e_m* = 20.2 on average). Parameter estimates revealed that gene flow between Central Asia and Europe was asymmetrical, with more movements westward than eastward. Outside Central Asia, Europe was the main source of immigrants for all populations. Secondary sources of immigrants were Brazil and, to a lesser extent, Morocco and North America, while South Africa and New Zealand acted as sinks.

**Table 6:** Estimates for the mean number of migrants exchanged per generation *M* (2*N_e_m*) and the mean population mutation rate *θ* (2*N_e_μ*) obtained using the program Migrate [75].

Sink populations	*θ*	*M*						
		Source populations						
		Central Asia	Europe	Morocco	North America	Brazil	South Africa	New Zealand
Central Asia	1.12 (1.10–1.14)	…	56.02 (53.66–58.45)	…	…	…	…	…
Europe	0.91 (0.89–0.93)	62.61 (60.07–65.23)	…	14.83 (13.61–16.12)	17.76 (16.42–19.17)	28.89 (27.17–30.68)	1.60 (1.22–2.05)	7.79 (6.91–8.73)
Morocco	0.36 (0.34–0.37)	…	16.73 (15.49–18.03)	…	6.58 (5.81–7.41)	11.56 (10.54–12.65)	1.74 (1.36–2.18)	3.12 (2.61–3.71)
North America	0.36 (0.34–0.37)	…	18.18 (16.94–19.48)	6.03 (5.33–6.79)	…	11.88 (10.88–12.93)	1.33 (1.02–1.71)	2.83 (2.36–3.36)
Brazil	0.60 (0.58–0.62)	…	28.6 (26.97–30.3)	10.94 (9.94–12.00)	12.60 (11.53–13.73)	…	1.43 (1.09–1.83)	5.34 (4.66–6.09)
South Africa	0.04 (0.03–0.05)	…	0.62 (0.50–0.75)	0.44 (0.35–0.55)	0.27 (0.20–0.36)	0.49 (0.39–0.60)	…	0.11 (0.06–0.16)
New Zealand	0.19 (0.18–0.20)	…	7.26 (6.54–8.03)	2.74 (2.31–3.22)	3.31 (2.84–3.84)	5.00 (4.41–5.64)	0.36 (0.22–0.55)	…

*N_e_* is the effective population size, *m* is the migration rate and *μ* is the average mutation rate. Estimates are listed with 95% confidence intervals following in parentheses. The migration model was constrained as follows: the Central Asian population was allowed to exchange migrants with the European population only while all non-Central Asian populations were allowed to exchange migrants without restriction.

## Discussion

### Origin and introduction pathways

We used a multilocus microsatellite typing system to describe the worldwide population genetic structure of the apple scab fungus *V. inaequalis*. Previous studies based on RAPD and PCR-RFLP [43], [44] and microsatellites [45], [48], [49] found high genotypic and genetic diversity in European samples collected on cultivars with no known effective resistance traits. Here, we confirm previous findings at the European scale and we describe the variation in fungal populations from five continents. An older source population is expected to be more variable than a population founded more recently from it [78]. Our finding that genetic variability was higher in Central Asian than in non-Central Asian samples is consistent with a Central Asian origin of the fungus. Just like *Mycosphaerella graminicola*, *Ustilago scitaminea*, *Magnaporthe oryza,* and *Phytophthora infestans*, respectively pathogen on wheat, sugarcane, rice and potato, and unlike the barley pathogen *Rhynchosporium secalis*, *V. inaequalis* seems to share the same geographical origin as its host [21], [61], [79]–[83]. Prospecting and analysis of isolates from Central Asian wild apple should reveal whether the domestication of apple has led to a parallel emergence of apple scab.

The finding of lower levels of variation in non-Central Asian populations suggests that these populations have lost alleles in association with movement, arrival, and establishment outside their native range. However, though less diverse, all these populations were far from being clonal and none displayed extreme reductions in genetic variation such as those reported for other invasive phytopathogenic fungi (e.g., *P. infestans* [84], *P. ramorum* [27], *Sphaeropsis sapinea* [85], *U. scitaminea* [82], *Magnaporthe grisea* [33], *Ceratocystis fimbriata* f. *platani* [26], *Fusarium circinatum* [86]). Rather, the variability observed in *V. inaequalis* samples could be compared with that reported for the cereal pathogens *R. secalis* or *Stagonospora nodorum* outside their centre of origin. [22], [61]. This level of genetic variation points toward multiple introductions, probably in combination with considerable intraregional gene flow and a significant population expansion as host density increased in new apple-growing regions. In particular, European samples displayed a level of variation close to that observed in Central Asia, suggesting that most of the variation from this region has been introduced into Europe during 2,000 years of travel and trade along the Silk Roads. Considering that apple and potentially its pathogen was introduced in North Africa more than 2,000 years ago [42] we could have expected similar levels of variability in the samples of Morocco. Instead, variation in these samples was significantly lower than in Eurasia and more comparable with the variation displayed in newfound lands. Our hypothesis is that this low level of variation can be attributed to subtle changes in the reproductive mode or the epidemiological structure of the fungus because of the particular mild and dry climatic conditions of this area.

Genetic variability was significantly lower in samples from newfound lands than in samples from Eurasia, and variation indices were linearly correlated with geographic distance from Central Asia, indicating that the farthest populations have received a smaller subset of the original variation. This pattern may reflect that the probability of intercontinental movement of infected material has been limited by distance before recent advances in transportation technology and the advent of global trade. In line with this hypothesis, apple was introduced more recently in countries more distant from Central Asia: with the first settlers during the 16^th^ century in North and South America, in 1654 in South Africa, and in 1814 in New Zealand [42].

From our coalescent analyses, it appears likely that *V. inaequalis* followed its host out of Central Asia into Europe, and then into newfound lands. Our migration models indicate that Europe acted as a secondary centre of origin and that very few movements overseas came directly from the actual centre of origin in Central Asia. The intermediate level of variation reported in Europe in comparisons among regions, the central position of European samples in the principal component analysis, and the high rate of misassignment of genotypes from newfound lands in the European group are also consistent with this region being a node in introduction routes.

Exchanges of apple and nursery trees have certainly allowed migrations of *V. inaequalis* among newfound lands [42], [87]. Estimates of gene flow obtained with Migrate or indirectly using the assignment procedure implemented in Geneclass [76] indicated that such migrations have been low in front of the historical contribution of Eurasian populations. Surprisingly, analysis with Migrate indicated that Brazil, but also Morocco and North America, were major sources of immigrants, while South Africa and New Zealand acted as sinks for migration. However, we considered these estimates with caution as the assumption of constant population size is likely to be violated in these populations, which have been recently founded.

In summary, the data available to us are consistent with a model of global co-dispersal of apple and its pathogen: the fungus would have first emerged in Central Asia prior to being introduced into Europe along the Silk Roads and more recently into newfound lands with European colonization.

### Gene flow and population subdivision

We found that most of the genetic variation (88%) was distributed within local samples, which indicates that samples are mostly similar at the genetic level despite distances ranging up to thousands of kilometers. The low differentiation among regions (8% of total variation) can both be explained by levels of gene flow that have been large enough to maintain genetic similarity and/or by insufficient time for differentiation to arise because of recent foundation from a common source. Within regions, the genetic homogeneity observed among samples (4% of total variation) can be explained by the structure of the agrosystem. Apple growing is extensive in all regions where we have sampled and, at least in Central Asia, Europe, and North America, apple trees have covered large areas of land for centuries, both in the form of orchard, meadow, or garden trees [42], [43]. This structure may have been prone to significant levels of man-mediated and natural dispersal of *V. inaequalis*, thereby tending to produce genetic homogeneity among populations within regions.

Note that our study may underestimate population subdivision for two reasons. First, comparative studies have shown that microsatellites could underestimate population differentiation, relative to other markers. The reason given is that the high mutation rate of microsatellite markers increases within-population variance, thereby decreasing the relative magnitude of between-population variance [78], [88]. Second, we found evidence of deviations from mutation-drift equilibrium consistent with a demographic expansion in most populations, and such demographic instabilities are known to downwardly bias estimates of population differentiation [35], [89].

Despite a shallow population structure, clustering analyses partitioned the microsatellite data set into separate groups of samples corresponding roughly to geography. Based on these findings, and considering that “a population (…) is a group of conspecific individuals that are at least relatively genetically isolated and that share a common evolutionary origin” [90], these regional groups of samples can be referred to as distinct *populations*. The only noticeable exception is the South African sample, which clustered in the European group with all three assignment methods. This finding reveals that this population has been founded recently from Europe and that introduction is too recent for quantifiable genetic differences to have developed. Considering that apple scab was reported for the first time in 1888 South Africa [36], populations of more ancient origin probably exist in this region and our sample may not be representative of the real gene pool of *V. inaequalis* in South Africa.

Interestingly, samples from newfound lands tended to have high membership in a single cluster, while many individuals from Eurasia had a high membership in non-Eurasian clusters, though they are supposed to be ancestral to all other regional populations. Similar patterns of admixture among ancestral and derived populations have been reported for other organisms (e.g., *Drosophila melanogaster* [91] or *D. simulans* [92]). Back migrations of individuals from newfound lands into Eurasia could explain this signal of admixture, but it may also simply reflect the recent foundation of newfound lands populations from a Eurasian source, as recent admixture and shared ancestral variation will give the same signal with Bayesian clustering algorithms [92].

### Reproductive biology

Moving a fungus outside its native range can increase linkage disequilibrium or even induce shifts in the reproductive mode because of chance factors (e.g., establishment of only one sexual type) or evolution in response to novel biotic/abiotic conditions [30], [33]. In our study, almost all isolates in all samples had unique haplotypes and the majority of samples did not significantly deviate from expectations under random association of alleles as expected for randomly mating populations. This finding concurs with the prevailing view that sexual recombination is a regular feature of *V. inaequalis* [36] and suggests that our sampling scheme (one isolate per tree) was well suited to avoid collecting clones derived from asexual multiplication. In newfound lands, we would have expected to find more samples with significant multilocus linkage disequilibrium, since multilocus linkage disequilibrium can be inflated by recent foundation and/or admixture between introduced lineages that have different allele frequencies [32]. However, the proportion of samples showing a significant departure from expectations under random association of alleles was not particularly higher in newfound lands (4/12) than in Eurasia (3/14). One explanation is that multiple generations of recombination, segregation, and population growth may have had time to gradually dissipate multilocus linkage disequilibrium in these populations, even though repeated introductions are known to delay the disruption of multilocus linkage disequilibrium [93].

The Moroccan group displayed a singular population structure: all samples had a low level of genetic and genotypic variation and tended to display multilocus linkage disequilibrium. A possible explanation is that the warm and dry climate of that region could curtail the relative contribution of sexual reproduction. A first hypothesis is that winter temperatures may not be low enough to initiate the sexual stage of the fungus [94], [95] and/or to induce the falling of leaves, fruiting bodies being only produced on dead leaves [36]. A second hypothesis is that periodic droughts may have cut down genetic variation across historical times [54]; in dry years, rain-splash and moisture may not be sufficient to produce dense epidemics, resulting in lower population sizes and therefore in higher levels of random genetic drift. Further sampling in North Africa should allow determining whether our samples are representative of the population structure of the fungus in that region, and provide more insights into possible changes in the reproductive mode in connection with climate.

### Demographic history

Tests for mutation-drift equilibrium showed a trend consistent with population expansion in all regions. *Venturia inaequalis* seems to be in the last step of the invasive process: following multiple introductions, the fungus established viable populations, which are now expanding in their novel environment. The imbalance index also indicated that all populations experienced genetic bottlenecks prior to expansion [63], with the bottleneck event being most ancient in Eurasian than in non-Eurasian populations. Eurasian populations may have undergone population shrinkage during the early stages of the putative simultaneous domestication of apple and its fungal pathogen along the Silk Roads [83], while the genetic structure of populations from newfound lands may bear the signature of more recent bottlenecks resulting from founder events. Such patterns of historical changes in demography have already emerged from population genetic analyses of other eukaryotic invasive pathogens (e.g., *M. graminicola* [21], [83] and *Plasmodium falciparum* [96], [97]), and it may be a common feature of many invasive species due to the correlation of their demography with changes in human culture and agricultural practices [35].

## Supporting Information

Table S1 Pairwise PhiST between pairs of samples of V. inaequalis collected on different cultivars in the same location.
(0.01 MB PDF)

Table S2 Origin and MLMT profiles for 1273 isolates of Venturia inaequalis
(0.20 MB PDF)

Table S3 Admixture analysis for 1180 haplotypes of Venturia inaequalis from seven regions: (A) average membership coefficients in seven clusters inferred with the individual-based method implemented in the Structure program [69], [70], and (B) average membership coefficients in six clusters inferred with the sample-based method implemented in Baps 4 [71].
(0.04 MB PDF)

Figure S1 Map of approximate sampling locations.
(0.74 MB PDF)

Figure S2 Scatterplot expected heterozygosity, unique allele richness and arc surface distance from the most eastern sample. A least-square regression line represents the relationship between the two variables. Significance of the correlation was tested using Spearman's r (expected heterozygosity: r = −0.66, P<0.0001; unique allele richness: r = −0.58, P<0.0013).
(0.14 MB PDF)

Figure S3 Plot of Ln likelihood of the data for several value of K, the parameter representing the number of populations in the Bayesian clustering algorithm implemented in the STRUCTURE program [69], [70]. Ln likelihood values were averaged across at least 6 independent runs of the program.
(0.10 MB PDF)

Text S1 Abstract in Chinese, French, Arabic and Portuguese
(0.04 MB DOC)
