# Supplementary material for: On the Origin and Spread of the Scab Disease of Apple: Out of Central Asia
Source: PLoS One. 2008 Jan 16;3(1):e1455. doi: 10.1371/journal.pone.0001455 (PMC2186383; doi:10.1371/journal.pone.0001455)
Supplement: Table S1 — Pairwise PhiST between pairs of samples of V. inaequalis collected on different cultivars in the same location. (0.01 MB PDF) [file pone.0001455.s001.pdf]

Table S1. Pairwise ϕ_{ST} between pairs of samples of *Venturia inaequalis* collected on different cultivars in the same location.

	BR3	BR6	CN1	CN2	CN5	CN6	F3	F4	F7	MA1	MA3	MA4	SP1	SP2	US1
BR4	0.006^{ns}														
BR7		0.039**													
CN2			0.012^{ns}												
CN3			0.005^{ns}	0.000^{ns}											
CN6					0.002^{ns}										
CN7					0.004^{ns}	0.004^{ns}									
F6							0.008^{ns}								
F5								0.012^{ns}							
F8									0.009^{ns}						
MA2										0.000^{ns}					
MA4											0.000^{ns}				
MA5											0.070***	0.086***			
SP2													0.023*		
SP3													0.016 ^{ns}	0.016*	
US2															0.000^{ns}

The null hypothesis $\phi_{ST} = 0$ was tested using 1000 random permutations [50]. $^{ns}P>0.05$, $*P<0.05$, $**P<0.01$, $***P<0.001$.
