# Supplementary material for: On the Origin and Spread of the Scab Disease of Apple: Out of Central Asia
Source: PLoS One. 2008 Jan 16;3(1):e1455. doi: 10.1371/journal.pone.0001455 (PMC2186383; doi:10.1371/journal.pone.0001455)
Supplement: Table S2 — Origin and MLMT profiles for 1273 isolates of Venturia inaequalis (0.20 MB PDF) [file pone.0001455.s002.pdf]

Table S2. Origin and MLMT profiles for 1273 isolates of *Venturia inaequalis*

isolate	sample	host species	host cultivar	country	loci											
					1tc1a	1tc1b	1tc1g	1aac3b	Vitc1/2	Vica9/152	Vitcca7/P	Vitg11/70	Vicacg8/42	M42	Viga7/116	Vica9/X
CN05KB1GO1	CN1	Malus x domestica	Golden Delicious	China	17	15	15	2	7	7	10	5	5	9	4	8
CN05KB1GO10	CN1	Malus x domestica	Golden Delicious	China	10	15	11	2	5	6	9	5	5	9	4	7
CN05KB1GO11	CN1	Malus x domestica	Golden Delicious	China	12	9	8	4	5	7	13	8	5	9	4	7
CN05KB1GO12	CN1	Malus x domestica	Golden Delicious	China	13	21	14	2	7	7	16	11	12	10	4	8
CN05KB1GO13	CN1	Malus x domestica	Golden Delicious	China	11	21	8	2	5	7	?	5	5	8	5	7
CN05KB1GO14	CN1	Malus x domestica	Golden Delicious	China	11	2	9	2	6	6	13	11	8	20	6	8
CN05KB1GO15	CN1	Malus x domestica	Golden Delicious	China	11	15	6	3	7	7	10	10	5	9	6	9
CN05KB1GO18	CN1	Malus x domestica	Golden Delicious	China	?	15	6	2	7	7	16	9	5	9	4	9
CN05KB1GO19	CN1	Malus x domestica	Golden Delicious	China	4	9	?	5	6	7	9	8	14	14	5	7
CN05KB1GO2	CN1	Malus x domestica	Golden Delicious	China	12	9	7	2	13	7	7	14	8	9	4	7
CN05KB1GO20	CN1	Malus x domestica	Golden Delicious	China	5	9	11	3	6	6	6	7	5	9	4	8
CN05KB1GO21	CN1	Malus x domestica	Golden Delicious	China	12	9	17	2	6	7	12	9	2	11	4	9
CN05KB1GO22	CN1	Malus x domestica	Golden Delicious	China	11	9	16	2	11	5	6	8	5	9	4	12
CN05KB1GO23	CN1	Malus x domestica	Golden Delicious	China	9	9	14	2	7	7	7	8	11	9	4	9
CN05KB1GO24	CN1	Malus x domestica	Golden Delicious	China	12	2	11	2	5	6	16	9	12	?	4	7
CN05KB1GO25	CN1	Malus x domestica	Golden Delicious	China	22	10	?	2	5	6	?	8	5	13	5	9
CN05KB1GO26	CN1	Malus x domestica	Golden Delicious	China	7	9	?	2	9	7	10	5	11	12	5	8
CN05KB1GO27	CN1	Malus x domestica	Golden Delicious	China	12	9	13	3	7	7	?	8	5	9	6	7
CN05KB1GO28	CN1	Malus x domestica	Golden Delicious	China	12	9	7	3	11	6	9	7	5	9	6	7
CN05KB1GO29	CN1	Malus x domestica	Golden Delicious	China	13	9	17	2	4	7	16	7	5	9	6	7
CN05KB1GO3	CN1	Malus x domestica	Golden Delicious	China	10	2	3	2	5	6	16	5	5	11	5	7
CN05KB1GO30	CN1	Malus x domestica	Golden Delicious	China	6	8	8	2	4	6	?	6	5	9	6	7
CN05KB1GO31	CN1	Malus x domestica	Golden Delicious	China	10	9	8	2	4	7	8	7	5	9	6	8
CN05KB1GO5	CN1	Malus x domestica	Golden Delicious	China	10	9	4	?	7	8	9	10	5	9	4	7
CN05KB1GO6	CN1	Malus x domestica	Golden Delicious	China	?	11	7	2	5	7	18	13	11	16	6	7
CN05KB1GO7	CN1	Malus x domestica	Golden Delicious	China	?	11	11	2	7	7	8	2	5	16	4	8
CN05KB1GO8	CN1	Malus x domestica	Golden Delicious	China	10	10	8	2	4	7	8	12	5	13	6	7
CN05KB1GO9	CN1	Malus x domestica	Golden Delicious	China	14	2	11	2	4	6	10	6	5	9	6	9
CN05KB2FU10	CN2	Malus x domestica	Fuji	China	12	9	3	2	8	7	8	8	5	10	6	8
CN05KB2FU11	CN2	Malus x domestica	Fuji	China	?	15	5	2	17	7	8	5	?	9	4	8
CN05KB2FU12	CN2	Malus x domestica	Fuji	China	11	11	6	2	14	6	9	3	5	11	5	8
CN05KB2FU13	CN2	Malus x domestica	Fuji	China	15	9	17	2	?	7	?	18	8	10	4	7
CN05KB2FU14	CN2	Malus x domestica	Fuji	China	10	9	22	2	11	7	9	6	8	8	6	7
CN05KB2FU15	CN2	Malus x domestica	Fuji	China	22	9	3	2	5	7	8	5	14	8	5	7
CN05KB2FU16	CN2	Malus x domestica	Fuji	China	22	9	6	4	8	6	13	5	5	9	6	9
CN05KB2FU17	CN2	Malus x domestica	Fuji	China	12	9	?	2	7	7	9	10	12	9	5	8

CN05KB2FU18	CN2	Malus x domestica	Fuji	China	10	9	22	2	13	7	7	6	5	8	6	7
CN05KB2FU19	CN2	Malus x domestica	Fuji	China	?	10	2	2	5	6	9	?	5	16	5	9
CN05KB2FU2	CN2	Malus x domestica	Fuji	China	5	10	?	2	4	7	10	5	5	8	4	8
CN05KB2FU20	CN2	Malus x domestica	Fuji	China	3	9	17	2	5	7	7	10	5	10	5	7
CN05KB2FU21	CN2	Malus x domestica	Fuji	China	12	9	6	4	13	7	9	8	5	8	5	7
CN05KB2FU22	CN2	Malus x domestica	Fuji	China	13	9	11	2	6	7	9	5	4	12	5	9
CN05KB2FU23	CN2	Malus x domestica	Fuji	China	11	2	3	2	7	6	16	5	5	11	7	9
CN05KB2FU24	CN2	Malus x domestica	Fuji	China	6	11	23	2	?	7	17	5	1	10	4	9
CN05KB2FU25	CN2	Malus x domestica	Fuji	China	11	9	1	2	8	7	10	10	12	10	5	7
CN05KB2FU26	CN2	Malus x domestica	Fuji	China	10	9	2	2	7	6	10	8	5	12	5	7
CN05KB2FU27	CN2	Malus x domestica	Fuji	China	3	3	5	2	8	7	13	3	14	12	5	7
CN05KB2FU28	CN2	Malus x domestica	Fuji	China	15	14	?	2	7	7	10	7	4	9	5	8
CN05KB2FU29	CN2	Malus x domestica	Fuji	China	?	10	?	?	7	1	10	6	5	9	4	7
CN05KB2FU3	CN2	Malus x domestica	Fuji	China	9	?	26	2	5	7	9	8	5	8	4	7
CN05KB2FU30	CN2	Malus x domestica	Fuji	China	10	9	11	2	4	7	9	5	5	13	6	7
CN05KB2FU4	CN2	Malus x domestica	Fuji	China	10	9	11	2	7	7	6	7	5	11	5	9
CN05KB2FU5	CN2	Malus x domestica	Fuji	China	3	15	?	2	13	6	8	8	5	9	5	8
CN05KB2FU6	CN2	Malus x domestica	Fuji	China	18	9	11	2	5	7	11	10	5	16	4	8
CN05KB2FU8	CN2	Malus x domestica	Fuji	China	10	9	22	2	11	7	7	6	8	8	6	7
CN05KB2FU9	CN2	Malus x domestica	Fuji	China	13	10	2	2	5	6	10	6	5	9	6	8
CN05KB3GA1	CN3	Malus x domestica	Royal Gala	China	11	9	6	2	7	6	9	7	5	13	4	9
CN05KB3GA10	CN3	Malus x domestica	Royal Gala	China	15	9	?	3	6	7	9	10	5	15	5	8
CN05KB3GA11	CN3	Malus x domestica	Royal Gala	China	18	9	?	2	?	6	7	10	8	9	6	8
CN05KB3GA12	CN3	Malus x domestica	Royal Gala	China	10	9	21	5	4	7	12	8	5	10	9	7
CN05KB3GA13	CN3	Malus x domestica	Royal Gala	China	2	9	11	2	13	1	9	5	5	12	4	8
CN05KB3GA14	CN3	Malus x domestica	Royal Gala	China	5	9	17	5	13	9	9	10	15	13	4	9
CN05KB3GA15	CN3	Malus x domestica	Royal Gala	China	12	9	15	2	5	6	10	8	5	9	6	7
CN05KB3GA16	CN3	Malus x domestica	Royal Gala	China	17	9	?	2	3	7	10	5	5	11	6	7
CN05KB3GA17	CN3	Malus x domestica	Royal Gala	China	11	9	8	2	7	7	9	8	12	9	4	8
CN05KB3GA18	CN3	Malus x domestica	Royal Gala	China	17	9	17	2	12	7	7	5	8	11	5	7
CN05KB3GA19	CN3	Malus x domestica	Royal Gala	China	3	9	15	2	4	7	10	5	5	10	6	7
CN05KB3GA2	CN3	Malus x domestica	Royal Gala	China	19	15	17	2	17	6	7	5	5	9	6	9
CN05KB3GA20	CN3	Malus x domestica	Royal Gala	China	3	?	20	5	7	7	9	8	5	11	5	8
CN05KB3GA21	CN3	Malus x domestica	Royal Gala	China	11	?	6	2	7	6	?	7	5	13	4	9
CN05KB3GA22	CN3	Malus x domestica	Royal Gala	China	3	9	?	2	6	7	4	7	8	10	4	9
CN05KB3GA23	CN3	Malus x domestica	Royal Gala	China	13	15	?	2	8	7	7	?	5	9	6	8
CN05KB3GA24	CN3	Malus x domestica	Royal Gala	China	13	15	17	2	12	7	10	6	5	9	5	7
CN05KB3GA25	CN3	Malus x domestica	Royal Gala	China	15	9	8	2	7	7	7	5	?	13	4	12
CN05KB3GA26	CN3	Malus x domestica	Royal Gala	China	3	6	?	2	2	9	9	8	5	9	5	7
CN05KB3GA27	CN3	Malus x domestica	Royal Gala	China	10	9	8	2	7	6	?	8	5	11	5	8
CN05KB3GA28	CN3	Malus x domestica	Royal Gala	China	19	15	17	2	7	6	10	8	5	9	6	9

CN05KB3GA29	CN3	Malus x domestica	Royal Gala	China	17	9	2	2	11	7	9	9	11	10	4	8
CN05KB3GA3	CN3	Malus x domestica	Royal Gala	China	11	9	6	2	7	6	?	7	5	13	4	9
CN05KB3GA30	CN3	Malus x domestica	Royal Gala	China	17	15	?	2	6	6	9	13	5	6	5	7
CN05KB3GA31	CN3	Malus x domestica	Royal Gala	China	18	14	14	2	?	7	?	8	7	10	7	7
CN05KB3GA32	CN3	Malus x domestica	Royal Gala	China	7	9	14	2	8	7	10	6	12	3	6	7
CN05KB3GA33	CN3	Malus x domestica	Royal Gala	China	13	15	11	2	5	7	9	7	12	12	4	9
CN05KB3GA34	CN3	Malus x domestica	Royal Gala	China	13	9	?	2	7	6	9	8	8	10	5	7
CN05KB3GA35	CN3	Malus x domestica	Royal Gala	China	11	9	11	2	5	6	11	6	5	8	4	8
CN05KB3GA36	CN3	Malus x domestica	Royal Gala	China	10	9	11	2	4	7	9	8	14	16	5	8
CN05KB3GA37	CN3	Malus x domestica	Royal Gala	China	9	9	8	2	7	6	7	6	5	9	5	8
CN05KB3GA38	CN3	Malus x domestica	Royal Gala	China	10	9	8	3	7	7	13	5	5	9	4	8
CN05KB3GA39	CN3	Malus x domestica	Royal Gala	China	3	15	8	2	?	7	8	7	5	9	4	8
CN05KB3GA4	CN3	Malus x domestica	Royal Gala	China	11	9	6	2	7	6	?	7	5	13	4	9
CN05KB3GA40	CN3	Malus x domestica	Royal Gala	China	3	15	6	2	2	2	10	8	5	9	5	7
CN05KB3GA41	CN3	Malus x domestica	Royal Gala	China	17	10	4	2	5	7	11	5	12	3	6	8
CN05KB3GA42	CN3	Malus x domestica	Royal Gala	China	16	2	?	2	?	7	9	5	8	10	4	9
CN05KB3GA43	CN3	Malus x domestica	Royal Gala	China	17	2	?	2	12	7	9	5	11	10	4	9
CN05KB3GA44	CN3	Malus x domestica	Royal Gala	China	12	9	11	2	4	7	9	6	5	10	4	7
CN05KB3GA45	CN3	Malus x domestica	Royal Gala	China	12	10	?	2	9	7	9	6	5	11	5	8
CN05KB3GA48	CN3	Malus x domestica	Royal Gala	China	15	11	3	2	13	7	9	5	11	9	4	9
CN05KB3GA49	CN3	Malus x domestica	Royal Gala	China	13	9	4	2	4	7	7	5	5	9	5	7
CN05KB3GA5	CN3	Malus x domestica	Royal Gala	China	11	9	5	2	7	6	8	4	11	4	4	8
CN05KB3GA50	CN3	Malus x domestica	Royal Gala	China	12	9	11	2	6	7	16	6	5	8	4	7
CN05KB3GA6	CN3	Malus x domestica	Royal Gala	China	11	9	6	2	7	6	?	7	5	13	4	9
CN05KB3GA8	CN3	Malus x domestica	Royal Gala	China	12	2	20	2	12	7	10	8	5	9	5	8
CN05KB3GA9	CN3	Malus x domestica	Royal Gala	China	11	?	6	2	7	6	9	6	5	?	4	9
CN05SG1.1.2	CN4	Malus x domestica	xxx	China	11	9	10	2	13	7	7	5	12	9	6	9
CN05SG1.10.2	CN4	Malus x domestica	xxx	China	13	9	7	2	7	7	7	6	5	10	5	8
CN05SG1.11.1	CN4	Malus x domestica	xxx	China	10	9	11	2	8	7	8	5	5	9	4	8
CN05SG1.12.1	CN4	Malus x domestica	xxx	China	5	9	5	2	8	7	7	7	5	16	4	7
CN05SG1.13.2	CN4	Malus x domestica	xxx	China	11	9	8	2	5	9	?	?	5	10	7	8
CN05SG1.14.2	CN4	Malus x domestica	xxx	China	11	11	2	2	13	7	9	10	12	10	6	9
CN05SG1.15.1	CN4	Malus x domestica	xxx	China	4	9	14	2	7	7	9	7	5	9	5	8
CN05SG1.16.2	CN4	Malus x domestica	xxx	China	5	9	2	2	17	7	7	12	12	15	4	7
CN05SG1.17.2	CN4	Malus x domestica	xxx	China	11	2	6	2	11	7	9	5	5	9	6	8
CN05SG1.18.1	CN4	Malus x domestica	xxx	China	?	?	6	2	5	7	9	5	5	9	5	8
CN05SG1.19.2	CN4	Malus x domestica	xxx	China	11	9	16	2	7	7	9	6	11	9	4	7
CN05SG1.2.2	CN4	Malus x domestica	xxx	China	?	9	1	2	5	7	10	?	5	10	7	8
CN05SG1.20.2	CN4	Malus x domestica	xxx	China	11	9	?	2	6	5	9	5	14	16	5	7
CN05SG1.21.1	CN4	Malus x domestica	xxx	China	6	21	?	2	10	5	?	4	5	20	5	9
CN05SG1.22.2	CN4	Malus x domestica	xxx	China	11	9	7	3	5	7	9	8	5	10	5	9

CN05SG1.23.2	CN4	Malus x domestica	xxx	China	14	14	2	2	7	7	9	5	5	10	5	4
CN05SG1.24.2	CN4	Malus x domestica	xxx	China	?	11	?	2	7	7	9	7	5	10	6	7
CN05SG1.25.2	CN4	Malus x domestica	xxx	China	10	10	8	2	6	7	10	7	5	11	6	7
CN05SG1.26.1	CN4	Malus x domestica	xxx	China	11	21	7	2	5	5	4	8	5	9	5	8
CN05SG1.27.2	CN4	Malus x domestica	xxx	China	9	9	13	2	13	7	9	6	5	10	5	9
CN05SG1.28.1	CN4	Malus x domestica	xxx	China	11	9	8	2	8	7	7	5	5	16	8	9
CN05SG1.29.1	CN4	Malus x domestica	xxx	China	12	?	?	2	7	7	6	7	11	12	4	8
CN05SG1.3.1	CN4	Malus x domestica	xxx	China	7	2	14	2	16	7	7	5	5	10	5	7
CN05SG1.30.1	CN4	Malus x domestica	xxx	China	12	9	6	2	7	9	7	5	?	8	5	7
CN05SG1.31.1	CN4	Malus x domestica	xxx	China	9	14	2	2	9	7	?	6	5	8	5	8
CN05SG1.32.1	CN4	Malus x domestica	xxx	China	13	9	17	2	2	7	9	6	5	10	5	7
CN05SG1.33.1	CN4	Malus x domestica	xxx	China	13	2	18	2	8	7	7	6	5	11	5	7
CN05SG1.34.1	CN4	Malus x domestica	xxx	China	?	?	18	?	17	9	13	12	14	10	5	8
CN05SG1.35.1	CN4	Malus x domestica	xxx	China	13	11	7	4	5	7	9	5	5	9	6	8
CN05SG1.36.1	CN4	Malus x domestica	xxx	China	13	10	17	3	13	5	14	6	5	13	6	8
CN05SG1.37.1	CN4	Malus x domestica	xxx	China	22	9	?	4	13	7	11	5	5	9	6	7
CN05SG1.38.1	CN4	Malus x domestica	xxx	China	10	11	14	2	2	9	10	6	5	9	5	8
CN05SG1.39.1	CN4	Malus x domestica	xxx	China	11	9	8	2	8	7	7	5	5	16	8	9
CN05SG1.4.1	CN4	Malus x domestica	xxx	China	10	9	7	2	7	7	9	8	5	9	7	9
CN05SG1.40.1	CN4	Malus x domestica	xxx	China	10	9	7	2	13	9	7	6	5	9	5	8
CN05SG1.5.1	CN4	Malus x domestica	xxx	China	12	9	14	2	9	7	?	5	5	10	4	8
CN05SG1.6.1	CN4	Malus x domestica	xxx	China	9	9	?	2	5	1	7	8	5	9	4	7
CN05SG1.7.1	CN4	Malus x domestica	xxx	China	13	9	3	2	8	7	7	7	5	15	8	7
CN05SG1.8.1	CN4	Malus x domestica	xxx	China	12	9	4	2	5	7	7	5	5	9	8	7
CN05SG1.9.2	CN4	Malus x domestica	xxx	China	4	11	14	3	7	7	13	9	5	10	7	10
CN05YI2GO1.3	CN5	Malus x domestica	Golden Delicious	China	15	3	18	4	5	7	9	7	5	10	6	7
CN05YI2GO10	CN5	Malus x domestica	Golden Delicious	China	13	11	8	2	5	7	8	11	15	10	4	9
CN05YI2GO11.2	CN5	Malus x domestica	Golden Delicious	China	10	9	14	2	7	7	4	5	5	11	7	7
CN05YI2GO12.2	CN5	Malus x domestica	Golden Delicious	China	13	9	8	2	13	7	9	6	5	10	4	8
CN05YI2GO13.1	CN5	Malus x domestica	Golden Delicious	China	10	2	10	2	11	7	8	10	15	9	4	8
CN05YI2GO14	CN5	Malus x domestica	Golden Delicious	China	16	9	6	2	7	7	8	9	12	3	4	8
CN05YI2GO15	CN5	Malus x domestica	Golden Delicious	China	13	9	13	2	6	7	9	8	15	10	4	7
CN05YI2GO16.2	CN5	Malus x domestica	Golden Delicious	China	13	9	14	2	2	7	9	5	5	9	7	8
CN05YI2GO17	CN5	Malus x domestica	Golden Delicious	China	12	2	?	2	5	7	7	12	5	?	7	7
CN05YI2GO18	CN5	Malus x domestica	Golden Delicious	China	3	9	9	2	7	6	8	5	5	9	7	7
CN05YI2GO2	CN5	Malus x domestica	Golden Delicious	China	9	9	4	2	7	7	8	5	18	12	5	8
CN05YI2GO20	CN5	Malus x domestica	Golden Delicious	China	12	21	6	2	?	7	9	6	5	17	4	7
CN05YI2GO21	CN5	Malus x domestica	Golden Delicious	China	13	9	4	2	13	7	9	7	5	12	5	7
CN05YI2GO22	CN5	Malus x domestica	Golden Delicious	China	15	9	16	2	?	7	?	5	5	11	5	8
CN05YI2GO23	CN5	Malus x domestica	Golden Delicious	China	11	9	14	2	1	7	10	?	15	9	4	8
CN05YI2GO24	CN5	Malus x domestica	Golden Delicious	China	6	14	4	2	6	7	11	5	5	11	4	8

CN05YI2GO25.2	CN5	Malus x domestica	Golden Delicious	China	10	2	10	2	11	7	8	10	15	9	4	8
CN05YI2GO3	CN5	Malus x domestica	Golden Delicious	China	10	11	6	2	6	6	9	12	16	12	4	7
CN05YI2GO4	CN5	Malus x domestica	Golden Delicious	China	16	10	?	2	8	7	9	7	5	12	5	3
CN05YI2GO5.3	CN5	Malus x domestica	Golden Delicious	China	11	9	4	2	7	7	10	5	12	11	4	9
CN05YI2GO6	CN5	Malus x domestica	Golden Delicious	China	9	10	13	2	8	6	9	9	5	9	5	8
CN05YI2GO7.2	CN5	Malus x domestica	Golden Delicious	China	17	21	18	2	7	7	9	6	5	11	5	7
CN05YI2GO8	CN5	Malus x domestica	Golden Delicious	China	3	9	5	2	?	7	9	5	8	10	4	7
CN05YI2GO9	CN5	Malus x domestica	Golden Delicious	China	13	14	14	2	12	7	9	5	5	10	6	7
CN05YI2GO9.2	CN5	Malus x domestica	Golden Delicious	China	13	14	14	2	12	7	9	5	5	10	6	7
CN05YI3NC10	CN6	Malus x domestica	New Century	China	13	9	14	2	5	7	9	12	7	10	5	8
CN05YI3NC14	CN6	Malus x domestica	New Century	China	10	10	17	2	13	7	11	6	8	9	5	8
CN05YI3NC15	CN6	Malus x domestica	New Century	China	12	9	23	2	4	7	10	6	5	9	6	7
CN05YI3NC16	CN6	Malus x domestica	New Century	China	13	9	?	2	5	9	7	12	5	9	6	8
CN05YI3NC17	CN6	Malus x domestica	New Century	China	16	2	6	2	8	9	7	12	5	11	5	8
CN05YI3NC18	CN6	Malus x domestica	New Century	China	5	21	20	2	7	7	?	6	5	9	6	8
CN05YI3NC19	CN6	Malus x domestica	New Century	China	11	21	10	2	7	7	9	3	4	16	5	7
CN05YI3NC22	CN6	Malus x domestica	New Century	China	10	9	8	2	17	7	10	9	5	12	4	8
CN05YI3NC28	CN6	Malus x domestica	New Century	China	12	10	10	2	7	7	8	6	5	8	4	9
CN05YI3NC31	CN6	Malus x domestica	New Century	China	10	10	14	2	6	7	9	8	8	?	4	7
CN05YI3NC32	CN6	Malus x domestica	New Century	China	?	9	5	2	5	7	13	6	5	3	4	8
CN05YI3NC40	CN6	Malus x domestica	New Century	China	11	21	8	2	6	6	11	7	8	20	6	7
CN05YI3NC41	CN6	Malus x domestica	New Century	China	13	10	17	2	5	5	7	2	12	8	5	8
CN05YI3NC42	CN6	Malus x domestica	New Century	China	12	9	8	2	4	7	11	7	5	9	4	7
CN05YI3NC43	CN6	Malus x domestica	New Century	China	3	9	?	2	?	7	9	5	11	3	4	8
CN05YI3NC47	CN6	Malus x domestica	New Century	China	7	9	20	2	6	7	9	6	5	11	7	9
CN05YI3NC50	CN6	Malus x domestica	New Century	China	15	9	16	2	13	7	9	4	2	9	6	7
CN05YI3NC51	CN6	Malus x domestica	New Century	China	13	10	5	2	7	7	13	3	5	4	5	8
CN05YI3NC53	CN6	Malus x domestica	New Century	China	11	2	4	2	?	7	4	10	5	12	6	3
CN05YI3NC55	CN6	Malus x domestica	New Century	China	5	21	2	2	5	7	13	6	5	13	7	8
CN05YI3NC57	CN6	Malus x domestica	New Century	China	12	9	15	3	17	7	9	7	5	11	4	8
CN05YI3NC6	CN6	Malus x domestica	New Century	China	9	2	6	2	23	7	6	7	5	9	4	8
CN05YI3NC61	CN6	Malus x domestica	New Century	China	3	11	4	2	6	7	9	9	9	10	4	7
CN05YI3NC67	CN6	Malus x domestica	New Century	China	12	11	19	3	8	6	9	5	5	11	4	8
CN05YI3NC68	CN6	Malus x domestica	New Century	China	12	9	32	3	?	6	9	2	12	10	4	8
CN05YI3NC71	CN6	Malus x domestica	New Century	China	2	2	20	2	8	7	9	6	9	9	4	8
CN05YI4GA1	CN7	Malus x domestica	Royal Gala	China	17	14	10	2	7	4	13	8	5	7	5	8
CN05YI4GA10	CN7	Malus x domestica	Royal Gala	China	6	9	5	2	13	6	8	13	5	10	6	7
CN05YI4GA11	CN7	Malus x domestica	Royal Gala	China	12	9	4	2	5	9	9	3	8	8	8	7
CN05YI4GA12	CN7	Malus x domestica	Royal Gala	China	6	9	5	2	13	6	8	5	5	10	6	7
CN05YI4GA13	CN7	Malus x domestica	Royal Gala	China	11	4	8	2	5	7	9	12	5	11	8	8
CN05YI4GA14	CN7	Malus x domestica	Royal Gala	China	?	9	10	2	13	7	10	5	2	11	4	2

CN05YI4GA15	CN7	Malus x domestica	Royal Gala	China	12	10	?	3	8	6	11	8	5	13	7	8
CN05YI4GA16	CN7	Malus x domestica	Royal Gala	China	18	10	10	2	5	7	9	7	12	12	8	7
CN05YI4GA17	CN7	Malus x domestica	Royal Gala	China	6	9	5	2	13	6	8	13	5	10	6	7
CN05YI4GA18	CN7	Malus x domestica	Royal Gala	China	13	10	3	2	5	7	?	7	5	10	4	7
CN05YI4GA19	CN7	Malus x domestica	Royal Gala	China	6	?	11	2	8	7	9	5	12	10	4	8
CN05YI4GA2	CN7	Malus x domestica	Royal Gala	China	9	9	10	2	5	7	9	5	5	9	5	7
CN05YI4GA20	CN7	Malus x domestica	Royal Gala	China	6	9	5	2	6	6	9	13	2	10	4	7
CN05YI4GA21	CN7	Malus x domestica	Royal Gala	China	4	11	5	2	5	1	3	6	9	8	4	8
CN05YI4GA22	CN7	Malus x domestica	Royal Gala	China	19	15	10	2	6	7	9	5	2	11	4	8
CN05YI4GA23	CN7	Malus x domestica	Royal Gala	China	11	10	2	2	8	1	10	5	10	10	4	7
CN05YI4GA24	CN7	Malus x domestica	Royal Gala	China	13	10	?	2	6	6	7	7	5	11	8	8
CN05YI4GA25	CN7	Malus x domestica	Royal Gala	China	13	10	10	2	6	7	9	6	5	12	7	?
CN05YI4GA26	CN7	Malus x domestica	Royal Gala	China	6	9	5	2	13	6	8	13	5	10	6	7
CN05YI4GA27	CN7	Malus x domestica	Royal Gala	China	16	9	?	2	8	7	12	6	5	13	4	10
CN05YI4GA28	CN7	Malus x domestica	Royal Gala	China	10	9	19	5	3	3	9	6	8	12	5	8
CN05YI4GA29	CN7	Malus x domestica	Royal Gala	China	13	2	5	2	7	7	9	5	5	9	10	7
CN05YI4GA3	CN7	Malus x domestica	Royal Gala	China	13	9	3	2	11	6	9	5	14	12	8	8
CN05YI4GA30	CN7	Malus x domestica	Royal Gala	China	17	10	6	2	7	9	13	17	4	9	5	8
CN05YI4GA31	CN7	Malus x domestica	Royal Gala	China	13	9	3	2	11	6	9	5	14	12	8	8
CN05YI4GA32	CN7	Malus x domestica	Royal Gala	China	13	10	?	2	7	6	9	7	5	10	4	8
CN05YI4GA33	CN7	Malus x domestica	Royal Gala	China	16	2	27	2	13	7	9	2	5	9	6	7
CN05YI4GA34	CN7	Malus x domestica	Royal Gala	China	10	10	?	2	8	7	9	5	15	?	4	8
CN05YI4GA35	CN7	Malus x domestica	Royal Gala	China	4	2	13	4	7	7	9	8	5	9	6	7
CN05YI4GA37	CN7	Malus x domestica	Royal Gala	China	13	10	21	2	8	7	9	5	8	11	5	7
CN05YI4GA38	CN7	Malus x domestica	Royal Gala	China	10	9	?	2	7	6	10	?	13	11	6	3
CN05YI4GA39	CN7	Malus x domestica	Royal Gala	China	13	10	?	2	6	6	8	7	5	10	4	8
CN05YI4GA4	CN7	Malus x domestica	Royal Gala	China	13	2	3	2	8	7	9	6	12	10	5	7
CN05YI4GA40	CN7	Malus x domestica	Royal Gala	China	14	9	8	2	7	7	11	8	5	9	6	7
CN05YI4GA41	CN7	Malus x domestica	Royal Gala	China	3	10	?	2	5	9	10	5	5	8	11	7
CN05YI4GA42	CN7	Malus x domestica	Royal Gala	China	10	9	?	2	7	?	9	7	8	3	6	9
CN05YI4GA43	CN7	Malus x domestica	Royal Gala	China	12	9	?	2	?	7	7	5	5	11	5	7
CN05YI4GA44	CN7	Malus x domestica	Royal Gala	China	12	9	4	2	?	7	?	5	5	11	4	7
CN05YI4GA5	CN7	Malus x domestica	Royal Gala	China	6	3	4	2	13	6	9	6	5	10	4	8
CN05YI4GA6	CN7	Malus x domestica	Royal Gala	China	6	10	5	2	11	6	9	13	5	?	6	7
CN05YI4GA7	CN7	Malus x domestica	Royal Gala	China	3	9	12	2	6	6	8	12	12	10	4	7
CN05YI4GA8	CN7	Malus x domestica	Royal Gala	China	12	11	14	2	8	7	11	7	5	10	6	8
CN05YI4GA9	CN7	Malus x domestica	Royal Gala	China	19	9	5	2	5	7	9	10	2	12	6	8
CN05YI5GA1	CN7	Malus x domestica	Royal Gala	China	11	9	8	2	5	6	10	2	8	9	6	7
CN05YI5GA10	CN7	Malus x domestica	Royal Gala	China	13	2	?	2	13	7	9	12	9	10	8	8
CN05YI5GA11	CN7	Malus x domestica	Royal Gala	China	14	9	?	2	18	7	7	7	8	14	4	8
CN05YI5GA12	CN7	Malus x domestica	Royal Gala	China	11	9	2	2	5	7	?	6	8	13	6	8

CN05YI5GA13	CN7	Malus x domestica	Royal Gala	China	6	9	2	2	8	6	7	5	5	11	8	7
CN05YI5GA14	CN7	Malus x domestica	Royal Gala	China	6	9	14	2	7	7	14	12	5	8	6	7
CN05YI5GA15	CN7	Malus x domestica	Royal Gala	China	19	2	10	2	7	7	11	5	15	7	4	8
CN05YI5GA16	CN7	Malus x domestica	Royal Gala	China	11	2	13	2	8	6	6	5	8	11	4	9
CN05YI5GA17	CN7	Malus x domestica	Royal Gala	China	3	10	12	2	7	9	7	7	9	11	4	8
CN05YI5GA18	CN7	Malus x domestica	Royal Gala	China	13	11	13	2	13	7	10	7	5	11	6	7
CN05YI5GA2	CN7	Malus x domestica	Royal Gala	China	12	9	6	4	6	7	7	5	8	10	4	8
CN05YI5GA20	CN7	Malus x domestica	Royal Gala	China	8	9	10	2	5	7	10	12	5	6	7	7
CN05YI5GA22	CN7	Malus x domestica	Royal Gala	China	9	19	14	2	5	7	9	12	8	10	5	7
CN05YI5GA23	CN7	Malus x domestica	Royal Gala	China	14	11	10	2	10	7	8	5	1	11	5	7
CN05YI5GA24	CN7	Malus x domestica	Royal Gala	China	11	10	7	4	5	9	9	9	5	10	7	7
CN05YI5GA25	CN7	Malus x domestica	Royal Gala	China	2	9	5	2	8	7	10	9	2	9	6	7
CN05YI5GA26	CN7	Malus x domestica	Royal Gala	China	12	11	10	2	14	6	7	6	5	21	6	9
CN05YI5GA27	CN7	Malus x domestica	Royal Gala	China	13	9	16	2	5	7	9	16	5	10	6	8
CN05YI5GA28	CN7	Malus x domestica	Royal Gala	China	10	9	14	3	5	7	11	8	5	16	4	7
CN05YI5GA29	CN7	Malus x domestica	Royal Gala	China	11	9	10	4	9	7	9	12	5	9	5	8
CN05YI5GA3	CN7	Malus x domestica	Royal Gala	China	6	9	4	2	8	7	8	5	5	9	6	8
CN05YI5GA30	CN7	Malus x domestica	Royal Gala	China	17	9	8	2	5	9	11	8	7	10	4	7
CN05YI5GA4	CN7	Malus x domestica	Royal Gala	China	12	2	6	2	8	7	9	6	7	10	4	8
CN05YI5GA5	CN7	Malus x domestica	Royal Gala	China	11	10	?	3	5	7	?	8	4	10	4	7
CN05YI5GA6	CN7	Malus x domestica	Royal Gala	China	13	10	17	2	?	7	?	6	5	10	5	7
CN05YI5GA7	CN7	Malus x domestica	Royal Gala	China	10	9	?	2	5	7	9	7	5	?	4	8
IR04S1.1b	IR1	Malus x domestica	Golden Delicious	Iran	9	2	10	2	2	7	9	5	5	9	4	?
IR04S1.1.1	IR1	Malus x domestica	Golden Delicious	Iran	14	9	12	3	7	7	7	2	5	12	5	8
IR04S1.10b	IR1	Malus x domestica	Golden Delicious	Iran	12	9	17	?	2	7	9	5	5	11	4	8
IR04S1.10.1	IR1	Malus x domestica	Golden Delicious	Iran	13	9	?	2	7	7	7	2	2	?	4	8
IR04S1.11b	IR1	Malus x domestica	Golden Delicious	Iran	6	9	25	2	5	6	?	13	12	12	4	9
IR04S1.11.1	IR1	Malus x domestica	Golden Delicious	Iran	11	2	17	4	8	7	7	12	5	?	3	8
IR04S1.12b	IR1	Malus x domestica	Golden Delicious	Iran	5	2	4	2	6	7	?	6	12	12	5	8
IR04S1.12.1	IR1	Malus x domestica	Golden Delicious	Iran	6	10	10	2	?	7	7	5	5	?	5	8
IR04S1.13.1	IR1	Malus x domestica	Golden Delicious	Iran	4	9	?	2	17	7	9	5	8	16	7	7
IR04S1.14.1	IR1	Malus x domestica	Golden Delicious	Iran	10	?	?	2	2	7	9	2	5	10	4	9
IR04S1.15.1	IR1	Malus x domestica	Golden Delicious	Iran	6	9	16	2	6	6	8	5	9	11	6	7
IR04S1.16.1	IR1	Malus x domestica	Golden Delicious	Iran	17	?	9	?	17	6	8	5	5	9	8	7
IR04S1.17.1	IR1	Malus x domestica	Golden Delicious	Iran	14	9	16	2	6	?	9	2	5	9	4	?
IR04S1.18.1	IR1	Malus x domestica	Golden Delicious	Iran	15	?	5	2	2	7	7	5	8	?	4	8
IR04S1.19.1	IR1	Malus x domestica	Golden Delicious	Iran	13	?	31	2	2	7	10	17	9	7	10	8
IR04S1.2b	IR1	Malus x domestica	Golden Delicious	Iran	11	9	3	2	2	7	10	12	5	7	4	9
IR04S1.2.1	IR1	Malus x domestica	Golden Delicious	Iran	12	9	12	2	2	7	8	5	5	10	6	8
IR04S1.3b	IR1	Malus x domestica	Golden Delicious	Iran	10	2	5	2	2	7	9	2	5	10	5	7
IR04S1.3.1	IR1	Malus x domestica	Golden Delicious	Iran	14	9	18	2	2	9	7	5	2	5	5	6

IR04S1.4b	IR1	Malus x domestica	Golden Delicious	Iran	12	2	5	2	7	7	7	5	5	16	5	8
IR04S1.4.1	IR1	Malus x domestica	Golden Delicious	Iran	6	9	15	2	?	9	10	5	2	?	6	9
IR04S1.5b	IR1	Malus x domestica	Golden Delicious	Iran	12	10	10	2	2	7	19	11	5	8	4	7
IR04S1.5.1	IR1	Malus x domestica	Golden Delicious	Iran	3	10	?	2	17	7	9	5	5	13	5	8
IR04S1.6b	IR1	Malus x domestica	Golden Delicious	Iran	6	2	6	2	7	7	?	5	5	3	4	7
IR04S1.6.1	IR1	Malus x domestica	Golden Delicious	Iran	11	9	17	2	2	6	10	8	2	9	6	7
IR04S1.7b	IR1	Malus x domestica	Golden Delicious	Iran	17	9	5	3	2	7	8	13	2	9	4	9
IR04S1.7.1	IR1	Malus x domestica	Golden Delicious	Iran	12	2	6	2	8	6	5	8	5	9	4	7
IR04S1.8b	IR1	Malus x domestica	Golden Delicious	Iran	6	9	17	2	2	7	5	6	9	10	4	9
IR04S1.8.1	IR1	Malus x domestica	Golden Delicious	Iran	6	9	10	2	8	?	8	7	5	10	5	?
IR04S1.9b	IR1	Malus x domestica	Golden Delicious	Iran	12	9	16	2	6	7	10	5	8	9	4	8
IR04S1.9.1	IR1	Malus x domestica	Golden Delicious	Iran	5	9	15	2	2	9	9	9	5	4	6	8
IR04S2.1	IR1	Malus x domestica	Golden Delicious	Iran	11	9	6	3	2	7	8	9	5	7	5	?
IR04S2.1.1	IR1	Malus x domestica	Golden Delicious	Iran	4	9	15	2	6	7	8	6	5	13	4	7
IR04S2.10	IR1	Malus x domestica	Golden Delicious	Iran	6	9	9	3	8	7	11	2	9	16	4	9
IR04S2.11	IR1	Malus x domestica	Golden Delicious	Iran	12	9	6	2	2	7	9	7	8	14	4	6
IR04S2.12	IR1	Malus x domestica	Golden Delicious	Iran	6	9	5	2	13	7	11	6	5	5	5	7
IR04S2.2	IR1	Malus x domestica	Golden Delicious	Iran	6	9	14	3	6	7	11	5	5	4	5	8
IR04S2.2.1	IR1	Malus x domestica	Golden Delicious	Iran	15	2	6	2	2	9	8	6	5	4	9	9
IR04S2.3	IR1	Malus x domestica	Golden Delicious	Iran	6	10	6	2	5	7	19	7	5	10	4	7
IR04S2.3.1	IR1	Malus x domestica	Golden Delicious	Iran	26	2	9	2	8	7	19	5	12	10	7	7
IR04S2.4	IR1	Malus x domestica	Golden Delicious	Iran	6	2	2	2	2	7	9	8	5	8	5	8
IR04S2.4.1	IR1	Malus x domestica	Golden Delicious	Iran	6	2	3	2	2	7	5	8	5	?	4	9
IR04S2.5	IR1	Malus x domestica	Golden Delicious	Iran	6	9	17	2	2	7	7	2	2	?	4	?
IR04S2.6	IR1	Malus x domestica	Golden Delicious	Iran	11	10	18	2	2	7	7	2	9	7	4	8
IR04S2.7	IR1	Malus x domestica	Golden Delicious	Iran	6	9	12	3	8	7	?	3	9	16	4	9
IR04S2.8	IR1	Malus x domestica	Golden Delicious	Iran	5	9	3	2	2	7	8	5	6	15	4	9
IR04S2.9	IR1	Malus x domestica	Golden Delicious	Iran	11	2	12	3	2	7	?	5	5	12	5	7
IR04S4.1b	IR2	Malus x domestica	Golden Delicious	Iran	3	10	3	2	6	7	?	7	8	4	7	?
IR04S4.1.1	IR2	Malus x domestica	Golden Delicious	Iran	6	9	3	2	2	6	9	6	2	8	6	7
IR04S4.10b	IR2	Malus x domestica	Golden Delicious	Iran	12	9	18	2	6	7	9	7	6	13	4	9
IR04S4.10.1	IR2	Malus x domestica	Golden Delicious	Iran	6	2	14	2	2	6	12	6	14	11	4	7
IR04S4.11.1	IR2	Malus x domestica	Golden Delicious	Iran	11	9	13	2	2	7	8	5	5	?	4	8
IR04S4.12.1	IR2	Malus x domestica	Golden Delicious	Iran	12	2	21	3	2	7	8	?	5	10	4	9
IR04S4.13.1	IR2	Malus x domestica	Golden Delicious	Iran	18	9	?	2	9	7	9	6	5	9	4	8
IR04S4.14.1	IR2	Malus x domestica	Golden Delicious	Iran	11	9	9	2	7	7	9	6	5	9	4	9
IR04S4.15.1	IR2	Malus x domestica	Golden Delicious	Iran	13	9	6	2	6	6	9	9	3	5	5	8
IR04S4.16.1	IR2	Malus x domestica	Golden Delicious	Iran	14	9	12	2	6	7	10	6	5	6	4	12
IR04S4.17.1	IR2	Malus x domestica	Golden Delicious	Iran	13	9	?	2	6	7	11	2	5	?	5	7
IR04S4.18.1	IR2	Malus x domestica	Golden Delicious	Iran	3	10	3	2	6	7	13	7	8	4	7	7
IR04S4.19.1	IR2	Malus x domestica	Golden Delicious	Iran	19	2	4	2	17	4	11	5	5	7	7	7

IR04S4.2b	IR2	Malus x domestica	Golden Delicious	Iran	6	9	8	3	6	7	?	6	5	11	4	7
IR04S4.20.1	IR2	Malus x domestica	Golden Delicious	Iran	4	2	?	2	2	7	7	5	8	3	6	7
IR04S4.21.1	IR2	Malus x domestica	Golden Delicious	Iran	13	2	2	2	2	7	12	8	5	9	5	8
IR04S4.22.1	IR2	Malus x domestica	Golden Delicious	Iran	12	8	6	2	6	7	9	2	5	14	4	7
IR04S4.3b	IR2	Malus x domestica	Golden Delicious	Iran	9	9	6	2	6	7	7	2	2	8	4	7
IR04S4.3.1	IR2	Malus x domestica	Golden Delicious	Iran	11	9	14	2	2	7	8	5	5	8	4	3
IR04S4.4b	IR2	Malus x domestica	Golden Delicious	Iran	9	?	4	2	7	7	?	6	5	14	4	7
IR04S4.4.1	IR2	Malus x domestica	Golden Delicious	Iran	6	9	6	2	2	6	8	2	8	?	5	12
IR04S4.5b	IR2	Malus x domestica	Golden Delicious	Iran	13	9	8	2	2	7	8	5	9	14	4	7
IR04S4.6b	IR2	Malus x domestica	Golden Delicious	Iran	4	9	12	3	5	7	9	4	?	14	4	7
IR04S4.6.1	IR2	Malus x domestica	Golden Delicious	Iran	13	9	15	2	14	6	8	7	5	12	7	8
IR04S4.7b	IR2	Malus x domestica	Golden Delicious	Iran	9	9	6	2	6	7	7	2	2	8	4	7
IR04S4.7.1	IR2	Malus x domestica	Golden Delicious	Iran	11	9	5	2	2	7	8	6	5	5	3	7
IR04S4.8b	IR2	Malus x domestica	Golden Delicious	Iran	?	9	6	2	2	6	9	2	5	8	5	7
IR04S4.8.1	IR2	Malus x domestica	Golden Delicious	Iran	11	2	18	2	6	7	9	7	5	5	4	9
IR04S4.9b	IR2	Malus x domestica	Golden Delicious	Iran	15	9	20	2	17	7	8	10	9	8	6	8
IR04S4.9b	IR2	Malus x domestica	Golden Delicious	Iran	12	2	6	2	2	7	9	5	5	3	4	7
IR04S4.9.1	IR2	Malus x domestica	Golden Delicious	Iran	12	?	12	2	6	7	9	2	5	?	5	9
IR04S5P1.1	IR3	Malus x domestica	Golden Delicious	Iran	16	9	4	2	12	7	13	10	5	11	8	9
IR04S5P10.1	IR3	Malus x domestica	Golden Delicious	Iran	6	10	14	3	2	7	9	2	8	9	4	8
IR04S5P2.1	IR3	Malus x domestica	Golden Delicious	Iran	6	10	15	3	2	7	7	7	5	9	5	8
IR04S5P3.1	IR3	Malus x domestica	Golden Delicious	Iran	12	9	10	2	6	6	10	5	5	9	5	7
IR04S5P4.1	IR3	Malus x domestica	Golden Delicious	Iran	6	10	9	2	6	?	9	5	5	8	4	?
IR04S5P5.1	IR3	Malus x domestica	Golden Delicious	Iran	13	9	19	2	?	7	7	12	2	?	6	8
IR04S5P7.1	IR3	Malus x domestica	Golden Delicious	Iran	11	?	?	2	8	6	9	6	11	11	4	7
IR04S5P8.1	IR3	Malus x domestica	Golden Delicious	Iran	13	9	9	2	9	7	9	5	8	9	4	8
IR04S5P9.1	IR3	Malus x domestica	Golden Delicious	Iran	6	?	25	?	8	7	10	5	6	16	4	9
IR04S5.10	IR3	Malus x domestica	Golden Delicious	Iran	3	2	5	2	2	7	9	2	8	?	4	6
IR04S5.12	IR3	Malus x domestica	Golden Delicious	Iran	12	5	6	3	6	7	8	10	9	8	6	8
IR04S5.3	IR3	Malus x domestica	Golden Delicious	Iran	1	?	1	2	10	7	?	2	12	4	6	?
IR04S5.4	IR3	Malus x domestica	Golden Delicious	Iran	13	9	7	2	2	7	8	18	5	11	5	7
IR04S5.5	IR3	Malus x domestica	Golden Delicious	Iran	6	9	6	2	2	7	8	5	5	10	4	9
IR04S5.6	IR3	Malus x domestica	Golden Delicious	Iran	13	9	8	2	6	7	7	5	7	9	4	?
IR04S5.7	IR3	Malus x domestica	Golden Delicious	Iran	?	9	16	2	17	7	7	9	9	8	4	?
IR04S5.8	IR3	Malus x domestica	Golden Delicious	Iran	13	9	11	2	2	7	8	5	5	9	4	?
IR04S5.9	IR3	Malus x domestica	Golden Delicious	Iran	?	9	9	2	8	7	6	9	5	9	5	?
AZ05QUSTKR1	AZ	Malus x domestica	Starkrimson	Azerbaijan	13	9	6	2	2	7	8	6	8	10	?	7
AZ05QUSTKR10	AZ	Malus x domestica	Starkrimson	Azerbaijan	11	?	5	3	2	6	7	?	8	?	?	13
AZ05QUSTKR11	AZ	Malus x domestica	Starkrimson	Azerbaijan	11	9	6	2	?	7	7	7	8	9	4	7
AZ05QUSTKR12	AZ	Malus x domestica	Starkrimson	Azerbaijan	13	2	12	2	7	7	?	7	5	?	4	7
AZ05QUSTKR13.1	AZ	Malus x domestica	Starkrimson	Azerbaijan	6	9	?	2	17	7	14	5	8	8	6	9

AZ05QUSTKR14.1	AZ	Malus x domestica	Starkrimson	Azerbaijan	13	9	13	2	2	7	?	9	8	8	4	7
AZ05QUSTKR15.1	AZ	Malus x domestica	Starkrimson	Azerbaijan	6	9	5	2	8	7	11	5	5	10	5	9
AZ05QUSTKR15.2	AZ	Malus x domestica	Starkrimson	Azerbaijan	5	9	4	4	9	7	11	5	4	9	6	7
AZ05QUSTKR16.2	AZ	Malus x domestica	Starkrimson	Azerbaijan	6	2	6	2	2	9	9	4	5	8	8	7
AZ05QUSTKR17.1	AZ	Malus x domestica	Starkrimson	Azerbaijan	6	10	6	2	6	7	6	8	5	11	6	9
AZ05QUSTKR18.2	AZ	Malus x domestica	Starkrimson	Azerbaijan	13	9	8	2	?	6	7	?	8	9	?	8
AZ05QUSTKR2	AZ	Malus x domestica	Starkrimson	Azerbaijan	13	9	16	2	10	6	1	5	5	?	5	8
AZ05QUSTKR3	AZ	Malus x domestica	Starkrimson	Azerbaijan	14	9	13	2	12	7	11	7	5	8	8	11
AZ05QUSTKR4	AZ	Malus x domestica	Starkrimson	Azerbaijan	11	?	7	2	2	7	9	?	15	12	4	8
AZ05QUSTKR5	AZ	Malus x domestica	Starkrimson	Azerbaijan	11	9	?	2	2	7	7	7	5	?	?	7
AZ05QUSTKR6	AZ	Malus x domestica	Starkrimson	Azerbaijan	13	2	?	2	2	7	12	5	8	10	5	8
AZ05QUSTKR7	AZ	Malus x domestica	Starkrimson	Azerbaijan	12	9	6	3	2	9	10	?	5	2	4	7
AZ05QUSTKR8	AZ	Malus x domestica	Starkrimson	Azerbaijan	12	9	?	2	7	7	10	6	6	11	6	8
AZ05QUSTKR9	AZ	Malus x domestica	Starkrimson	Azerbaijan	13	2	?	2	2	7	8	?	8	?	6	8
F05BISLS1	F1	Malus x domestica	Bisquet	France	14	2	7	2	21	6	?	7	5	9	4	8
F05BISLS10	F1	Malus x domestica	Bisquet	France	10	9	?	2	6	6	20	5	5	8	4	8
F05BISLS11	F1	Malus x domestica	Bisquet	France	10	9	7	2	6	7	7	8	8	13	7	8
F05BISLS12	F1	Malus x domestica	Bisquet	France	14	9	21	2	6	7	12	5	5	12	4	7
F05BISLS14	F1	Malus x domestica	Bisquet	France	12	9	?	2	7	7	?	4	?	12	4	8
F05BISLS15	F1	Malus x domestica	Bisquet	France	14	9	17	2	4	9	9	5	5	8	5	8
F05BISLS17	F1	Malus x domestica	Bisquet	France	11	2	19	3	7	6	11	10	2	10	8	8
F05BISLS18	F1	Malus x domestica	Bisquet	France	12	9	14	2	10	6	7	5	5	11	7	12
F05BISLS2	F1	Malus x domestica	Bisquet	France	11	2	7	2	21	6	?	?	5	9	4	8
F05BISLS20	F1	Malus x domestica	Bisquet	France	11	9	16	2	7	7	7	5	2	8	6	7
F05BISLS21	F1	Malus x domestica	Bisquet	France	13	2	13	2	10	7	8	5	5	8	4	8
F05BISLS22	F1	Malus x domestica	Bisquet	France	12	9	?	2	4	6	9	4	?	8	4	7
F05BISLS23	F1	Malus x domestica	Bisquet	France	13	9	19	2	14	6	?	?	?	10	4	7
F05BISLS24	F1	Malus x domestica	Bisquet	France	13	9	8	3	21	9	10	?	?	11	4	8
F05BISLS25	F1	Malus x domestica	Bisquet	France	13	9	8	3	21	9	10	8	5	11	4	8
F05BISLS26	F1	Malus x domestica	Bisquet	France	21	2	15	2	6	7	11	8	5	9	4	7
F05BISLS27	F1	Malus x domestica	Bisquet	France	?	10	11	2	?	7	10	5	5	9	4	8
F05BISLS28	F1	Malus x domestica	Bisquet	France	3	10	11	2	?	7	10	5	5	9	4	8
F05BISLS29	F1	Malus x domestica	Bisquet	France	3	9	11	3	?	7	11	4	11	8	3	8
F05BISLS3	F1	Malus x domestica	Bisquet	France	12	9	7	2	12	?	11	6	8	3	4	8
F05BISLS30	F1	Malus x domestica	Bisquet	France	3	9	11	3	?	7	11	?	?	8	4	8
F05BISLS32	F1	Malus x domestica	Bisquet	France	13	10	19	2	10	6	11	?	?	11	6	8
F05BISLS33	F1	Malus x domestica	Bisquet	France	10	9	3	2	6	6	20	6	5	8	4	8
F05BISLS34	F1	Malus x domestica	Bisquet	France	3	9	8	2	10	7	8	10	5	9	7	8
F05BISLS35	F1	Malus x domestica	Bisquet	France	13	9	20	3	7	7	7	11	8	14	6	7
F05BISLS36	F1	Malus x domestica	Bisquet	France	12	9	?	3	13	7	7	7	5	13	6	7
F05BISLS37	F1	Malus x domestica	Bisquet	France	13	10	14	3	6	7	?	?	5	8	4	7

F05BISLS4	F1	Malus x domestica	Bisquet	France	12	2	17	2	4	7	10	5	11	6	6	8
F05BISLS5	F1	Malus x domestica	Bisquet	France	12	9	9	3	13	7	?	7	5	13	6	7
F05BISLS6	F1	Malus x domestica	Bisquet	France	12	9	15	2	7	6	?	9	?	10	4	8
F05BISLS7	F1	Malus x domestica	Bisquet	France	13	10	10	3	6	7	10	5	5	13	4	7
F05BISLS8	F1	Malus x domestica	Bisquet	France	13	9	15	2	6	9	7	?	?	9	4	8
F05BISLS9	F1	Malus x domestica	Bisquet	France	13	9	15	2	6	9	7	7	2	9	4	8
F05FUJLOR1	F2	Malus x domestica	Fuji	France	12	13	?	2	8	9	9	4	8	16	4	2
F05FUJLOR10	F2	Malus x domestica	Fuji	France	3	9	20	2	5	7	6	6	5	17	6	7
F05FUJLOR11	F2	Malus x domestica	Fuji	France	3	9	6	2	8	7	6	4	5	3	6	8
F05FUJLOR12	F2	Malus x domestica	Fuji	France	15	9	29	2	12	7	7	?	11	10	6	12
F05FUJLOR13	F2	Malus x domestica	Fuji	France	13	9	23	2	6	7	10	13	2	10	7	8
F05FUJLOR14	F2	Malus x domestica	Fuji	France	14	9	?	2	8	9	9	5	11	13	4	8
F05FUJLOR15	F2	Malus x domestica	Fuji	France	13	9	11	?	13	7	10	4	5	9	6	8
F05FUJLOR16	F2	Malus x domestica	Fuji	France	9	1	?	2	7	9	10	6	8	11	4	8
F05FUJLOR17	F2	Malus x domestica	Fuji	France	13	8	16	2	7	9	10	6	5	5	6	2
F05FUJLOR18	F2	Malus x domestica	Fuji	France	12	9	10	2	10	9	10	5	5	9	6	12
F05FUJLOR19	F2	Malus x domestica	Fuji	France	6	10	10	2	14	9	10	7	12	1	6	2
F05FUJLOR2	F2	Malus x domestica	Fuji	France	12	9	12	2	7	9	10	4	8	9	6	8
F05FUJLOR20	F2	Malus x domestica	Fuji	France	13	9	17	3	6	7	10	9	5	5	6	2
F05FUJLOR21	F2	Malus x domestica	Fuji	France	16	9	6	2	6	7	6	6	5	9	7	7
F05FUJLOR22	F2	Malus x domestica	Fuji	France	13	2	18	2	7	7	8	11	2	3	4	7
F05FUJLOR23	F2	Malus x domestica	Fuji	France	5	9	?	3	10	6	9	5	14	18	4	5
F05FUJLOR24	F2	Malus x domestica	Fuji	France	13	9	17	2	9	9	9	8	5	7	4	8
F05FUJLOR25	F2	Malus x domestica	Fuji	France	3	9	?	2	9	7	9	6	8	10	5	2
F05FUJLOR26	F2	Malus x domestica	Fuji	France	14	10	11	2	9	6	14	5	5	3	4	7
F05FUJLOR27	F2	Malus x domestica	Fuji	France	17	10	17	3	13	6	10	5	5	19	6	12
F05FUJLOR28	F2	Malus x domestica	Fuji	France	14	9	15	2	6	7	20	4	11	3	6	7
F05FUJLOR3	F2	Malus x domestica	Fuji	France	3	9	15	2	9	6	10	?	5	?	6	2
F05FUJLOR30	F2	Malus x domestica	Fuji	France	12	9	9	3	8	7	10	5	5	10	6	8
F05FUJLOR4	F2	Malus x domestica	Fuji	France	13	6	6	2	7	7	17	4	2	3	6	7
F05FUJLOR5	F2	Malus x domestica	Fuji	France	15	9	13	2	?	9	8	5	16	9	4	2
F05FUJLOR6	F2	Malus x domestica	Fuji	France	?	10	19	6	6	7	11	5	2	10	6	2
F05FUJLOR7	F2	Malus x domestica	Fuji	France	18	9	26	2	13	6	14	9	8	17	7	7
F05FUJLOR8	F2	Malus x domestica	Fuji	France	12	9	18	2	6	7	10	5	8	10	6	12
F05FUJLOR9	F2	Malus x domestica	Fuji	France	11	9	15	2	8	9	12	9	11	9	7	12
F05GOMU100	F3	Malus x domestica	Mutsu	France	14	9	7	2	19	5	9	5	11	18	6	7
F05GOMU101	F3	Malus x domestica	Mutsu	France	12	1	12	2	13	5	10	14	5	9	3	7
F05GOMU102	F3	Malus x domestica	Mutsu	France	?	9	11	2	8	7	11	5	2	10	3	7
F05GOMU103	F3	Malus x domestica	Mutsu	France	13	13	15	2	7	9	10	5	5	13	6	7
F05GOMU104	F3	Malus x domestica	Mutsu	France	11	9	?	2	7	9	9	6	8	13	4	8
F05GOMU105	F3	Malus x domestica	Mutsu	France	13	10	17	2	19	7	10	6	5	13	5	8

F05GOMU106	F3	Malus x domestica	Mutsu	France	13	10	2	2	10	7	11	5	5	13	4	7
F05GOMU107	F3	Malus x domestica	Mutsu	France	12	6	14	2	13	7	11	5	8	22	7	7
F05GOMU108	F3	Malus x domestica	Mutsu	France	13	10	16	2	6	7	9	6	5	9	4	8
F05GOMU109	F3	Malus x domestica	Mutsu	France	9	2	3	2	8	7	9	6	5	13	4	7
F05GOMU110	F3	Malus x domestica	Mutsu	France	25	13	5	3	13	9	?	11	5	17	6	7
F05GOMU111	F3	Malus x domestica	Mutsu	France	12	6	5	2	10	5	9	6	2	9	5	7
F05GOMU112	F3	Malus x domestica	Mutsu	France	5	9	17	2	7	5	9	8	11	9	5	8
F05GOMU113	F3	Malus x domestica	Mutsu	France	12	9	15	2	7	6	14	8	8	14	4	8
F05GOMU114	F3	Malus x domestica	Mutsu	France	9	9	18	2	5	10	9	6	8	3	4	7
F05GOMU115	F3	Malus x domestica	Mutsu	France	14	9	7	2	10	6	?	8	8	3	4	7
F05GOMU116	F3	Malus x domestica	Mutsu	France	?	6	17	2	6	5	7	8	8	9	5	12
F05GOMU117	F3	Malus x domestica	Mutsu	France	16	6	16	2	7	7	10	8	2	13	6	9
F05GOMU118	F3	Malus x domestica	Mutsu	France	16	6	16	2	7	7	10	1	2	13	6	9
F05GOMU119	F3	Malus x domestica	Mutsu	France	11	9	15	2	19	9	7	5	5	8	5	8
F05GOMU120	F3	Malus x domestica	Mutsu	France	3	9	15	2	6	3	11	5	5	9	7	12
F05GOMU121	F3	Malus x domestica	Mutsu	France	3	2	17	2	7	6	11	6	5	13	6	7
F05GOMU122	F3	Malus x domestica	Mutsu	France	15	9	15	4	13	5	8	8	8	9	5	8
F05GOMU93	F3	Malus x domestica	Mutsu	France	12	10	17	2	7	7	12	6	8	10	4	12
F05GOMU94	F3	Malus x domestica	Mutsu	France	12	7	18	2	12	7	14	14	2	11	4	8
F05GOMU95	F3	Malus x domestica	Mutsu	France	12	9	9	2	6	7	7	6	2	10	5	12
F05GOMU96	F3	Malus x domestica	Mutsu	France	?	9	4	2	7	6	11	4	8	3	5	8
F05GOMU97	F3	Malus x domestica	Mutsu	France	13	10	17	2	7	9	9	12	5	9	6	7
F05GOMU98	F3	Malus x domestica	Mutsu	France	12	9	14	2	10	6	11	7	2	15	4	8
F05GOMU99	F3	Malus x domestica	Mutsu	France	13	9	17	2	6	5	7	6	5	9	6	7
F05LHGA46	F4	Malus x domestica	Royal Gala	France	12	9	11	2	8	6	7	11	8	9	6	8
F05LHGA47	F4	Malus x domestica	Royal Gala	France	11	10	14	2	8	9	7	5	16	8	8	7
F05LHGA48	F4	Malus x domestica	Royal Gala	France	10	10	8	2	?	9	9	5	5	9	4	7
F05LHGA49	F4	Malus x domestica	Royal Gala	France	3	2	11	3	10	6	7	8	5	12	4	7
F05LHGA50	F4	Malus x domestica	Royal Gala	France	12	10	11	2	10	7	10	5	5	8	4	8
F05LHGA51	F4	Malus x domestica	Royal Gala	France	13	9	8	3	7	7	9	11	2	10	4	8
F05LHGA52	F4	Malus x domestica	Royal Gala	France	14	9	14	2	6	9	9	4	5	14	6	8
F05LHGA53	F4	Malus x domestica	Royal Gala	France	17	1	14	2	4	6	9	7	2	12	6	8
F05LHGA54	F4	Malus x domestica	Royal Gala	France	?	12	12	2	4	7	9	5	5	8	6	8
F05LHGA55	F4	Malus x domestica	Royal Gala	France	12	9	3	2	8	6	9	9	5	9	7	8
F05LHGA56	F4	Malus x domestica	Royal Gala	France	12	12	11	2	11	7	8	6	8	3	4	8
F05LHGA57	F4	Malus x domestica	Royal Gala	France	14	10	6	2	6	6	9	7	8	5	4	8
F05LHGA58	F4	Malus x domestica	Royal Gala	France	15	9	23	2	21	7	9	6	2	9	6	8
F05LHGA59	F4	Malus x domestica	Royal Gala	France	12	2	14	2	14	7	8	8	7	10	4	7
F05LHGA60	F4	Malus x domestica	Royal Gala	France	14	9	?	2	7	7	9	7	8	15	7	8
F05LHGA61	F4	Malus x domestica	Royal Gala	France	10	9	14	2	8	6	11	5	5	11	4	7
F05LHGA62	F4	Malus x domestica	Royal Gala	France	12	9	8	2	7	9	11	7	5	13	6	8

F05LHGA63	F4	Malus x domestica	Royal Gala	France	10	9	14	2	7	7	10	4	8	?	6	8
F05LHGA64	F4	Malus x domestica	Royal Gala	France	12	10	14	2	21	6	10	9	5	9	4	8
F05LHGA65	F4	Malus x domestica	Royal Gala	France	13	11	?	2	8	7	9	8	5	3	6	7
F05LHGA66	F4	Malus x domestica	Royal Gala	France	11	9	12	2	7	6	7	4	5	8	7	8
F05LHGA67	F4	Malus x domestica	Royal Gala	France	3	9	9	2	7	5	9	7	8	10	5	8
F05LHGA68	F4	Malus x domestica	Royal Gala	France	13	2	8	3	6	7	9	4	11	11	5	7
F05LHGA69	F4	Malus x domestica	Royal Gala	France	12	9	4	2	?	7	8	8	11	9	5	8
F05LHGA70	F4	Malus x domestica	Royal Gala	France	12	2	18	2	7	7	10	5	1	10	6	8
F05LHGA71	F4	Malus x domestica	Royal Gala	France	12	11	4	2	8	6	10	5	11	12	4	8
F05LHGA72	F4	Malus x domestica	Royal Gala	France	12	9	14	3	6	7	8	9	8	4	4	2
F05LHGA73	F4	Malus x domestica	Royal Gala	France	10	9	16	?	8	7	8	5	8	8	5	8
F05LHGA74	F4	Malus x domestica	Royal Gala	France	11	10	16	2	10	7	9	9	5	9	1	2
F05LHGA75	F4	Malus x domestica	Royal Gala	France	13	10	19	2	13	5	9	6	5	9	5	8
F05LHJO76	F5	Malus x domestica	Jonagold	France	12	9	4	2	7	5	?	4	8	15	6	8
F05LHJO77	F5	Malus x domestica	Jonagold	France	11	2	15	2	10	7	9	8	5	13	4	8
F05LHJO78	F5	Malus x domestica	Jonagold	France	?	9	16	2	13	5	11	7	5	15	4	7
F05LHJO79	F5	Malus x domestica	Jonagold	France	12	9	14	2	11	5	?	?	2	3	?	8
F05LHJO80	F5	Malus x domestica	Jonagold	France	12	9	11	2	12	5	8	4	?	10	4	7
F05LHJO81	F5	Malus x domestica	Jonagold	France	15	2	9	2	12	6	9	6	5	9	4	8
F05LHJO82	F5	Malus x domestica	Jonagold	France	12	9	6	3	12	6	9	5	5	8	5	9
F05LHJO83	F5	Malus x domestica	Jonagold	France	11	9	18	2	9	5	9	5	5	16	6	7
F05LHJO84	F5	Malus x domestica	Jonagold	France	13	10	17	2	7	6	10	7	8	9	6	7
F05LHJO85	F5	Malus x domestica	Jonagold	France	21	9	7	2	9	6	8	5	5	10	1	8
F05LHJO86	F5	Malus x domestica	Jonagold	France	21	9	8	2	8	9	?	8	5	3	7	7
F05LHJO87	F5	Malus x domestica	Jonagold	France	12	9	?	2	11	5	?	6	5	12	4	?
F05LHJO88	F5	Malus x domestica	Jonagold	France	12	10	15	2	7	9	10	8	5	9	6	8
F05LHJO89	F5	Malus x domestica	Jonagold	France	3	9	22	2	7	6	9	5	5	5	4	8
F05LHJO90	F5	Malus x domestica	Jonagold	France	12	9	?	2	13	5	7	8	5	9	4	8
F05LHJO91	F5	Malus x domestica	Jonagold	France	14	2	21	2	10	7	9	6	5	9	4	8
F05LHJO92	F5	Malus x domestica	Jonagold	France	23	10	4	2	13	5	?	6	5	10	4	8
F05SMOGOT1	F6	Malus x domestica	Smoothee	France	18	9	7	2	4	9	?	6	5	8	4	2
F05SMOGOT10	F6	Malus x domestica	Smoothee	France	12	9	17	2	9	7	9	7	8	9	4	2
F05SMOGOT11	F6	Malus x domestica	Smoothee	France	13	9	16	2	6	7	9	8	5	10	6	7
F05SMOGOT12	F6	Malus x domestica	Smoothee	France	15	10	5	2	4	7	?	5	8	8	5	7
F05SMOGOT13	F6	Malus x domestica	Smoothee	France	?	?	20	?	7	6	9	11	11	10	6	8
F05SMOGOT14	F6	Malus x domestica	Smoothee	France	14	9	12	2	13	7	8	4	8	12	4	7
F05SMOGOT15	F6	Malus x domestica	Smoothee	France	3	9	6	2	6	7	9	6	8	13	4	8
F05SMOGOT17	F6	Malus x domestica	Smoothee	France	8	9	17	2	10	9	11	6	8	10	4	12
F05SMOGOT18	F6	Malus x domestica	Smoothee	France	3	10	17	2	?	6	11	5	8	14	7	4
F05SMOGOT19	F6	Malus x domestica	Smoothee	France	22	9	5	2	19	7	10	6	12	13	6	8
F05SMOGOT20	F6	Malus x domestica	Smoothee	France	13	9	2	2	8	9	9	9	2	13	4	7

F05SMOGOT3	F6	Malus x domestica	Smoothee	France	12	9	12	2	10	7	8	12	2	11	4	?
F05SMOGOT4	F6	Malus x domestica	Smoothee	France	3	9	2	2	6	7	9	6	2	15	5	8
F05SMOGOT5	F6	Malus x domestica	Smoothee	France	12	10	23	2	10	7	7	7	2	12	4	7
F05SMOGOT6	F6	Malus x domestica	Smoothee	France	12	9	6	2	10	7	9	8	5	5	4	8
F05SMOGOT7	F6	Malus x domestica	Smoothee	France	3	9	17	2	7	7	?	5	8	8	7	7
F05SMOGOT8	F6	Malus x domestica	Smoothee	France	?	?	5	2	13	9	11	7	5	18	4	?
F05SMOGOT9	F6	Malus x domestica	Smoothee	France	3	9	15	2	?	6	?	6	2	7	6	9
F05VAGA1	F7	Malus x domestica	Royal Gala	France	3	10	18	2	7	7	10	4	1	9	6	8
F05VAGA10	F7	Malus x domestica	Royal Gala	France	11	9	18	2	4	7	10	4	2	11	7	8
F05VAGA11	F7	Malus x domestica	Royal Gala	France	13	9	18	2	6	7	10	4	2	3	4	8
F05VAGA12	F7	Malus x domestica	Royal Gala	France	12	9	5	3	4	7	9	6	8	9	4	8
F05VAGA13	F7	Malus x domestica	Royal Gala	France	12	9	19	2	7	7	10	5	5	10	5	8
F05VAGA14	F7	Malus x domestica	Royal Gala	France	12	9	15	2	8	6	12	7	8	11	4	8
F05VAGA15	F7	Malus x domestica	Royal Gala	France	3	10	4	2	6	7	8	8	5	9	4	8
F05VAGA16	F7	Malus x domestica	Royal Gala	France	12	9	4	3	7	7	9	9	2	10	9	8
F05VAGA17	F7	Malus x domestica	Royal Gala	France	3	10	18	2	4	7	10	7	2	10	7	8
F05VAGA18	F7	Malus x domestica	Royal Gala	France	?	2	?	2	6	7	9	5	8	?	4	9
F05VAGA19	F7	Malus x domestica	Royal Gala	France	?	9	?	2	7	7	7	7	5	10	4	8
F05VAGA20	F7	Malus x domestica	Royal Gala	France	?	9	18	2	7	7	13	7	5	10	4	8
F05VAGA22	F7	Malus x domestica	Royal Gala	France	12	10	13	2	7	7	14	4	2	10	4	8
F05VAGA3	F7	Malus x domestica	Royal Gala	France	11	9	13	2	11	6	9	4	11	11	6	8
F05VAGA4	F7	Malus x domestica	Royal Gala	France	10	9	7	3	11	7	8	8	2	9	7	8
F05VAGA5	F7	Malus x domestica	Royal Gala	France	3	10	10	2	4	7	14	7	2	10	7	7
F05VAGA7	F7	Malus x domestica	Royal Gala	France	12	9	13	2	10	7	7	4	8	10	6	8
F05VAGA8	F7	Malus x domestica	Royal Gala	France	12	10	16	3	8	9	8	8	8	12	4	8
F05VAGA9	F7	Malus x domestica	Royal Gala	France	3	10	13	2	4	7	8	4	8	3	4	8
F05VAGO23	F8	Malus x domestica	Golden Delicious	France	13	9	13	2	7	7	10	11	2	10	7	8
F05VAGO24	F8	Malus x domestica	Golden Delicious	France	3	9	13	2	7	7	8	4	2	10	4	8
F05VAGO25	F8	Malus x domestica	Golden Delicious	France	12	9	18	2	4	7	10	7	2	10	7	8
F05VAGO26	F8	Malus x domestica	Golden Delicious	France	3	10	18	2	7	7	9	7	8	9	6	8
F05VAGO27	F8	Malus x domestica	Golden Delicious	France	3	10	18	2	4	7	10	8	2	10	4	8
F05VAGO28	F8	Malus x domestica	Golden Delicious	France	15	10	13	2	8	6	8	5	2	10	4	8
F05VAGO29	F8	Malus x domestica	Golden Delicious	France	3	9	18	3	6	7	8	5	8	10	6	8
F05VAGO30	F8	Malus x domestica	Golden Delicious	France	3	9	18	2	8	6	10	5	8	5	6	8
F05VAGO31	F8	Malus x domestica	Golden Delicious	France	3	10	18	2	7	7	8	4	2	9	6	8
F05VAGO32	F8	Malus x domestica	Golden Delicious	France	3	10	18	2	7	6	8	4	2	9	10	8
F05VAGO33	F8	Malus x domestica	Golden Delicious	France	5	10	7	2	7	7	8	5	5	4	4	8
F05VAGO34	F8	Malus x domestica	Golden Delicious	France	13	9	13	2	7	7	10	4	2	10	7	8
F05VAGO35	F8	Malus x domestica	Golden Delicious	France	3	9	7	2	6	7	10	7	8	9	5	9
F05VAGO36	F8	Malus x domestica	Golden Delicious	France	13	9	5	3	4	7	9	5	5	9	6	8
F05VAGO37	F8	Malus x domestica	Golden Delicious	France	16	9	18	2	10	6	10	8	8	9	6	8

F05VAGO38	F8	Malus x domestica	Golden Delicious	France	12	9	7	2	6	6	8	8	8	9	6	8
F05VAGO39	F8	Malus x domestica	Golden Delicious	France	3	10	5	3	7	7	9	4	2	9	6	8
F05VAGO40	F8	Malus x domestica	Golden Delicious	France	6	10	18	2	8	7	10	4	5	9	4	8
F05VAGO41	F8	Malus x domestica	Golden Delicious	France	14	9	13	2	6	7	8	8	8	10	6	12
F05VAGO42	F8	Malus x domestica	Golden Delicious	France	3	10	14	2	10	9	8	4	1	9	6	8
F05VAGO43	F8	Malus x domestica	Golden Delicious	France	12	2	18	2	10	7	8	8	8	9	6	8
F05VAGO44	F8	Malus x domestica	Golden Delicious	France	6	10	13	2	4	7	10	5	5	5	6	8
F05VAGO45	F8	Malus x domestica	Golden Delicious	France	?	9	15	3	5	7	8	5	8	10	6	8
SE05ALGA1	SE	Malus x domestica	Royal Gala	Sweden	12	9	15	2	10	6	16	7	8	9	6	7
SE05ALGA1	SE	Malus x domestica	Royal Gala	Sweden	12	9	7	2	11	6	?	6	5	3	6	9
SE05ALGA11.2	SE	Malus x domestica	Royal Gala	Sweden	3	9	5	3	13	7	9	5	8	9	10	9
SE05ALGA12	SE	Malus x domestica	Royal Gala	Sweden	12	9	17	3	11	7	10	6	5	10	4	7
SE05ALGA13	SE	Malus x domestica	Royal Gala	Sweden	12	9	17	2	6	7	10	6	8	9	4	8
SE05ALGA14	SE	Malus x domestica	Royal Gala	Sweden	11	10	17	3	?	6	8	?	8	10	5	7
SE05ALGA15	SE	Malus x domestica	Royal Gala	Sweden	12	9	9	2	7	7	9	8	5	8	5	7
SE05ALGA16	SE	Malus x domestica	Royal Gala	Sweden	12	9	?	2	7	7	8	7	5	10	6	8
SE05ALGA17	SE	Malus x domestica	Royal Gala	Sweden	12	9	?	2	7	7	10	5	5	9	6	7
SE05ALGA18	SE	Malus x domestica	Royal Gala	Sweden	13	9	17	3	7	7	12	8	5	5	4	8
SE05ALGA19	SE	Malus x domestica	Royal Gala	Sweden	4	9	16	2	7	7	12	5	5	10	4	?
SE05ALGA2	SE	Malus x domestica	Royal Gala	Sweden	11	9	7	2	7	4	9	6	5	17	4	8
SE05ALGA2	SE	Malus x domestica	Royal Gala	Sweden	11	11	7	2	8	7	?	6	2	8	6	8
SE05ALGA2.2	SE	Malus x domestica	Royal Gala	Sweden	12	9	?	2	6	7	9	5	5	3	8	7
SE05ALGA21	SE	Malus x domestica	Royal Gala	Sweden	3	6	17	3	7	7	9	7	2	?	6	7
SE05ALGA22	SE	Malus x domestica	Royal Gala	Sweden	11	9	15	2	7	9	10	8	5	?	4	7
SE05ALGA23	SE	Malus x domestica	Royal Gala	Sweden	12	10	17	2	14	2	9	6	8	3	6	7
SE05ALGA3	SE	Malus x domestica	Royal Gala	Sweden	7	9	17	2	7	6	8	4	8	5	6	8
SE05ALGA3	SE	Malus x domestica	Royal Gala	Sweden	18	10	7	3	14	6	11	6	8	15	6	7
SE05ALGA4	SE	Malus x domestica	Royal Gala	Sweden	12	9	?	2	11	6	9	6	5	3	6	9
SE05ALGA4	SE	Malus x domestica	Royal Gala	Sweden	12	17	?	2	7	6	?	7	5	9	6	7
SE05ALGA5	SE	Malus x domestica	Royal Gala	Sweden	11	11	7	2	8	7	9	6	8	9	6	8
SE05ALGA5	SE	Malus x domestica	Royal Gala	Sweden	13	10	21	3	7	6	?	2	2	10	6	7
SE05ALGA6	SE	Malus x domestica	Royal Gala	Sweden	3	9	15	2	?	7	?	8	8	10	8	8
SE05ALGA7	SE	Malus x domestica	Royal Gala	Sweden	12	9	?	2	7	7	9	7	5	9	4	7
SE05ALGA8	SE	Malus x domestica	Royal Gala	Sweden	13	9	13	2	14	6	8	2	8	11	4	7
SP05GA24	SP1	Malus x domestica	Royal Gala	Spain	3	9	22	2	8	6	10	6	5	9	6	7
SP05GA33	SP1	Malus x domestica	Royal Gala	Spain	14	10	18	2	7	6	8	9	8	10	4	8
SP05GA34	SP1	Malus x domestica	Royal Gala	Spain	13	9	15	2	9	9	9	7	5	2	4	8
SP05GA35	SP1	Malus x domestica	Royal Gala	Spain	15	10	9	2	7	9	12	8	5	11	4	8
SP05GA37	SP1	Malus x domestica	Royal Gala	Spain	14	9	7	2	?	6	10	6	5	3	6	8
SP05GA38	SP1	Malus x domestica	Royal Gala	Spain	11	10	17	2	11	7	6	5	8	9	5	7
SP05GA39	SP1	Malus x domestica	Royal Gala	Spain	13	10	9	2	6	6	8	5	5	9	8	8

SP05GA40	SP1	Malus x domestica	Royal Gala	Spain	12	10	?	2	12	7	8	5	5	11	4	8
SP05GA41	SP1	Malus x domestica	Royal Gala	Spain	16	10	14	2	18	6	8	5	5	8	7	8
SP05GA1	SP1	Malus x domestica	Royal Gala	Spain	24	9	?	2	?	7	7	5	5	10	4	?
SP05GA11	SP1	Malus x domestica	Royal Gala	Spain	3	9	22	2	8	6	10	6	5	9	6	7
SP05GA12	SP1	Malus x domestica	Royal Gala	Spain	16	9	?	2	10	6	6	8	5	10	4	7
SP05GA13	SP1	Malus x domestica	Royal Gala	Spain	13	?	8	2	9	6	10	8	5	4	4	7
SP05GA14	SP1	Malus x domestica	Royal Gala	Spain	12	10	17	2	7	6	10	10	11	14	4	8
SP05GA15	SP1	Malus x domestica	Royal Gala	Spain	13	10	8	2	9	7	7	5	8	16	4	8
SP05GA17	SP1	Malus x domestica	Royal Gala	Spain	13	10	9	2	8	6	6	5	5	9	8	8
SP05GA18	SP1	Malus x domestica	Royal Gala	Spain	16	10	14	2	6	6	8	5	5	8	6	?
SP05GA19	SP1	Malus x domestica	Royal Gala	Spain	?	9	18	2	6	7	?	8	5	10	7	7
SP05GA2	SP1	Malus x domestica	Royal Gala	Spain	22	2	14	2	7	6	7	9	5	18	4	7
SP05GA20	SP1	Malus x domestica	Royal Gala	Spain	13	2	27	2	10	7	10	4	5	14	4	8
SP05GA3	SP1	Malus x domestica	Royal Gala	Spain	7	9	?	3	7	6	6	6	8	12	7	8
SP05GA4	SP1	Malus x domestica	Royal Gala	Spain	9	9	14	3	10	9	10	8	2	18	7	7
SP05GA5	SP1	Malus x domestica	Royal Gala	Spain	13	10	8	2	7	6	11	8	5	9	4	8
SP05GA6	SP1	Malus x domestica	Royal Gala	Spain	16	10	14	2	?	6	8	5	5	8	7	?
SP05GA8	SP1	Malus x domestica	Royal Gala	Spain	13	9	8	3	11	6	10	8	5	12	5	7
SP05GA9	SP1	Malus x domestica	Royal Gala	Spain	17	9	9	2	10	6	?	8	5	8	4	8
SP05GO23	SP2	Malus x domestica	Golden Delicious	Spain	15	10	17	3	10	7	8	5	5	3	4	7
SP05GO24	SP2	Malus x domestica	Golden Delicious	Spain	13	10	6	2	10	6	9	6	2	11	5	7
SP05GO25	SP2	Malus x domestica	Golden Delicious	Spain	3	10	3	3	19	6	15	?	8	10	?	8
SP05GO27	SP2	Malus x domestica	Golden Delicious	Spain	15	11	13	2	6	6	9	6	5	8	4	8
SP05GO28	SP2	Malus x domestica	Golden Delicious	Spain	15	10	17	3	10	?	8	5	5	3	4	7
SP05GO31	SP2	Malus x domestica	Golden Delicious	Spain	12	9	17	2	7	7	10	6	5	12	5	8
SP05GO33	SP2	Malus x domestica	Golden Delicious	Spain	12	10	?	2	10	7	9	5	5	9	4	7
SP05GO34	SP2	Malus x domestica	Golden Delicious	Spain	19	9	19	2	10	7	6	6	8	12	6	8
SP05GO35	SP2	Malus x domestica	Golden Delicious	Spain	19	9	19	2	10	7	6	6	8	12	6	8
SP05GO36	SP2	Malus x domestica	Golden Delicious	Spain	12	13	15	2	?	7	10	5	8	9	4	7
SP05GO37	SP2	Malus x domestica	Golden Delicious	Spain	13	10	19	2	?	7	10	5	5	14	5	7
SP05GO38	SP2	Malus x domestica	Golden Delicious	Spain	3	?	8	2	16	7	10	5	6	3	7	8
SP05GO39	SP2	Malus x domestica	Golden Delicious	Spain	12	9	15	2	10	4	10	11	2	10	4	8
SP05GO40	SP2	Malus x domestica	Golden Delicious	Spain	12	9	18	3	6	6	9	10	5	14	4	8
SP05GO41	SP2	Malus x domestica	Golden Delicious	Spain	11	10	16	2	5	7	6	7	8	9	4	8
SP05GO42	SP2	Malus x domestica	Golden Delicious	Spain	13	10	16	2	10	9	11	7	8	4	4	2
SP05Gold1	SP2	Malus x domestica	Golden Delicious	Spain	16	9	20	2	10	9	15	7	5	10	4	?
SP05Gold11	SP2	Malus x domestica	Golden Delicious	Spain	3	?	13	3	7	9	9	5	5	?	4	7
SP05Gold12	SP2	Malus x domestica	Golden Delicious	Spain	13	2	27	2	10	7	10	4	5	14	4	8
SP05Gold13	SP2	Malus x domestica	Golden Delicious	Spain	13	2	17	3	10	7	6	4	2	10	5	8
SP05Gold16	SP2	Malus x domestica	Golden Delicious	Spain	12	?	15	2	7	6	8	4	8	?	4	8
SP05Gold17	SP2	Malus x domestica	Golden Delicious	Spain	3	9	13	2	7	9	9	5	5	13	4	?

SP05Gold18	SP2	Malus x domestica	Golden Delicious	Spain	13	10	18	3	10	9	6	5	2	11	6	2
SP05Gold19	SP2	Malus x domestica	Golden Delicious	Spain	15	10	17	3	10	?	8	5	5	3	4	7
SP05Gold2	SP2	Malus x domestica	Golden Delicious	Spain	3	13	22	2	6	7	10	5	8	9	4	8
SP05Gold20	SP2	Malus x domestica	Golden Delicious	Spain	9	9	23	?	?	7	8	7	5	9	4	7
SP05Gold3	SP2	Malus x domestica	Golden Delicious	Spain	13	10	13	2	6	6	11	4	5	9	4	7
SP05Gold4	SP2	Malus x domestica	Golden Delicious	Spain	13	10	6	2	10	6	9	4	2	11	4	7
SP05Gold5	SP2	Malus x domestica	Golden Delicious	Spain	12	9	18	3	6	6	9	10	5	14	4	8
SP05Gold6	SP2	Malus x domestica	Golden Delicious	Spain	13	10	6	2	10	6	10	4	2	11	4	7
SP05Gold7	SP2	Malus x domestica	Golden Delicious	Spain	12	9	8	3	6	6	11	6	8	10	2	8
SP05Gold9	SP2	Malus x domestica	Golden Delicious	Spain	13	9	17	2	7	9	8	6	8	8	5	7
SP05WE10	SP3	Malus x domestica	Wellspur	Spain	13	10	11	2	7	6	11	6	2	10	4	8
SP05WE11	SP3	Malus x domestica	Wellspur	Spain	10	10	?	2	8	9	8	8	11	5	4	7
SP05WE12.1	SP3	Malus x domestica	Wellspur	Spain	14	?	14	2	8	10	9	4	5	5	6	8
SP05WE12.2	SP3	Malus x domestica	Wellspur	Spain	14	?	14	2	8	?	9	4	5	5	5	8
SP05WE13	SP3	Malus x domestica	Wellspur	Spain	3	9	14	2	7	7	11	8	5	10	5	7
SP05WE14	SP3	Malus x domestica	Wellspur	Spain	10	9	14	2	7	7	8	12	8	8	4	7
SP05WE15	SP3	Malus x domestica	Wellspur	Spain	14	9	17	3	6	7	15	4	5	9	4	8
SP05WE16	SP3	Malus x domestica	Wellspur	Spain	5	9	22	3	?	6	8	5	8	12	4	8
SP05WE17	SP3	Malus x domestica	Wellspur	Spain	12	10	15	2	16	7	9	8	5	9	5	8
SP05WE19	SP3	Malus x domestica	Wellspur	Spain	10	9	14	2	7	7	9	12	8	8	4	7
SP05WE20	SP3	Malus x domestica	Wellspur	Spain	13	9	15	2	5	9	9	5	5	3	4	7
SP05WE22	SP3	Malus x domestica	Wellspur	Spain	?	9	19	2	11	6	14	9	5	11	4	8
SP05WE23	SP3	Malus x domestica	Wellspur	Spain	12	9	12	2	10	7	12	5	5	10	4	9
SP05WE24	SP3	Malus x domestica	Wellspur	Spain	12	10	17	2	7	7	10	6	8	4	5	7
SP05WE27	SP3	Malus x domestica	Wellspur	Spain	12	9	15	2	4	7	8	6	8	7	4	8
SP05WE28	SP3	Malus x domestica	Wellspur	Spain	12	8	17	2	14	6	8	8	2	10	4	8
SP05WE29	SP3	Malus x domestica	Wellspur	Spain	?	9	18	2	4	9	8	6	8	10	5	8
SP05WE32	SP3	Malus x domestica	Wellspur	Spain	13	11	17	2	7	9	11	5	8	9	7	8
SP05WE61	SP3	Malus x domestica	Wellspur	Spain	16	10	10	2	?	9	7	7	5	11	4	7
SP05WE7	SP3	Malus x domestica	Wellspur	Spain	21	10	9	2	7	7	8	7	5	9	4	8
SP05WE81	SP3	Malus x domestica	Wellspur	Spain	13	9	6	2	8	6	10	5	2	9	5	8
SP05WE82	SP3	Malus x domestica	Wellspur	Spain	13	9	6	2	8	6	10	5	2	9	7	8
SP05WE91	SP3	Malus x domestica	Wellspur	Spain	18	9	16	2	22	7	10	8	5	9	5	7
SP05WE92	SP3	Malus x domestica	Wellspur	Spain	13	9	14	2	7	7	11	6	2	8	5	8
MA05S13.9.1	MA1	Malus x domestica	Chimère	Morocco	3	9	22	2	4	5	9	8	11	9	4	8
MA05S13.9	MA1	Malus x domestica	Chimère	Morocco	11	9	12	2	9	7	8	6	5	10	4	8
MA05S13.8.2	MA1	Malus x domestica	Chimère	Morocco	3	9	6	2	4	5	?	6	5	11	4	?
MA05S13.8.1	MA1	Malus x domestica	Chimère	Morocco	3	9	21	2	4	5	12	8	11	9	4	8
MA05S13.8	MA1	Malus x domestica	Chimère	Morocco	3	9	6	2	12	7	10	8	16	3	5	8
MA05S13.7.1	MA1	Malus x domestica	Chimère	Morocco	3	10	?	2	8	7	10	7	5	8	4	2
MA05S13.7	MA1	Malus x domestica	Chimère	Morocco	3	9	6	2	12	5	10	8	16	3	5	8

MA05S13.6.1	MA1	Malus x domestica	Chimère	Morocco	3	9	?	2	4	5	10	8	11	9	4	8
MA05S13.6	MA1	Malus x domestica	Chimère	Morocco	3	9	6	2	12	5	10	8	16	3	5	8
MA05S13.5	MA1	Malus x domestica	Chimère	Morocco	12	9	6	2	4	5	10	6	5	11	4	8
MA05S13.4	MA1	Malus x domestica	Chimère	Morocco	11	9	16	2	9	9	?	6	5	3	4	7
MA05S13.3	MA1	Malus x domestica	Chimère	Morocco	3	9	21	2	4	5	10	8	11	9	4	8
MA05S13.2	MA1	Malus x domestica	Chimère	Morocco	12	2	6	2	4	5	10	6	5	11	4	8
MA05S13.19.1	MA1	Malus x domestica	Chimère	Morocco	16	9	21	2	9	5	10	8	5	9	4	8
MA05S13.18.1	MA1	Malus x domestica	Chimère	Morocco	12	9	6	2	4	5	10	6	5	11	4	8
MA05S13.17.1	MA1	Malus x domestica	Chimère	Morocco	12	9	6	2	4	5	10	6	5	11	4	8
MA05S13.16.1	MA1	Malus x domestica	Chimère	Morocco	3	?	21	?	4	5	10	8	11	?	4	8
MA05S13.15.1	MA1	Malus x domestica	Chimère	Morocco	3	9	21	2	4	5	10	8	16	11	5	8
MA05S13.14.1	MA1	Malus x domestica	Chimère	Morocco	3	9	6	2	12	5	10	8	5	3	5	8
MA05S13.13.1	MA1	Malus x domestica	Chimère	Morocco	3	9	6	2	12	5	?	8	16	3	5	?
MA05S13.12.1	MA1	Malus x domestica	Chimère	Morocco	12	10	17	2	9	6	11	8	2	3	4	7
MA05S13.11.1	MA1	Malus x domestica	Chimère	Morocco	3	9	21	2	4	9	10	6	16	11	5	8
MA05S13.11	MA1	Malus x domestica	Chimère	Morocco	3	9	15	2	8	6	?	8	5	11	4	8
MA05S13.10.1	MA1	Malus x domestica	Chimère	Morocco	11	9	23	2	4	5	10	6	11	11	4	8
MA05S13.10	MA1	Malus x domestica	Chimère	Morocco	16	9	4	2	12	7	?	6	16	9	5	8
MA05S13.1	MA1	Malus x domestica	Chimère	Morocco	3	9	6	2	12	5	10	8	16	3	5	8
MA05S11F51L1	MA2	Malus x domestica	Chimère	Morocco	13	9	21	2	9	7	11	7	8	3	4	7
MA05S11F50L1	MA2	Malus x domestica	Chimère	Morocco	12	9	21	2	4	9	?	8	11	11	4	8
MA05S11F49L1	MA2	Malus x domestica	Chimère	Morocco	3	9	19	2	4	6	7	6	5	14	7	8
MA05S11F48L1	MA2	Malus x domestica	Chimère	Morocco	3	9	16	2	12	7	8	6	11	10	5	8
MA05S11F47L1	MA2	Malus x domestica	Chimère	Morocco	11	9	16	2	12	5	10	4	16	3	5	2
MA05S11F46L1	MA2	Malus x domestica	Chimère	Morocco	3	9	19	2	4	6	7	6	5	14	7	8
MA05S11F45L1	MA2	Malus x domestica	Chimère	Morocco	3	9	21	2	4	5	10	8	11	9	4	8
MA05S11F44L1	MA2	Malus x domestica	Chimère	Morocco	3	9	21	2	12	5	10	8	18	11	5	8
MA05S11F43L1	MA2	Malus x domestica	Chimère	Morocco	3	9	6	2	12	5	10	8	16	3	5	8
MA05S11F42L1	MA2	Malus x domestica	Chimère	Morocco	11	9	12	2	9	7	8	6	5	10	4	8
MA05S11F41L1	MA2	Malus x domestica	Chimère	Morocco	3	9	6	2	13	5	10	8	16	3	5	8
MA05S11F40L1	MA2	Malus x domestica	Chimère	Morocco	11	6	21	2	12	5	10	8	5	11	5	8
MA05S11F39L1	MA2	Malus x domestica	Chimère	Morocco	3	10	24	2	8	7	11	7	8	8	4	7
MA05S11F38L1	MA2	Malus x domestica	Chimère	Morocco	3	10	24	2	8	7	11	7	8	8	4	7
MA05S11F37L1	MA2	Malus x domestica	Chimère	Morocco	3	9	21	2	4	5	10	6	11	10	4	8
MA05S11AN6	MA2	Malus x domestica	Anna	Morocco	3	9	6	2	12	5	10	8	16	3	5	8
MA05S11AN5.1	MA2	Malus x domestica	Anna	Morocco	12	9	6	2	4	5	10	6	5	11	4	8
MA05S11AN35	MA2	Malus x domestica	Anna	Morocco	3	9	6	2	4	5	10	6	5	11	4	8
MA05S11AN34	MA2	Malus x domestica	Anna	Morocco	3	9	6	2	4	5	10	6	5	11	4	8
MA05S11AN32	MA2	Malus x domestica	Anna	Morocco	12	9	6	2	4	5	10	6	5	11	4	8
MA05S11AN3.1	MA2	Malus x domestica	Anna	Morocco	3	9	19	2	4	6	7	6	5	14	7	8
MA05S11AN28	MA2	Malus x domestica	Anna	Morocco	13	6	16	2	12	7	10	6	16	10	5	8

MA05S11AN21.1	MA2	Malus x domestica	Anna	Morocco	3	9	16	2	13	5	10	8	8	3	8	8
MA05S11AN20	MA2	Malus x domestica	Anna	Morocco	3	9	6	2	12	5	10	8	16	3	5	8
MA05S11AN19.1	MA2	Malus x domestica	Anna	Morocco	3	10	24	2	8	7	11	7	8	8	4	7
MA05S11AN18.1	MA2	Malus x domestica	Anna	Morocco	3	9	6	2	12	5	10	8	16	3	5	8
MA05S11AN15.1	MA2	Malus x domestica	Anna	Morocco	3	9	6	2	12	5	10	8	8	3	5	8
MA05S11AN13.1	MA2	Malus x domestica	Anna	Morocco	3	9	6	2	12	5	10	8	16	3	5	8
MA05S11AN12	MA2	Malus x domestica	Anna	Morocco	3	9	6	2	12	5	10	8	16	3	5	8
MA05S1AN9	MA3	Malus x domestica	Anna	Morocco	11	9	21	2	4	9	?	6	11	12	4	8
MA05S1AN8	MA3	Malus x domestica	Anna	Morocco	3	6	21	2	9	9	?	8	5	11	4	8
MA05S1AN7	MA3	Malus x domestica	Anna	Morocco	3	9	21	2	4	5	10	6	11	3	4	8
MA05S1AN6	MA3	Malus x domestica	Anna	Morocco	11	9	4	2	4	9	?	8	5	3	4	?
MA05S1AN5	MA3	Malus x domestica	Anna	Morocco	11	9	16	2	9	9	?	6	5	3	3	8
MA05S1AN4	MA3	Malus x domestica	Anna	Morocco	11	6	11	2	4	5	10	4	5	3	4	8
MA05S1AN34	MA3	Malus x domestica	Anna	Morocco	11	9	21	2	6	5	10	9	8	11	4	2
MA05S1AN33.1	MA3	Malus x domestica	Anna	Morocco	11	6	21	2	4	5	10	6	11	3	4	8
MA05S1AN32	MA3	Malus x domestica	Anna	Morocco	14	9	21	2	?	9	10	4	5	3	?	8
MA05S1AN31.1	MA3	Malus x domestica	Anna	Morocco	11	9	4	2	9	9	10	4	5	3	4	8
MA05S1AN3	MA3	Malus x domestica	Anna	Morocco	11	9	21	2	9	5	10	8	5	11	4	7
MA05S1AN29	MA3	Malus x domestica	Anna	Morocco	11	10	16	2	9	5	8	8	11	11	5	7
MA05S1AN28	MA3	Malus x domestica	Anna	Morocco	11	9	20	2	4	9	?	6	11	12	4	8
MA05S1AN27	MA3	Malus x domestica	Anna	Morocco	11	6	21	2	4	5	10	6	11	3	4	8
MA05S1AN26	MA3	Malus x domestica	Anna	Morocco	12	10	22	2	3	9	10	?	11	11	4	8
MA05S1AN25	MA3	Malus x domestica	Anna	Morocco	11	9	21	2	?	5	10	9	8	11	4	2
MA05S1AN24.1	MA3	Malus x domestica	Anna	Morocco	12	10	22	2	3	9	10	8	11	11	4	8
MA05S1AN23	MA3	Malus x domestica	Anna	Morocco	3	9	4	2	4	5	?	4	11	11	4	8
MA05S1AN22	MA3	Malus x domestica	Anna	Morocco	11	9	4	3	4	5	10	9	11	11	4	8
MA05S1AN21.2	MA3	Malus x domestica	Anna	Morocco	11	9	22	2	12	5	6	4	16	3	5	8
MA05S1AN20.1	MA3	Malus x domestica	Anna	Morocco	11	6	21	2	4	5	10	6	11	3	4	8
MA05S1AN2	MA3	Malus x domestica	Anna	Morocco	11	6	21	2	4	5	10	6	5	9	4	8
MA05S1AN19.3	MA3	Malus x domestica	Anna	Morocco	14	9	16	2	7	5	10	6	16	11	5	8
MA05S1AN18.1	MA3	Malus x domestica	Anna	Morocco	11	6	21	2	4	5	10	6	5	9	4	7
MA05S1AN17	MA3	Malus x domestica	Anna	Morocco	?	9	21	3	9	7	?	9	8	11	?	7
MA05S1AN16	MA3	Malus x domestica	Anna	Morocco	11	9	21	2	9	7	11	7	5	8	4	8
MA05S1AN15	MA3	Malus x domestica	Anna	Morocco	12	9	16	2	?	6	?	9	5	11	4	7
MA05S1AN14.1	MA3	Malus x domestica	Anna	Morocco	11	9	21	3	9	5	10	6	5	3	4	8
MA05S1AN13	MA3	Malus x domestica	Anna	Morocco	11	6	21	2	4	5	10	6	5	9	4	8
MA05S1AN12	MA3	Malus x domestica	Anna	Morocco	11	9	21	2	6	5	10	9	8	11	4	2
MA05S1AN11	MA3	Malus x domestica	Anna	Morocco	3	10	21	3	5	7	11	7	11	8	5	8
MA05S1AN10	MA3	Malus x domestica	Anna	Morocco	3	9	21	2	8	5	6	6	11	3	4	7
MA05S1AN1	MA3	Malus x domestica	Anna	Morocco	11	10	21	3	?	7	11	7	11	9	5	8
MA06MBK1DO1	MA4	Malus x domestica	Dorsett	Morocco	3	9	6	2	9	5	13	6	5	11	4	7

MA06MBK1DO11	MA4	Malus x domestica	Dorsett	Morocco	12	9	?	2	6	7	?	9	?	11	6	8
MA06MBK1DO14	MA4	Malus x domestica	Dorsett	Morocco	11	9	20	2	4	9	9	6	11	12	4	8
MA06MBK1DO16	MA4	Malus x domestica	Dorsett	Morocco	11	6	21	2	4	5	10	6	11	3	4	8
MA06MBK1DO17	MA4	Malus x domestica	Dorsett	Morocco	11	9	22	3	?	7	10	6	16	3	6	8
MA06MBK1DO18	MA4	Malus x domestica	Dorsett	Morocco	11	6	20	2	4	5	10	6	11	3	4	8
MA06MBK1DO19	MA4	Malus x domestica	Dorsett	Morocco	?	9	21	2	?	9	?	12	8	3	4	7
MA06MBK1DO2	MA4	Malus x domestica	Dorsett	Morocco	11	6	21	2	?	5	8	6	8	11	?	8
MA06MBK1DO23	MA4	Malus x domestica	Dorsett	Morocco	3	9	21	2	?	9	12	8	8	11	4	8
MA06MBK1DO24	MA4	Malus x domestica	Dorsett	Morocco	3	9	4	2	9	5	11	6	8	8	4	7
MA06MBK1DO26	MA4	Malus x domestica	Dorsett	Morocco	11	9	22	3	4	5	10	6	11	11	4	8
MA06MBK1DO27	MA4	Malus x domestica	Dorsett	Morocco	11	6	21	2	4	5	10	6	11	3	4	8
MA06MBK1DO28	MA4	Malus x domestica	Dorsett	Morocco	11	9	21	3	7	5	11	9	8	12	4	8
MA06MBK1DO3	MA4	Malus x domestica	Dorsett	Morocco	11	9	21	2	4	5	10	9	5	3	6	8
MA06MBK1DO32	MA4	Malus x domestica	Dorsett	Morocco	3	6	21	2	9	5	10	8	11	9	4	8
MA06MBK1DO33	MA4	Malus x domestica	Dorsett	Morocco	11	9	21	2	4	5	10	5	5	9	4	8
MA06MBK1DO34	MA4	Malus x domestica	Dorsett	Morocco	11	9	4	2	9	7	10	6	5	3	4	8
MA06MBK1DO35	MA4	Malus x domestica	Dorsett	Morocco	11	9	22	2	9	5	10	6	5	11	4	?
MA06MBK1DO36	MA4	Malus x domestica	Dorsett	Morocco	11	9	21	2	4	5	10	5	11	3	4	8
MA06MBK1DO37	MA4	Malus x domestica	Dorsett	Morocco	11	9	21	2	9	5	10	5	5	3	4	8
MA06MBK1DO4	MA4	Malus x domestica	Dorsett	Morocco	11	9	21	3	9	5	10	6	5	3	?	8
MA06MBK1DO43	MA4	Malus x domestica	Dorsett	Morocco	11	6	21	2	4	5	10	6	11	3	4	8
MA06MBK1DO44	MA4	Malus x domestica	Dorsett	Morocco	11	6	21	2	4	9	10	4	8	3	4	8
MA06MBK1DO45	MA4	Malus x domestica	Dorsett	Morocco	11	6	18	3	9	9	10	4	5	7	4	8
MA06MBK1DO46	MA4	Malus x domestica	Dorsett	Morocco	3	9	21	2	6	5	10	9	8	11	4	2
MA06MBK1DO48	MA4	Malus x domestica	Dorsett	Morocco	11	9	4	2	6	9	12	4	8	11	4	8
MA06MBK1DO6	MA4	Malus x domestica	Dorsett	Morocco	12	9	6	2	4	5	10	6	5	11	4	8
MA06MBK1DO7	MA4	Malus x domestica	Dorsett	Morocco	3	9	21	2	?	5	10	8	5	11	4	2
MA06MBK2GA10	MA5	Malus x domestica	Royal Gala	Morocco	?	?	4	2	7	9	12	?	8	12	4	8
MA06MBK2GA11	MA5	Malus x domestica	Royal Gala	Morocco	11	10	?	2	7	9	9	10	8	10	6	?
MA06MBK2GA12	MA5	Malus x domestica	Royal Gala	Morocco	3	10	20	2	7	7	11	8	8	11	5	?
MA06MBK2GA13	MA5	Malus x domestica	Royal Gala	Morocco	3	10	21	2	9	5	10	6	5	8	4	7
MA06MBK2GA14	MA5	Malus x domestica	Royal Gala	Morocco	12	9	9	3	?	6	?	?	5	8	6	8
MA06MBK2GA15	MA5	Malus x domestica	Royal Gala	Morocco	12	2	?	2	7	10	8	8	8	9	6	7
MA06MBK2GA16	MA5	Malus x domestica	Royal Gala	Morocco	11	8	20	2	?	5	8	4	8	3	6	8
MA06MBK2GA18	MA5	Malus x domestica	Royal Gala	Morocco	12	9	24	2	4	9	?	7	11	8	4	7
MA06MBK2GA20	MA5	Malus x domestica	Royal Gala	Morocco	11	9	?	2	9	7	?	8	8	5	4	8
MA06MBK2GA21	MA5	Malus x domestica	Royal Gala	Morocco	?	9	?	2	5	10	5	8	?	10	6	8
MA06MBK2GA22	MA5	Malus x domestica	Royal Gala	Morocco	11	2	5	2	5	5	6	?	2	9	6	8
MA06MBK2GA27	MA5	Malus x domestica	Royal Gala	Morocco	12	9	24	2	4	9	8	7	11	8	4	7
MA06MBK2GA3	MA5	Malus x domestica	Royal Gala	Morocco	?	6	21	3	12	9	10	6	5	3	4	8
MA06MBK2GA30	MA5	Malus x domestica	Royal Gala	Morocco	?	6	21	2	?	7	10	8	5	11	4	8

MA06MBK2GA35	MA5	Malus x domestica	Royal Gala	Morocco	11	9	21	2	4	6	4	8	5	3	4	8
MA06MBK2GA38	MA5	Malus x domestica	Royal Gala	Morocco	12	6	21	2	?	9	10	8	8	3	6	7
MA06MBK2GA40	MA5	Malus x domestica	Royal Gala	Morocco	11	6	18	3	9	5	10	8	8	7	4	8
MA06MBK2GA41	MA5	Malus x domestica	Royal Gala	Morocco	11	6	18	3	9	5	10	8	8	7	4	8
MA06MBK2GA42	MA5	Malus x domestica	Royal Gala	Morocco	?	9	?	3	4	9	?	12	8	10	6	8
MA06MBK2GA43	MA5	Malus x domestica	Royal Gala	Morocco	11	10	21	3	4	7	12	9	8	8	6	8
MA06MBK2GA44	MA5	Malus x domestica	Royal Gala	Morocco	12	10	21	3	4	7	12	7	11	8	4	8
MA06MBK2GA5	MA5	Malus x domestica	Royal Gala	Morocco	12	9	?	2	5	9	6	7	5	8	6	7
MA06MBK2GA7	MA5	Malus x domestica	Royal Gala	Morocco	12	2	24	2	5	7	7	8	5	9	6	?
MA06MBK2GA8	MA5	Malus x domestica	Royal Gala	Morocco	11	10	21	3	4	7	11	8	?	8	5	8
MA06MBK2GA9	MA5	Malus x domestica	Royal Gala	Morocco	11	9	6	3	9	5	10	5	5	3	4	8
US06GECO1	US1	Malus x domestica	Cortland	USA	11	9	9	2	21	7	?	5	8	9	6	2
US06GECO11	US1	Malus x domestica	Cortland	USA	11	10	?	2	14	7	8	7	5	6	6	7
US06GECO12	US1	Malus x domestica	Cortland	USA	10	10	22	2	14	6	9	7	11	10	6	2
US06GECO17.2	US1	Malus x domestica	Cortland	USA	3	9	?	2	21	7	8	7	5	9	4	8
US06GECO18	US1	Malus x domestica	Cortland	USA	3	9	?	2	7	7	8	5	8	10	4	7
US06GECO2	US1	Malus x domestica	Cortland	USA	13	18	4	2	2	9	11	7	8	10	6	2
US06GECO20	US1	Malus x domestica	Cortland	USA	5	21	8	2	14	7	?	4	2	6	6	7
US06GECO21	US1	Malus x domestica	Cortland	USA	12	9	15	3	8	9	9	7	5	3	6	2
US06GECO22	US1	Malus x domestica	Cortland	USA	12	9	7	2	6	9	9	7	5	10	6	8
US06GECO23	US1	Malus x domestica	Cortland	USA	3	9	13	3	14	7	8	7	8	9	6	2
US06GECO25	US1	Malus x domestica	Cortland	USA	3	9	7	3	13	7	?	4	5	13	4	8
US06GECO27	US1	Malus x domestica	Cortland	USA	11	9	16	3	2	7	10	4	8	11	6	8
US06GECO28	US1	Malus x domestica	Cortland	USA	12	9	11	2	17	9	?	7	8	7	9	8
US06GECO29	US1	Malus x domestica	Cortland	USA	12	9	7	2	15	7	?	6	8	9	6	2
US06GECO3	US1	Malus x domestica	Cortland	USA	12	9	6	2	18	7	5	4	5	5	6	2
US06GECO36	US1	Malus x domestica	Cortland	USA	12	9	2	2	16	?	7	6	8	10	6	8
US06GECO39	US1	Malus x domestica	Cortland	USA	3	9	3	2	14	7	?	7	5	3	4	12
US06GECO6	US1	Malus x domestica	Cortland	USA	3	10	11	2	14	?	6	5	5	5	6	8
US06GECO8	US1	Malus x domestica	Cortland	USA	3	9	12	2	14	9	8	?	2	11	8	2
US06GEMI1	US2	Malus x domestica	McIntosh	USA	11	10	9	3	7	7	?	7	8	10	6	2
US06GEMI10	US2	Malus x domestica	McIntosh	USA	11	9	8	2	16	7	9	7	8	9	6	2
US06GEMI11	US2	Malus x domestica	McIntosh	USA	11	9	17	3	15	4	9	7	8	10	6	2
US06GEMI11.2	US2	Malus x domestica	McIntosh	USA	3	6	5	2	15	7	7	5	8	10	8	8
US06GEMI12	US2	Malus x domestica	McIntosh	USA	12	9	13	2	7	7	6	4	8	9	6	2
US06GEMI13	US2	Malus x domestica	McIntosh	USA	12	10	2	3	16	9	6	5	8	10	8	2
US06GEMI15	US2	Malus x domestica	McIntosh	USA	14	10	11	3	14	6	9	7	2	10	4	2
US06GEMI16	US2	Malus x domestica	McIntosh	USA	14	10	3	2	17	10	?	4	8	10	6	7
US06GEMI16.2	US2	Malus x domestica	McIntosh	USA	13	9	11	2	6	7	?	7	5	9	6	8
US06GEMI17	US2	Malus x domestica	McIntosh	USA	13	2	8	2	4	7	8	7	5	9	6	2
US06GEMI2	US2	Malus x domestica	McIntosh	USA	11	9	12	2	5	?	9	5	8	5	6	2

US06GEMI20.2	US2	Malus x domestica	McIntosh	USA	12	2	8	2	14	9	10	6	5	6	7	2
US06GEMI21	US2	Malus x domestica	McIntosh	USA	3	9	3	2	14	7	10	5	5	10	6	2
US06GEMI25	US2	Malus x domestica	McIntosh	USA	11	9	7	2	12	7	9	7	5	12	?	2
US06GEMI28	US2	Malus x domestica	McIntosh	USA	11	9	11	2	17	7	10	5	8	3	6	8
US06GEMI29	US2	Malus x domestica	McIntosh	USA	11	9	7	3	2	?	?	4	1	18	6	2
US06GEMI3	US2	Malus x domestica	McIntosh	USA	11	9	16	2	?	7	8	8	5	7	4	8
US06GEMI30	US2	Malus x domestica	McIntosh	USA	11	9	19	3	17	7	?	15	8	11	6	8
US06GEMI33	US2	Malus x domestica	McIntosh	USA	11	10	?	2	15	7	5	7	11	3	7	2
US06GEMI36	US2	Malus x domestica	McIntosh	USA	3	2	17	2	7	7	8	5	8	6	7	2
US06GEMI39	US2	Malus x domestica	McIntosh	USA	11	9	19	2	14	9	8	4	5	9	4	2
US06GEMI4	US2	Malus x domestica	McIntosh	USA	11	9	20	2	8	7	9	4	5	9	4	8
US06GEMI40	US2	Malus x domestica	McIntosh	USA	3	9	?	2	14	7	8	10	5	11	4	2
US06GEMI5	US2	Malus x domestica	McIntosh	USA	3	9	17	2	2	9	8	5	8	14	6	8
US06GEMI8	US2	Malus x domestica	McIntosh	USA	12	10	10	2	17	7	9	4	8	11	6	2
US06GEMI9	US2	Malus x domestica	McIntosh	USA	13	9	16	2	13	7	16	4	5	3	6	8
US06INRD1	US3	Malus x domestica	Red Delicious	USA	?	?	11	?	6	7	10	6	8	9	6	7
US06INRD11	US3	Malus x domestica	Red Delicious	USA	3	9	11	2	21	7	8	7	2	6	6	9
US06INRD14	US3	Malus x domestica	Red Delicious	USA	11	10	9	2	17	7	?	5	5	10	4	9
US06INRD16	US3	Malus x domestica	Red Delicious	USA	20	?	11	2	17	9	6	4	8	9	6	8
US06INRD17	US3	Malus x domestica	Red Delicious	USA	11	9	3	2	2	7	?	5	2	10	4	9
US06INRD18	US3	Malus x domestica	Red Delicious	USA	?	13	21	3	14	7	6	8	11	7	6	10
US06INRD19	US3	Malus x domestica	Red Delicious	USA	11	9	12	3	14	7	8	7	5	9	6	2
US06INRD2	US3	Malus x domestica	Red Delicious	USA	11	9	11	2	18	7	?	8	8	10	4	2
US06INRD20	US3	Malus x domestica	Red Delicious	USA	?	9	11	2	2	7	10	4	5	3	6	2
US06INRD23	US3	Malus x domestica	Red Delicious	USA	12	9	11	2	4	7	8	5	8	3	6	9
US06INRD25	US3	Malus x domestica	Red Delicious	USA	12	10	11	3	14	7	10	15	8	3	6	9
US06INRD27	US3	Malus x domestica	Red Delicious	USA	11	9	3	2	2	7	10	6	11	3	4	10
US06INRD28	US3	Malus x domestica	Red Delicious	USA	12	9	11	2	13	7	9	8	8	3	4	10
US06INRD29	US3	Malus x domestica	Red Delicious	USA	11	3	?	2	?	7	8	8	8	3	4	8
US06INRD3	US3	Malus x domestica	Red Delicious	USA	11	9	11	2	?	7	8	5	8	5	6	7
US06INRD30	US3	Malus x domestica	Red Delicious	USA	11	9	3	3	14	7	9	4	8	3	6	7
US06INRD31.2	US3	Malus x domestica	Red Delicious	USA	?	9	3	3	15	7	7	5	8	10	6	8
US06INRD32.2	US3	Malus x domestica	Red Delicious	USA	12	9	?	2	14	7	8	5	8	10	6	2
US06INRD33	US3	Malus x domestica	Red Delicious	USA	12	10	16	2	2	7	8	7	8	10	6	7
US06INRD34.2	US3	Malus x domestica	Red Delicious	USA	?	9	?	2	17	7	10	8	8	10	6	2
US06INRD37	US3	Malus x domestica	Red Delicious	USA	11	10	11	2	14	7	10	7	5	3	6	8
US06INRD38	US3	Malus x domestica	Red Delicious	USA	11	10	11	2	12	7	8	4	5	10	6	8
US06INRD42	US3	Malus x domestica	Red Delicious	USA	11	10	?	2	17	9	8	5	11	3	6	7
US06INRD43	US3	Malus x domestica	Red Delicious	USA	11	9	2	2	16	7	8	6	8	6	6	2
US06INRD45	US3	Malus x domestica	Red Delicious	USA	11	9	2	2	14	6	8	5	5	18	6	9
US06INRD5	US3	Malus x domestica	Red Delicious	USA	11	9	11	2	?	7	16	4	5	10	6	2

US06INRD6	US3	Malus x domestica	Red Delicious	USA	11	9	11	2	14	7	11	5	5	3	6	10
US06INRD7	US3	Malus x domestica	Red Delicious	USA	3	9	?	3	6	7	10	4	8	10	6	2
US06INRD9.2	US3	Malus x domestica	Red Delicious	USA	11	8	13	2	17	7	7	5	2	9	6	9
US06KYGA1	US4	Malus x domestica	Royal Gala	USA	11	6	18	2	11	7	9	7	5	10	6	2
US06KYGA10	US4	Malus x domestica	Royal Gala	USA	3	9	19	2	14	9	10	8	8	14	3	2
US06KYGA11	US4	Malus x domestica	Royal Gala	USA	11	9	7	2	14	9	?	4	14	9	4	2
US06KYGA12	US4	Malus x domestica	Royal Gala	USA	3	9	11	2	7	9	9	15	5	6	6	10
US06KYGA13	US4	Malus x domestica	Royal Gala	USA	12	9	3	2	18	7	18	7	14	9	6	8
US06KYGA14	US4	Malus x domestica	Royal Gala	USA	11	9	17	2	14	7	?	10	5	6	4	8
US06KYGA16	US4	Malus x domestica	Royal Gala	USA	3	9	10	3	13	7	9	8	8	8	6	8
US06KYGA17	US4	Malus x domestica	Royal Gala	USA	12	9	17	2	16	6	?	4	5	15	6	2
US06KYGA18	US4	Malus x domestica	Royal Gala	USA	11	9	22	2	13	7	10	4	8	18	3	10
US06KYGA19	US4	Malus x domestica	Royal Gala	USA	11	9	10	2	17	7	?	10	5	6	6	8
US06KYGA2	US4	Malus x domestica	Royal Gala	USA	12	9	17	2	15	7	13	7	12	10	6	2
US06KYGA20	US4	Malus x domestica	Royal Gala	USA	11	9	28	2	14	7	?	8	5	10	6	2
US06KYGA21	US4	Malus x domestica	Royal Gala	USA	11	9	7	2	16	7	?	6	5	10	6	2
US06KYGA22	US4	Malus x domestica	Royal Gala	USA	13	9	?	2	6	7	?	6	8	10	6	8
US06KYGA23	US4	Malus x domestica	Royal Gala	USA	3	8	10	2	14	7	8	5	8	10	4	2
US06KYGA24	US4	Malus x domestica	Royal Gala	USA	3	9	10	2	14	7	7	6	5	10	7	2
US06KYGA25	US4	Malus x domestica	Royal Gala	USA	12	9	11	2	17	9	?	15	16	14	6	8
US06KYGA26	US4	Malus x domestica	Royal Gala	USA	3	9	?	2	6	7	10	7	5	12	6	2
US06KYGA28	US4	Malus x domestica	Royal Gala	USA	3	9	11	2	7	7	7	8	5	12	6	7
US06KYGA29	US4	Malus x domestica	Royal Gala	USA	12	9	?	2	18	9	?	7	5	11	6	8
US06KYGA3	US4	Malus x domestica	Royal Gala	USA	3	9	?	2	14	9	10	8	8	9	3	7
US06KYGA30	US4	Malus x domestica	Royal Gala	USA	11	8	10	2	16	7	?	10	5	8	6	2
US06KYGA31	US4	Malus x domestica	Royal Gala	USA	12	9	11	2	2	7	6	7	17	6	6	12
US06KYGA33	US4	Malus x domestica	Royal Gala	USA	11	9	11	2	14	7	?	16	5	10	4	2
US06KYGA34	US4	Malus x domestica	Royal Gala	USA	3	9	?	2	6	7	10	4	5	5	6	8
US06KYGA35	US4	Malus x domestica	Royal Gala	USA	3	9	2	2	7	7	?	15	5	14	6	7
US06KYGA38	US4	Malus x domestica	Royal Gala	USA	3	9	7	2	15	?	7	?	5	12	?	7
US06KYGA4	US4	Malus x domestica	Royal Gala	USA	11	9	?	3	14	7	10	6	5	11	6	7
US06KYGA40	US4	Malus x domestica	Royal Gala	USA	11	9	4	2	14	?	10	?	8	5	?	8
US06KYGA5	US4	Malus x domestica	Royal Gala	USA	11	9	19	2	?	7	9	7	5	10	6	7
US06KYGA7	US4	Malus x domestica	Royal Gala	USA	12	9	18	2	14	7	8	4	5	10	4	7
US06KYGA8	US4	Malus x domestica	Royal Gala	USA	11	9	6	2	18	9	7	7	15	9	6	8
US06KYGA9	US4	Malus x domestica	Royal Gala	USA	11	9	8	2	14	7	8	8	5	12	6	8
#153G1	CA	Malus x domestica	xxx	Canada	11	9	?	2	6	7	8	5	8	10	6	8
#169G1	CA	Malus x domestica	xxx	Canada	13	9	8	2	14	6	8	5	8	10	6	2
#183G1	CA	Malus x domestica	xxx	Canada	12	9	?	2	7	9	10	8	8	3	6	2
#224G1	CA	Malus x domestica	xxx	Canada	12	9	?	2	7	7	8	5	8	?	6	7
#225G1	CA	Malus x domestica	xxx	Canada	11	9	?	3	6	7	8	8	8	6	6	7

#225G1	CA	Malus x domestica	xxx	Canada	12	9	11	3	6	9	10	5	5	10	6	7
#236G2	CA	Malus x domestica	xxx	Canada	12	9	30	2	7	9	8	5	8	15	6	2
#258G2	CA	Malus x domestica	xxx	Canada	16	9	18	2	14	7	8	5	19	5	7	2
#380G1	CA	Malus x domestica	xxx	Canada	11	9	11	2	10	7	8	7	8	9	6	2
#427G2	CA	Malus x domestica	xxx	Canada	?	9	14	2	17	9	18	8	8	10	6	8
C1	CA	Malus x domestica	xxx	Canada	11	9	7	2	14	10	8	7	8	9	6	2
C3	CA	Malus x domestica	xxx	Canada	12	9	14	2	14	9	7	4	5	3	6	2
C4	CA	Malus x domestica	xxx	Canada	11	9	?	2	7	7	8	5	8	10	6	8
E1	CA	Malus x domestica	xxx	Canada	12	20	9	2	?	9	?	5	8	?	6	2
E2	CA	Malus x domestica	xxx	Canada	12	9	11	2	7	7	8	5	5	3	4	8
E3	CA	Malus x domestica	xxx	Canada	11	9	12	2	15	7	8	8	5	5	4	8
E4	CA	Malus x domestica	xxx	Canada	11	9	12	2	15	?	10	8	5	5	4	8
E5	CA	Malus x domestica	xxx	Canada	11	9	12	2	15	7	8	8	5	5	4	8
Gd1	CA	Malus x domestica	xxx	Canada	11	9	?	3	7	9	8	5	8	10	7	2
Gd2	CA	Malus x domestica	xxx	Canada	11	9	13	3	7	9	8	5	8	10	7	2
Gd3	CA	Malus x domestica	xxx	Canada	11	9	?	3	7	9	8	5	8	10	6	2
Gg1	CA	Malus x domestica	xxx	Canada	?	9	9	2	8	9	10	5	8	10	6	2
Gg2	CA	Malus x domestica	xxx	Canada	3	9	18	2	14	7	8	7	8	15	6	2
Gg3	CA	Malus x domestica	xxx	Canada	3	9	12	2	7	7	18	4	8	10	6	2
Gg4	CA	Malus x domestica	xxx	Canada	11	9	18	2	7	7	9	8	?	9	4	8
Gg5	CA	Malus x domestica	xxx	Canada	?	9	9	2	8	9	10	5	8	10	6	2
Gg6	CA	Malus x domestica	xxx	Canada	11	9	18	2	7	7	9	?	?	9	4	8
Gg7	CA	Malus x domestica	xxx	Canada	12	9	8	2	7	9	?	4	2	10	6	7
Pa2	CA	Malus x domestica	xxx	Canada	5	9	6	2	14	7	18	7	6	11	7	2
Pa3	CA	Malus x domestica	xxx	Canada	12	9	?	2	6	7	8	5	8	?	6	8
Pa4	CA	Malus x domestica	xxx	Canada	15	9	12	2	17	9	9	5	8	6	6	2
Pa5	CA	Malus x domestica	xxx	Canada	15	9	12	2	17	9	9	5	8	6	6	2
S1	CA	Malus x domestica	xxx	Canada	12	9	11	2	6	7	8	5	5	?	4	7
S2	CA	Malus x domestica	xxx	Canada	11	9	8	3	7	7	?	5	5	10	4	8
BR04GoldenF1L1	BR1	Malus x domestica	Golden Delicious	Brazil	12	9	15	2	7	6	9	8	8	3	6	9
BR04GoldenF10L1	BR1	Malus x domestica	Golden Delicious	Brazil	12	?	15	3	9	7	8	5	5	3	6	9
BR04GoldenF11L1	BR1	Malus x domestica	Golden Delicious	Brazil	12	9	15	2	?	7	9	8	8	3	6	1
BR04GoldenF12L1	BR1	Malus x domestica	Golden Delicious	Brazil	12	9	15	2	8	7	9	7	8	9	6	?
BR04GoldenF13L1	BR1	Malus x domestica	Golden Delicious	Brazil	11	9	15	2	9	7	9	7	5	3	6	?
BR04GoldenF14L1	BR1	Malus x domestica	Golden Delicious	Brazil	12	14	15	2	?	7	9	7	5	?	4	6
BR04GoldenF15L1	BR1	Malus x domestica	Golden Delicious	Brazil	12	9	17	2	?	7	9	4	8	?	6	8
BR04GoldenF16L1	BR1	Malus x domestica	Golden Delicious	Brazil	12	9	15	2	8	7	10	7	8	3	6	8
BR04GoldenF17L1	BR1	Malus x domestica	Golden Delicious	Brazil	12	9	15	2	8	7	10	7	8	8	6	8
BR04GoldenF18L1	BR1	Malus x domestica	Golden Delicious	Brazil	12	9	17	2	7	7	6	5	8	3	6	8
BR04GoldenF19L1	BR1	Malus x domestica	Golden Delicious	Brazil	12	?	16	3	8	7	9	7	8	13	6	10
BR04GoldenF2L1	BR1	Malus x domestica	Golden Delicious	Brazil	12	9	15	2	7	7	9	5	5	3	5	7

BR04GoldenF20L1	BR1	Malus x domestica	Golden Delicious	Brazil	12	9	15	2	7	7	9	7	5	3	6	9
BR04GoldenF21L1	BR1	Malus x domestica	Golden Delicious	Brazil	12	9	?	2	7	7	9	6	8	13	6	7
BR04GoldenF22L1	BR1	Malus x domestica	Golden Delicious	Brazil	12	9	15	2	?	7	9	7	8	?	6	7
BR04GoldenF23L1	BR1	Malus x domestica	Golden Delicious	Brazil	12	14	16	3	?	6	7	5	5	?	5	?
BR04GoldenF3L1	BR1	Malus x domestica	Golden Delicious	Brazil	12	10	15	2	7	9	9	7	5	3	5	8
BR04GoldenF4L1	BR1	Malus x domestica	Golden Delicious	Brazil	12	?	15	2	6	7	10	7	5	3	5	8
BR04GoldenF5L1	BR1	Malus x domestica	Golden Delicious	Brazil	12	9	15	2	7	7	9	5	8	10	6	7
BR04GoldenF6L1	BR1	Malus x domestica	Golden Delicious	Brazil	12	9	15	2	?	6	9	6	8	?	6	8
BR04GoldenF7L1	BR1	Malus x domestica	Golden Delicious	Brazil	12	?	17	2	?	6	9	5	8	?	6	7
BR04GoldenF8L1	BR1	Malus x domestica	Golden Delicious	Brazil	12	9	11	2	9	7	10	7	8	9	6	8
BR04GoldenF9L1	BR1	Malus x domestica	Golden Delicious	Brazil	12	9	16	2	7	9	11	5	8	3	6	8
BRE04Gold1c	BR1	Malus x domestica	Golden Delicious	Brazil	12	14	14	2	13	7	9	7	8	13	5	8
BRE04Gold2c	BR1	Malus x domestica	Golden Delicious	Brazil	12	14	14	3	7	7	9	7	5	13	4	8
BRE04Gold6b	BR1	Malus x domestica	Golden Delicious	Brazil	13	9	15	1	6	5	7	11	8	3	5	6
BRE04Gold8b	BR1	Malus x domestica	Golden Delicious	Brazil	3	14	18	2	7	7	14	5	8	12	6	8
BRE04Gold9b	BR1	Malus x domestica	Golden Delicious	Brazil	13	9	17	2	6	7	9	6	5	13	6	8
BR04BGCA11	BR2	Malus x domestica	Belgolden	Brazil	?	9	15	?	6	6	14	7	5	10	6	8
BR04BGCA12	BR2	Malus x domestica	Belgolden	Brazil	11	2	15	?	6	7	14	7	8	9	6	8
BR04BGCA13	BR2	Malus x domestica	Belgolden	Brazil	12	2	15	?	6	7	10	5	5	10	5	8
BR04BGCA14	BR2	Malus x domestica	Belgolden	Brazil	?	9	15	?	6	6	9	6	8	9	6	8
BR04BGCA15	BR2	Malus x domestica	Belgolden	Brazil	?	9	15	2	7	7	10	5	8	10	7	8
BR04BGCA16	BR2	Malus x domestica	Belgolden	Brazil	12	10	15	3	6	7	10	5	8	8	6	8
BR04BGCA17	BR2	Malus x domestica	Belgolden	Brazil	13	9	16	2	6	7	?	7	5	13	6	8
BR04BGCA18	BR2	Malus x domestica	Belgolden	Brazil	12	9	27	?	6	7	10	7	5	9	5	8
BR04BGCA19	BR2	Malus x domestica	Belgolden	Brazil	12	9	17	3	6	9	?	5	8	8	7	8
BR04BGCA2	BR2	Malus x domestica	Belgolden	Brazil	11	9	17	?	9	7	10	5	5	10	6	8
BR04BGCA20	BR2	Malus x domestica	Belgolden	Brazil	11	9	17	2	9	7	10	5	5	3	6	8
BR04BGCA21	BR2	Malus x domestica	Belgolden	Brazil	13	2	15	2	6	9	14	5	8	8	6	8
BR04BGCA22	BR2	Malus x domestica	Belgolden	Brazil	12	2	15	2	6	12	9	6	5	8	7	8
BR04BGCA23	BR2	Malus x domestica	Belgolden	Brazil	12	9	?	?	7	7	9	5	8	10	7	12
BR04BGCA24	BR2	Malus x domestica	Belgolden	Brazil	?	10	17	?	6	7	10	5	5	3	5	8
BR04BGCA25	BR2	Malus x domestica	Belgolden	Brazil	12	9	18	?	7	7	10	5	8	10	6	8
BR04BGCA26	BR2	Malus x domestica	Belgolden	Brazil	12	13	19	3	6	9	3	5	5	13	5	8
BR04BGCA27	BR2	Malus x domestica	Belgolden	Brazil	12	9	15	3	6	12	12	7	5	9	5	8
BR04BGCA28	BR2	Malus x domestica	Belgolden	Brazil	12	9	27	2	6	6	9	5	8	8	6	8
BR04BGCA29	BR2	Malus x domestica	Belgolden	Brazil	12	9	21	2	6	7	8	7	5	13	6	8
BR04BGCA3	BR2	Malus x domestica	Belgolden	Brazil	12	9	15	2	6	7	10	6	5	8	5	8
BR04BGCA30	BR2	Malus x domestica	Belgolden	Brazil	12	9	21	2	6	12	?	5	5	13	5	7
BR04BGCA31	BR2	Malus x domestica	Belgolden	Brazil	3	2	17	3	6	7	?	5	8	11	7	8
BR04BGCA32	BR2	Malus x domestica	Belgolden	Brazil	13	9	?	2	7	7	9	6	8	3	6	8
BR04BGCA33	BR2	Malus x domestica	Belgolden	Brazil	12	10	15	2	6	7	9	5	8	10	6	8

BR04BGCA34	BR2	Malus x domestica	Belgolden	Brazil	12	9	17	2	6	9	?	7	8	8	6	8
BR04BGCA35	BR2	Malus x domestica	Belgolden	Brazil	13	2	?	2	7	7	10	5	8	10	6	8
BR04BGCA36	BR2	Malus x domestica	Belgolden	Brazil	12	9	13	2	6	7	14	6	5	13	4	8
BR04BGCA37	BR2	Malus x domestica	Belgolden	Brazil	12	15	15	?	8	6	9	7	8	8	6	8
BR04BGCA38	BR2	Malus x domestica	Belgolden	Brazil	12	14	16	3	6	11	9	7	5	3	5	8
BR04BGCA39	BR2	Malus x domestica	Belgolden	Brazil	12	2	15	2	7	7	8	5	5	13	4	8
BR04BGCA4	BR2	Malus x domestica	Belgolden	Brazil	13	9	15	2	13	11	14	7	5	8	6	8
BR04BGCA40	BR2	Malus x domestica	Belgolden	Brazil	12	9	15	3	7	9	9	7	8	9	4	8
BR04BGCA41	BR2	Malus x domestica	Belgolden	Brazil	11	2	?	1	7	7	?	6	5	10	4	8
BR04BGCA5	BR2	Malus x domestica	Belgolden	Brazil	12	9	15	2	6	11	9	5	5	9	5	8
BR04BGCA6	BR2	Malus x domestica	Belgolden	Brazil	3	?	16	2	9	7	9	5	8	8	6	8
BR04BGCA7	BR2	Malus x domestica	Belgolden	Brazil	3	?	15	?	8	7	10	6	5	10	4	?
BR04BGCA8	BR2	Malus x domestica	Belgolden	Brazil	3	2	15	2	6	9	9	5	5	3	5	8
BR04BGCA9	BR2	Malus x domestica	Belgolden	Brazil	13	2	17	2	6	6	?	6	8	9	6	8
BR04GAV31	BR3	Malus x domestica	Royal Gala	Brazil	12	10	24	2	7	9	9	5	8	9	6	8
BR04GAV310	BR3	Malus x domestica	Royal Gala	Brazil	12	9	15	3	7	7	9	7	5	9	5	?
BR04GAV311	BR3	Malus x domestica	Royal Gala	Brazil	12	10	15	2	16	5	8	6	5	11	5	8
BR04GAV312	BR3	Malus x domestica	Royal Gala	Brazil	12	2	19	3	14	7	?	7	8	3	6	8
BR04GAV313	BR3	Malus x domestica	Royal Gala	Brazil	3	2	?	2	6	7	10	5	5	3	5	8
BR04GAV314	BR3	Malus x domestica	Royal Gala	Brazil	12	2	?	2	7	7	?	6	5	13	5	8
BR04GAV315	BR3	Malus x domestica	Royal Gala	Brazil	12	10	23	3	6	5	9	8	8	8	5	8
BR04GAV316	BR3	Malus x domestica	Royal Gala	Brazil	12	9	?	2	6	7	9	4	5	9	5	8
BR04GAV317	BR3	Malus x domestica	Royal Gala	Brazil	5	9	15	3	7	7	14	7	5	13	5	8
BR04GAV318	BR3	Malus x domestica	Royal Gala	Brazil	12	?	?	3	9	7	9	5	8	8	6	8
BR04GAV319	BR3	Malus x domestica	Royal Gala	Brazil	12	2	15	2	6	9	?	7	5	10	6	8
BR04GAV32	BR3	Malus x domestica	Royal Gala	Brazil	12	9	16	3	7	7	11	4	8	8	6	8
BR04GAV320	BR3	Malus x domestica	Royal Gala	Brazil	3	10	15	3	10	7	9	7	8	3	6	8
BR04GAV321	BR3	Malus x domestica	Royal Gala	Brazil	11	9	19	3	7	6	9	5	5	13	5	8
BR04GAV322	BR3	Malus x domestica	Royal Gala	Brazil	12	10	16	3	8	6	9	7	5	14	7	8
BR04GAV323.1	BR3	Malus x domestica	Royal Gala	Brazil	12	9	20	2	9	5	?	5	5	13	4	8
BR04GAV323.2	BR3	Malus x domestica	Royal Gala	Brazil	12	9	15	3	7	9	9	4	5	12	6	8
BR04GAV324	BR3	Malus x domestica	Royal Gala	Brazil	12	14	14	2	7	7	11	8	8	9	6	8
BR04GAV325.1	BR3	Malus x domestica	Royal Gala	Brazil	12	?	15	2	6	6	10	7	5	8	7	8
BR04GAV325.2	BR3	Malus x domestica	Royal Gala	Brazil	12	14	15	2	7	12	9	8	8	13	6	8
BR04GAV326	BR3	Malus x domestica	Royal Gala	Brazil	14	10	15	2	7	6	12	5	5	3	6	8
BR04GAV327.1	BR3	Malus x domestica	Royal Gala	Brazil	12	9	14	2	7	7	9	5	8	10	6	8
BR04GAV327.2	BR3	Malus x domestica	Royal Gala	Brazil	12	9	15	2	7	9	?	5	5	3	5	8
BR04GAV33	BR3	Malus x domestica	Royal Gala	Brazil	12	10	14	3	6	7	9	8	5	13	5	8
BR04GAV34	BR3	Malus x domestica	Royal Gala	Brazil	12	10	20	2	6	9	?	7	6	13	5	8
BR04GAV35	BR3	Malus x domestica	Royal Gala	Brazil	13	9	15	3	7	7	9	7	8	9	6	8
BR04GAV36	BR3	Malus x domestica	Royal Gala	Brazil	12	10	16	2	6	7	9	8	8	3	6	8

BR04GAV37	BR3	Malus x domestica	Royal Gala	Brazil	13	10	?	2	7	6	9	6	8	9	6	8
BR04GAV38	BR3	Malus x domestica	Royal Gala	Brazil	12	10	14	2	8	7	?	8	5	3	5	8
BR04GAV39	BR3	Malus x domestica	Royal Gala	Brazil	11	9	12	2	6	7	11	7	8	8	6	8
BR04FUjS3.1b	BR4	Malus x domestica	Fuji	Brazil	12	9	17	2	7	9	?	5	5	9	6	8
BR04FUjS3.2b	BR4	Malus x domestica	Fuji	Brazil	12	10	17	3	14	9	?	5	5	13	6	8
BR04FUjS3.3b	BR4	Malus x domestica	Fuji	Brazil	12	2	17	3	?	7	9	6	8	10	6	8
BR04FUjS3.4b	BR4	Malus x domestica	Fuji	Brazil	5	10	15	2	?	7	9	7	5	9	6	8
BR04FUjS3.5b	BR4	Malus x domestica	Fuji	Brazil	12	9	15	2	6	9	10	5	5	3	6	8
BR04FUjS3.6b	BR4	Malus x domestica	Fuji	Brazil	12	9	15	2	6	9	?	6	5	13	5	8
BR04FUjS3.7b	BR4	Malus x domestica	Fuji	Brazil	13	14	17	2	9	7	10	6	5	8	6	8
BR04FUjS3.8b	BR4	Malus x domestica	Fuji	Brazil	?	9	15	2	7	7	9	?	8	3	6	8
BRE04FUJS3.10	BR4	Malus x domestica	Fuji	Brazil	15	9	15	2	7	7	10	7	8	13	6	8
BRE04FUJS3.11	BR4	Malus x domestica	Fuji	Brazil	12	14	15	3	7	7	9	6	5	10	?	8
BRE04FUJS3.12	BR4	Malus x domestica	Fuji	Brazil	12	8	14	2	7	9	9	5	8	13	6	8
BRE04FUJS3.13	BR4	Malus x domestica	Fuji	Brazil	12	9	15	3	6	6	10	6	5	10	6	8
BRE04FUJS3.14	BR4	Malus x domestica	Fuji	Brazil	12	9	18	2	10	6	10	8	8	3	5	8
BRE04FUJS3.15	BR4	Malus x domestica	Fuji	Brazil	12	8	16	?	7	7	9	5	8	10	6	8
BRE04FUJS3.16	BR4	Malus x domestica	Fuji	Brazil	3	9	15	2	6	7	9	8	8	3	6	8
BRE04FUJS3.17	BR4	Malus x domestica	Fuji	Brazil	12	9	14	2	16	9	14	7	5	13	8	8
BRE04FUJS3.18	BR4	Malus x domestica	Fuji	Brazil	12	10	15	1	7	7	11	6	8	9	6	8
BRE04FUJS3.19	BR4	Malus x domestica	Fuji	Brazil	13	16	15	2	7	7	9	6	5	11	5	7
BRE04FUJS3.20	BR4	Malus x domestica	Fuji	Brazil	5	14	?	3	9	7	10	6	5	8	6	8
BRE04FUJS3.21	BR4	Malus x domestica	Fuji	Brazil	5	10	15	2	6	7	9	6	8	3	?	7
BRE04FUJS3.22	BR4	Malus x domestica	Fuji	Brazil	13	14	6	2	7	6	11	7	5	13	5	8
BRE04FUJS3.23	BR4	Malus x domestica	Fuji	Brazil	12	9	15	2	7	7	10	5	5	3	5	8
BRE04FUJS3.24	BR4	Malus x domestica	Fuji	Brazil	3	9	15	2	?	12	8	7	8	10	6	8
BRE04FUJS3.25	BR4	Malus x domestica	Fuji	Brazil	12	9	15	3	7	7	9	6	5	13	6	8
BRE04FUJS3.26	BR4	Malus x domestica	Fuji	Brazil	12	2	15	3	7	6	9	4	5	3	6	8
BRE04FUJS3.27	BR4	Malus x domestica	Fuji	Brazil	12	14	6	2	6	7	?	5	5	9	6	8
BRE04FUjS3.7	BR4	Malus x domestica	Fuji	Brazil	13	14	17	2	9	7	10	?	5	8	6	8
BRE04FUJS3.9	BR4	Malus x domestica	Fuji	Brazil	12	2	14	2	7	9	9	7	5	10	6	8
BR04GAV4.10	BR5	Malus x domestica	Royal Gala	Brazil	12	2	15	2	8	9	9	7	5	13	4	8
BR04GAV4.11	BR5	Malus x domestica	Royal Gala	Brazil	12	2	15	2	8	9	?	7	5	13	4	8
BR04GAV4.12	BR5	Malus x domestica	Royal Gala	Brazil	12	10	18	3	6	9	9	6	5	3	6	8
BR04GAV4.13	BR5	Malus x domestica	Royal Gala	Brazil	12	9	16	3	6	9	10	6	8	3	6	8
BR04GAV4.14	BR5	Malus x domestica	Royal Gala	Brazil	12	9	17	2	8	9	10	5	8	8	5	8
BR04GAV4.15	BR5	Malus x domestica	Royal Gala	Brazil	11	?	16	2	7	7	6	6	5	10	6	8
BR04GAV4.16	BR5	Malus x domestica	Royal Gala	Brazil	12	9	16	3	6	9	9	6	8	3	6	8
BR04GAV4.17	BR5	Malus x domestica	Royal Gala	Brazil	12	9	15	3	8	7	?	5	5	8	6	8
BR04GAV4.18	BR5	Malus x domestica	Royal Gala	Brazil	12	9	15	3	8	7	?	5	5	8	6	8
BR04GAV4.19	BR5	Malus x domestica	Royal Gala	Brazil	12	9	15	3	8	7	10	5	5	8	6	8

BR04GAV4.20	BR5	Malus x domestica	Royal Gala	Brazil	12	9	16	3	6	9	10	6	8	3	6	8
BR04GAV4.21	BR5	Malus x domestica	Royal Gala	Brazil	12	9	15	3	8	7	10	5	5	8	6	8
BR04GAV4.22	BR5	Malus x domestica	Royal Gala	Brazil	12	2	15	3	6	7	10	6	8	11	6	8
BR04GAV4.23	BR5	Malus x domestica	Royal Gala	Brazil	12	9	15	3	8	7	10	5	5	8	6	8
BR04GAV4.24	BR5	Malus x domestica	Royal Gala	Brazil	12	9	15	3	8	7	10	5	5	8	6	8
BR04GAV4.25	BR5	Malus x domestica	Royal Gala	Brazil	12	2	15	2	8	9	10	7	5	13	6	8
BR04GAV4.26	BR5	Malus x domestica	Royal Gala	Brazil	13	10	18	2	6	7	9	4	8	13	6	8
BR04GAV4.27	BR5	Malus x domestica	Royal Gala	Brazil	12	2	15	3	6	7	10	6	8	11	6	8
BR04GAV4.28	BR5	Malus x domestica	Royal Gala	Brazil	13	9	18	2	6	7	9	4	8	13	6	8
BR04GAV4.29	BR5	Malus x domestica	Royal Gala	Brazil	13	10	18	2	6	7	9	4	8	13	6	8
BR04GAV4.30	BR5	Malus x domestica	Royal Gala	Brazil	12	9	16	3	6	9	9	6	8	3	6	8
BR04GAV4.31	BR5	Malus x domestica	Royal Gala	Brazil	12	2	15	2	8	9	9	7	5	13	4	8
BR04GAV4.32	BR5	Malus x domestica	Royal Gala	Brazil	12	2	15	3	6	7	9	6	8	11	6	8
BR04GAV4.33	BR5	Malus x domestica	Royal Gala	Brazil	12	2	15	3	6	7	9	6	8	11	6	8
BR04GAV4.34	BR5	Malus x domestica	Royal Gala	Brazil	12	2	15	3	6	7	9	6	8	11	6	8
BR04GAV4.35	BR5	Malus x domestica	Royal Gala	Brazil	12	2	15	3	6	7	9	6	8	11	6	8
BR04GAV4.36	BR5	Malus x domestica	Royal Gala	Brazil	13	10	18	2	6	7	9	4	8	13	6	8
BR04GAV4.37	BR5	Malus x domestica	Royal Gala	Brazil	11	9	17	2	7	6	?	8	5	16	5	8
BR04GAV4.38	BR5	Malus x domestica	Royal Gala	Brazil	12	2	15	3	6	7	10	6	8	11	6	8
BR04GAV4.7	BR5	Malus x domestica	Royal Gala	Brazil	12	9	15	2	9	6	12	5	5	10	4	8
BR04GAV4.8	BR5	Malus x domestica	Royal Gala	Brazil	12	2	15	2	18	7	?	7	5	13	6	8
BR04GAV4.9	BR5	Malus x domestica	Royal Gala	Brazil	3	9	15	3	7	6	10	5	5	10	6	8
BR04GAV5.1	BR5	Malus x domestica	Royal Gala	Brazil	12	10	?	3	9	9	9	5	5	13	6	8
BR04GAV5.10	BR5	Malus x domestica	Royal Gala	Brazil	12	8	19	3	7	7	14	5	5	13	6	8
BR04GAV5.11	BR5	Malus x domestica	Royal Gala	Brazil	3	6	17	2	?	9	9	8	8	11	6	12
BR04GAV5.12	BR5	Malus x domestica	Royal Gala	Brazil	12	8	?	2	6	7	9	6	5	3	5	12
BR04GAV5.13	BR5	Malus x domestica	Royal Gala	Brazil	14	8	15	2	14	7	9	6	8	3	4	8
BR04GAV5.14	BR5	Malus x domestica	Royal Gala	Brazil	12	9	16	2	7	7	9	8	8	3	6	8
BR04GAV5.15	BR5	Malus x domestica	Royal Gala	Brazil	12	9	17	2	7	7	?	4	14	3	6	12
BR04GAV5.16	BR5	Malus x domestica	Royal Gala	Brazil	12	9	17	2	8	12	11	8	5	14	6	8
BR04GAV5.17	BR5	Malus x domestica	Royal Gala	Brazil	12	9	15	3	6	6	11	6	8	14	6	8
BR04GAV5.18	BR5	Malus x domestica	Royal Gala	Brazil	11	2	20	3	9	9	11	5	8	13	6	8
BR04GAV5.19	BR5	Malus x domestica	Royal Gala	Brazil	12	9	17	2	6	7	11	7	8	3	6	8
BR04GAV5.2	BR5	Malus x domestica	Royal Gala	Brazil	11	9	4	2	7	9	9	7	5	10	6	8
BR04GAV5.20	BR5	Malus x domestica	Royal Gala	Brazil	3	10	15	2	6	12	10	6	5	13	5	8
BR04GAV5.22	BR5	Malus x domestica	Royal Gala	Brazil	12	8	?	2	7	9	?	7	5	13	7	8
BR04GAV5.23	BR5	Malus x domestica	Royal Gala	Brazil	12	10	15	3	7	7	11	6	5	8	6	8
BR04GAV5.24	BR5	Malus x domestica	Royal Gala	Brazil	12	9	3	3	6	7	?	8	8	3	6	8
BR04GAV5.25	BR5	Malus x domestica	Royal Gala	Brazil	3	9	17	3	6	7	9	7	5	9	5	8
BR04GAV5.26	BR5	Malus x domestica	Royal Gala	Brazil	3	2	16	2	7	9	9	7	5	13	6	8
BR04GAV5.27	BR5	Malus x domestica	Royal Gala	Brazil	14	9	4	2	6	9	11	8	8	13	7	2

BR04GAV5.3	BR5	Malus x domestica	Royal Gala	Brazil	12	2	16	2	6	7	9	4	5	10	5	8
BR04GAV5.4	BR5	Malus x domestica	Royal Gala	Brazil	13	10	?	2	6	7	9	5	5	3	5	8
BR04GAV5.5	BR5	Malus x domestica	Royal Gala	Brazil	?	10	15	2	6	5	?	6	5	5	4	8
BR04GAV5.6	BR5	Malus x domestica	Royal Gala	Brazil	12	9	24	2	6	7	9	7	11	13	5	8
BR04GAV5.7	BR5	Malus x domestica	Royal Gala	Brazil	?	9	15	?	8	7	10	6	2	?	4	8
BR04GAV5.8	BR5	Malus x domestica	Royal Gala	Brazil	12	9	17	2	11	9	10	7	?	13	4	8
BR04GAV5.9	BR5	Malus x domestica	Royal Gala	Brazil	12	10	15	3	6	9	9	7	5	8	6	7
BR04GFV6.1	BR6	Malus x domestica	Royal Gala/Fuji	Brazil	12	9	16	2	?	7	9	5	5	3	6	8
BR04GFV6.10	BR6	Malus x domestica	Royal Gala/Fuji	Brazil	12	9	17	2	9	6	13	7	5	13	5	8
BR04GFV6.11	BR6	Malus x domestica	Royal Gala/Fuji	Brazil	11	10	16	2	7	7	9	7	5	13	5	8
BR04GFV6.12	BR6	Malus x domestica	Royal Gala/Fuji	Brazil	12	9	17	2	13	7	?	7	5	13	6	8
BR04GFV6.13	BR6	Malus x domestica	Royal Gala/Fuji	Brazil	13	10	16	2	6	7	14	5	8	10	6	8
BR04GFV6.14	BR6	Malus x domestica	Royal Gala/Fuji	Brazil	12	9	?	?	6	9	11	7	5	12	5	8
BR04GFV6.15	BR6	Malus x domestica	Royal Gala/Fuji	Brazil	?	10	17	?	6	7	10	5	5	10	5	8
BR04GFV6.16	BR6	Malus x domestica	Royal Gala/Fuji	Brazil	11	2	?	?	13	9	14	5	8	10	6	8
BR04GFV6.17	BR6	Malus x domestica	Royal Gala/Fuji	Brazil	11	2	15	?	7	7	?	?	8	10	6	8
BR04GFV6.18	BR6	Malus x domestica	Royal Gala/Fuji	Brazil	11	2	?	2	13	5	14	8	8	10	5	8
BR04GFV6.19	BR6	Malus x domestica	Royal Gala/Fuji	Brazil	12	9	15	?	8	5	?	6	2	10	4	?
BR04GFV6.2	BR6	Malus x domestica	Royal Gala/Fuji	Brazil	12	?	16	?	13	12	11	4	5	9	5	8
BR04GFV6.20	BR6	Malus x domestica	Royal Gala/Fuji	Brazil	12	10	16	2	7	7	11	6	5	9	5	8
BR04GFV6.21	BR6	Malus x domestica	Royal Gala/Fuji	Brazil	12	10	17	2	6	7	11	5	5	12	5	8
BR04GFV6.22	BR6	Malus x domestica	Royal Gala/Fuji	Brazil	12	2	17	2	7	7	10	6	5	10	5	8
BR04GFV6.23	BR6	Malus x domestica	Royal Gala/Fuji	Brazil	11	10	18	2	9	7	?	7	5	8	6	8
BR04GFV6.4	BR6	Malus x domestica	Royal Gala/Fuji	Brazil	13	2	17	2	6	5	10	6	5	10	4	8
BR04GFV6.5	BR6	Malus x domestica	Royal Gala/Fuji	Brazil	12	9	18	3	6	7	14	7	5	13	5	8
BR04GFV6.6	BR6	Malus x domestica	Royal Gala/Fuji	Brazil	12	10	15	2	6	7	9	9	5	13	6	8
BR04GFV6.7	BR6	Malus x domestica	Royal Gala/Fuji	Brazil	12	11	21	2	9	9	10	6	5	13	6	8
BR04GFV6.8	BR6	Malus x domestica	Royal Gala/Fuji	Brazil	11	9	15	2	6	5	?	6	5	9	5	8
BR04GFV6.9	BR6	Malus x domestica	Royal Gala/Fuji	Brazil	13	10	?	3	6	7	10	5	5	3	5	8
BRE04GALS8.10	BR7	Malus x domestica	Royal Gala	Brazil	12	10	15	2	6	7	10	7	5	13	5	8
BRE04GALS8.11	BR7	Malus x domestica	Royal Gala	Brazil	3	10	15	2	6	7	12	8	5	13	5	8
BRE04GALS8.12	BR7	Malus x domestica	Royal Gala	Brazil	12	14	15	2	7	7	9	6	8	3	6	8
BRE04GALS8.13	BR7	Malus x domestica	Royal Gala	Brazil	12	10	15	2	6	6	?	9	8	8	6	8
BRE04GALS8.14	BR7	Malus x domestica	Royal Gala	Brazil	12	9	15	2	6	9	9	6	8	13	6	8
BRE04GALS8.15	BR7	Malus x domestica	Royal Gala	Brazil	12	9	17	2	6	6	9	8	5	14	6	8
BRE04GALS8.16	BR7	Malus x domestica	Royal Gala	Brazil	12	9	15	2	6	7	9	6	5	12	5	?
BRE04GALS8.17	BR7	Malus x domestica	Royal Gala	Brazil	12	10	16	2	?	7	8	5	5	8	5	8
BRE04GALS8.18	BR7	Malus x domestica	Royal Gala	Brazil	12	10	15	2	6	6	9	5	8	13	5	8
BRE04GALS8.19	BR7	Malus x domestica	Royal Gala	Brazil	3	10	17	2	13	7	7	5	5	12	5	8
BRE04GAlS8.2	BR7	Malus x domestica	Royal Gala	Brazil	3	14	18	2	13	5	9	7	5	3	5	8
BRE04GALS8.20	BR7	Malus x domestica	Royal Gala	Brazil	12	14	15	2	?	7	9	6	8	3	6	8

BRE04GALS8.21	BR7	Malus x domestica	Royal Gala	Brazil	12	10	6	2	6	7	9	6	5	13	5	8
BRE04GALS8.22	BR7	Malus x domestica	Royal Gala	Brazil	3	10	17	2	7	12	9	5	8	12	6	8
BRE04GALS8.24	BR7	Malus x domestica	Royal Gala	Brazil	3	10	15	3	6	7	9	8	5	12	5	8
BRE04GALS8.25	BR7	Malus x domestica	Royal Gala	Brazil	13	9	6	2	6	12	10	8	8	8	5	8
BRE04GALS8.26	BR7	Malus x domestica	Royal Gala	Brazil	3	10	15	3	6	7	9	5	5	13	5	8
BRE04GAlS8.3	BR7	Malus x domestica	Royal Gala	Brazil	3	14	15	2	6	9	?	5	8	10	6	8
BRE04GAlS8.5	BR7	Malus x domestica	Royal Gala	Brazil	12	9	16	2	6	7	8	7	8	8	5	8
BRE04GAlS8.6	BR7	Malus x domestica	Royal Gala	Brazil	11	10	?	2	6	7	10	5	5	13	6	7
BRE04GAlS8.7	BR7	Malus x domestica	Royal Gala	Brazil	12	2	16	2	6	7	10	8	5	8	5	8
BRE04GAlS8.8	BR7	Malus x domestica	Royal Gala	Brazil	3	9	17	2	13	7	?	?	5	12	5	8
BRE04GALS8.9	BR7	Malus x domestica	Royal Gala	Brazil	12	10	16	2	6	7	7	5	5	8	5	8
SA05Gold1	SA	Malus x domestica	Golden Delicious	South Africa	13	9	8	5	5	7	12	8	18	6	4	8
SA05Gold11b	SA	Malus x domestica	Golden Delicious	South Africa	12	9	?	2	?	7	10	7	5	9	6	9
SA05Gold14b1	SA	Malus x domestica	Golden Delicious	South Africa	13	9	11	3	13	7	2	8	5	10	4	8
SA05Gold14b2	SA	Malus x domestica	Golden Delicious	South Africa	13	9	11	2	7	7	7	8	5	10	4	8
SA05Gold16b	SA	Malus x domestica	Golden Delicious	South Africa	12	2	8	2	15	7	?	8	5	10	4	8
SA05Gold3b	SA	Malus x domestica	Golden Delicious	South Africa	12	9	11	2	7	7	7	7	5	10	4	8
SA05Gold5b	SA	Malus x domestica	Golden Delicious	South Africa	13	9	11	3	13	7	10	6	5	6	4	8
SA05Gold6b1	SA	Malus x domestica	Golden Delicious	South Africa	13	10	8	3	7	7	?	8	5	10	4	8
SA05Gold7b1	SA	Malus x domestica	Golden Delicious	South Africa	3	9	12	3	13	7	15	8	5	16	4	8
SA05Gold7b2	SA	Malus x domestica	Golden Delicious	South Africa	13	9	8	2	?	7	?	6	5	9	4	8
SA05Gold2	SA	Malus x domestica	Golden Delicious	South Africa	3	10	7	3	13	?	7	?	?	10	4	?
SA05Gold8	SA	Malus x domestica	Golden Delicious	South Africa	14	10	17	3	7	5	8	?	13	16	5	2
NZ05GALF11	NZ1	Malus x domestica	Royal Gala	New Zealand	3	19	11	2	7	7	10	5	11	9	6	8
NZ05GALF12	NZ1	Malus x domestica	Royal Gala	New Zealand	3	9	4	3	7	7	9	5	11	8	6	7
NZ05GALF13	NZ1	Malus x domestica	Royal Gala	New Zealand	12	9	11	2	4	7	7	10	8	8	7	8
NZ05GALF14	NZ1	Malus x domestica	Royal Gala	New Zealand	3	9	11	2	6	7	10	6	2	9	6	8
NZ05GALF15	NZ1	Malus x domestica	Royal Gala	New Zealand	12	9	6	2	6	7	7	6	2	8	6	8
NZ05GALF16	NZ1	Malus x domestica	Royal Gala	New Zealand	12	9	6	2	7	6	8	6	8	9	6	8
NZ05GALF17	NZ1	Malus x domestica	Royal Gala	New Zealand	12	10	6	3	4	7	8	5	8	11	7	8
NZ05GALF18	NZ1	Malus x domestica	Royal Gala	New Zealand	12	19	6	2	6	7	8	6	8	8	6	8
NZ05GALF19	NZ1	Malus x domestica	Royal Gala	New Zealand	12	9	14	2	6	7	10	5	8	8	6	8
NZ05GALF20	NZ1	Malus x domestica	Royal Gala	New Zealand	11	9	15	2	?	7	11	5	8	9	6	8
NZ05GALF21	NZ1	Malus x domestica	Royal Gala	New Zealand	12	9	4	2	4	7	9	5	5	9	6	7
NZ05GALF22	NZ1	Malus x domestica	Royal Gala	New Zealand	12	19	6	2	6	7	8	6	8	8	6	8
NZ05GALF23	NZ1	Malus x domestica	Royal Gala	New Zealand	12	19	?	2	?	7	10	5	8	9	6	8
NZ05GALF24	NZ1	Malus x domestica	Royal Gala	New Zealand	12	19	?	2	6	7	8	6	8	8	6	8
NZ05GALF25	NZ1	Malus x domestica	Royal Gala	New Zealand	3	9	?	2	6	6	10	5	2	9	6	8
NZ05GALF26	NZ1	Malus x domestica	Royal Gala	New Zealand	3	9	6	2	6	6	10	5	2	9	6	8
NZ05GALF27	NZ1	Malus x domestica	Royal Gala	New Zealand	12	9	?	2	6	6	8	5	8	9	6	8
NZ05GALF28	NZ1	Malus x domestica	Royal Gala	New Zealand	12	9	?	2	4	7	7	5	8	8	6	8

NZ05GALF29	NZ1	Malus x domestica	Royal Gala	New Zealand	3	19	3	3	?	7	11	9	8	9	6	8
NZ05GALF30	NZ1	Malus x domestica	Royal Gala	New Zealand	12	10	12	3	?	7	8	5	8	11	7	8
NZ05GALF31	NZ1	Malus x domestica	Royal Gala	New Zealand	12	19	?	2	4	7	7	5	8	8	7	8
NZ05GALF32	NZ1	Malus x domestica	Royal Gala	New Zealand	12	9	6	2	6	7	7	5	8	8	6	8
NZ05GALF33	NZ1	Malus x domestica	Royal Gala	New Zealand	12	9	?	2	4	7	10	5	8	8	7	7
NZ05GALF34	NZ1	Malus x domestica	Royal Gala	New Zealand	12	10	6	2	6	7	7	6	8	9	7	8
NZ05GALF35	NZ1	Malus x domestica	Royal Gala	New Zealand	12	9	15	2	7	7	10	6	2	8	6	8
NZ05GALF36	NZ1	Malus x domestica	Royal Gala	New Zealand	12	9	17	2	6	7	8	6	8	8	6	8
NZ05GALF37	NZ1	Malus x domestica	Royal Gala	New Zealand	13	19	11	2	6	7	7	5	8	8	6	8
NZ06FU1	NZ2	Malus x domestica	Fuji	New Zealand	12	19	4	3	6	7	?	6	8	5	6	7
NZ06FU10	NZ2	Malus x domestica	Fuji	New Zealand	12	10	10	2	16	7	7	5	5	12	6	8
NZ06FU11	NZ2	Malus x domestica	Fuji	New Zealand	12	10	11	2	7	7	?	5	5	12	6	8
NZ06FU12	NZ2	Malus x domestica	Fuji	New Zealand	12	9	5	2	6	7	7	5	8	12	6	8
NZ06FU13	NZ2	Malus x domestica	Fuji	New Zealand	12	9	4	2	8	7	9	5	8	8	6	7
NZ06FU14	NZ2	Malus x domestica	Fuji	New Zealand	12	10	3	2	7	7	10	5	8	11	6	8
NZ06FU15	NZ2	Malus x domestica	Fuji	New Zealand	12	19	17	2	7	7	19	5	8	12	4	7
NZ06FU16	NZ2	Malus x domestica	Fuji	New Zealand	12	9	?	2	7	7	8	5	8	5	6	8
NZ06FU17	NZ2	Malus x domestica	Fuji	New Zealand	12	?	17	2	7	7	7	6	8	11	4	7
NZ06FU18	NZ2	Malus x domestica	Fuji	New Zealand	3	9	11	2	20	7	10	5	8	8	6	7
NZ06FU2	NZ2	Malus x domestica	Fuji	New Zealand	12	10	11	2	6	7	10	7	5	8	4	8
NZ06FU20	NZ2	Malus x domestica	Fuji	New Zealand	12	10	21	2	16	7	9	7	5	12	6	8
NZ06FU21	NZ2	Malus x domestica	Fuji	New Zealand	12	10	11	2	6	7	8	4	5	8	4	7
NZ06FU22	NZ2	Malus x domestica	Fuji	New Zealand	12	19	12	2	6	7	10	5	8	12	6	8
NZ06FU23	NZ2	Malus x domestica	Fuji	New Zealand	12	9	17	2	19	7	7	5	8	11	6	8
NZ06FU24	NZ2	Malus x domestica	Fuji	New Zealand	3	10	17	2	6	7	8	5	5	8	5	8
NZ06FU25	NZ2	Malus x domestica	Fuji	New Zealand	13	?	13	2	7	7	8	6	8	4	4	7
NZ06FU26	NZ2	Malus x domestica	Fuji	New Zealand	12	9	5	2	6	7	8	6	5	4	4	8
NZ06FU27	NZ2	Malus x domestica	Fuji	New Zealand	12	9	11	2	20	7	10	6	8	8	6	8
NZ06FU28	NZ2	Malus x domestica	Fuji	New Zealand	3	9	17	2	6	7	?	7	5	4	4	8
NZ06FU29	NZ2	Malus x domestica	Fuji	New Zealand	12	10	?	2	6	7	9	7	8	3	6	7
NZ06FU3	NZ2	Malus x domestica	Fuji	New Zealand	12	9	3	2	6	7	8	5	8	13	6	7
NZ06FU30	NZ2	Malus x domestica	Fuji	New Zealand	12	9	?	2	6	7	8	6	8	12	6	8
NZ06FU31	NZ2	Malus x domestica	Fuji	New Zealand	12	9	11	2	8	7	10	5	5	9	6	7
NZ06FU32	NZ2	Malus x domestica	Fuji	New Zealand	13	9	23	2	?	7	?	8	8	12	4	7
NZ06FU33	NZ2	Malus x domestica	Fuji	New Zealand	3	19	4	2	6	7	8	7	5	11	6	7
NZ06FU34	NZ2	Malus x domestica	Fuji	New Zealand	12	10	11	2	7	7	10	4	?	4	4	8
NZ06FU35	NZ2	Malus x domestica	Fuji	New Zealand	12	?	11	2	7	7	8	5	8	12	4	2
NZ06FU36	NZ2	Malus x domestica	Fuji	New Zealand	12	10	11	2	6	7	?	5	8	4	6	8
NZ06FU37	NZ2	Malus x domestica	Fuji	New Zealand	13	9	11	2	6	7	10	8	5	17	6	8
NZ06FU38	NZ2	Malus x domestica	Fuji	New Zealand	12	9	4	3	6	7	8	5	8	9	4	2
NZ06FU39	NZ2	Malus x domestica	Fuji	New Zealand	12	9	11	2	7	7	8	6	5	8	6	8

NZ06FU4	NZ2	Malus x domestica	Fuji	New Zealand	12	?	11	2	7	7	11	5	5	5	6	2
NZ06FU40	NZ2	Malus x domestica	Fuji	New Zealand	12	?	17	3	6	6	?	8	2	8	6	2
NZ06FU5	NZ2	Malus x domestica	Fuji	New Zealand	12	10	17	2	7	7	?	6	5	9	4	7
NZ06FU6	NZ2	Malus x domestica	Fuji	New Zealand	12	?	?	2	8	7	10	6	5	8	4	7
NZ06FU7	NZ2	Malus x domestica	Fuji	New Zealand	12	19	11	2	6	7	7	5	5	9	6	7
NZ06FU8	NZ2	Malus x domestica	Fuji	New Zealand	12	9	11	2	6	7	?	8	8	12	6	7
NZ06FU9	NZ2	Malus x domestica	Fuji	New Zealand	3	9	11	2	7	9	8	8	5	8	6	8
