# Supplementary material for: On the Origin and Spread of the Scab Disease of Apple: Out of Central Asia
Source: PLoS One. 2008 Jan 16;3(1):e1455. doi: 10.1371/journal.pone.0001455 (PMC2186383; doi:10.1371/journal.pone.0001455)
Supplement: Table S3 — Admixture analysis for 1180 haplotypes of Venturia inaequalis from seven regions: (A) average membership coefficients in seven clusters inferred with the individual-based method implemented in the Structure program [69], [70], and (B) average membership coefficients in six clusters inferred with the sample-based method implemented in Baps 4 [71]. (0.04 MB PDF) [file pone.0001455.s003.pdf]

Table S3. Admixture analysis for 1180 haplotypes of *Venturia inaequalis* from seven regions: (A) average membership coefficients in seven clusters inferred with the individual-based method implemented in the Structure program [69, 70], and (B) average membership coefficients in six clusters inferred with the sample-based method implemented in Baps 4 [71].

A

Clusters	Regional groups of samples						
	Central Asia	Europe	Morocco	North America	Brazil	New Zealand	South Africa
I	0.046	0.114	0.053	0.662	0.058	0.083	0.127
II	0.340	0.114	0.030	0.051	0.029	0.033	0.082
III	0.051	0.096	0.038	0.045	0.681	0.076	0.046
IV	0.339	0.120	0.038	0.052	0.035	0.032	0.252
V	0.102	0.341	0.051	0.059	0.066	0.069	0.347
VI	0.090	0.156	0.067	0.091	0.097	0.690	0.113
VII	0.033	0.058	0.722	0.041	0.036	0.018	0.032

B

Clusters	Regional groups of samples						
	Central Asia	Europe	Morocco	North America	Brazil	New Zealand	South Africa
I	0.041	0.069	0.045	0.730	0.027	0.026	0.143
II	0.703	0.110	0.019	0.037	0.013	0.010	0.172
III	0.062	0.117	0.030	0.039	0.823	0.051	0.029
IV	0.044	0.079	0.827	0.044	0.036	0.018	0.024
V	0.069	0.108	0.042	0.098	0.065	0.851	0.083
VI	0.081	0.517	0.037	0.052	0.035	0.045	0.548
