# Supplementary figures and images for: On the Origin and Spread of the Scab Disease of Apple: Out of Central Asia
Source: PLoS One. 2008 Jan 16;3(1):e1455. doi: 10.1371/journal.pone.0001455 (PMC2186383; doi:10.1371/journal.pone.0001455)

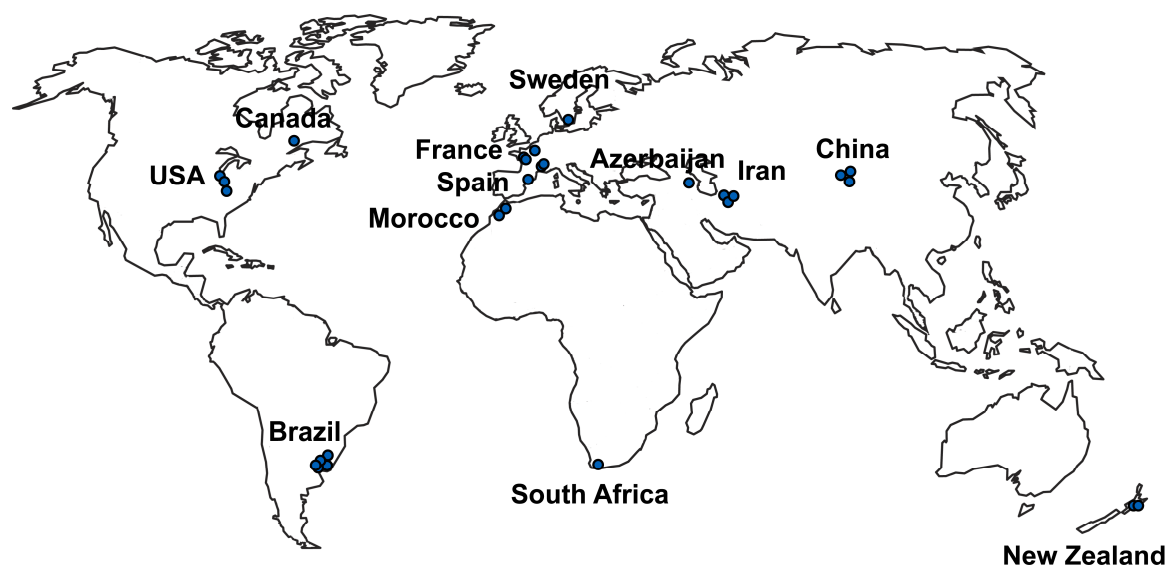

Figure S1. Map of approximate sampling locations.

Supplement: Figure S1 — Map of approximate sampling locations. (0.74 MB PDF) [file pone.0001455.s004.pdf]
