# Supplementary material for: On the Origin and Spread of the Scab Disease of Apple: Out of Central Asia
Source: PLoS One. 2008 Jan 16;3(1):e1455. doi: 10.1371/journal.pone.0001455 (PMC2186383; doi:10.1371/journal.pone.0001455)
Supplement: Figure S2 — Scatterplot expected heterozygosity, unique allele richness and arc surface distance from the most eastern sample. A least-square regression line represents the relationship between the two variables. Significance of the correlation was tested using Spearman's r (expected heterozygosity: r = −0.66, P<0.0001; unique allele richness: r = −0.58, P<0.0013). (0.14 MB PDF) [file pone.0001455.s005.pdf]

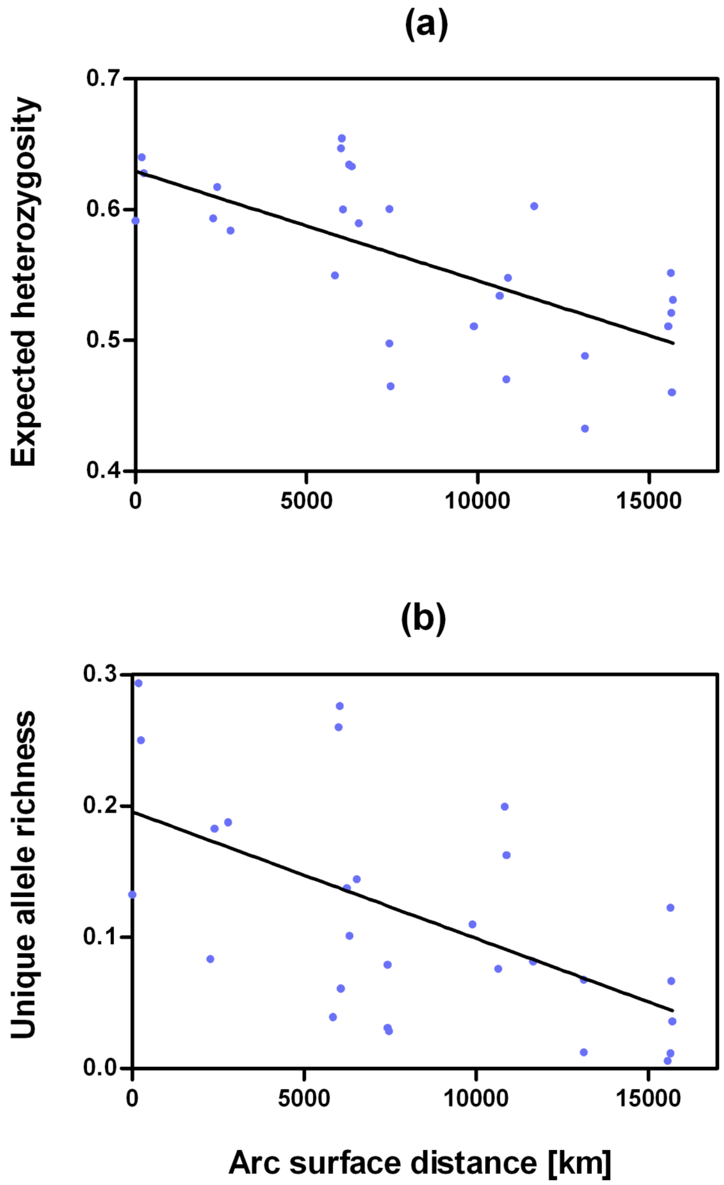

Figure S2. Scatterplot expected heterozygosity, unique allele richness and arc surface distance from the most eastern sample. A least-square regression line represents the relationship between the two variables. Significance of the correlation was tested using Spearman's r (expected heterozygosity: r=-0.66, P<0.0001; unique allele richness: r=-0.58, P<0.0013).
