# Supplementary material for: On the Origin and Spread of the Scab Disease of Apple: Out of Central Asia
Source: PLoS One. 2008 Jan 16;3(1):e1455. doi: 10.1371/journal.pone.0001455 (PMC2186383; doi:10.1371/journal.pone.0001455)
Supplement: Figure S3 — Plot of Ln likelihood of the data for several value of K, the parameter representing the number of populations in the Bayesian clustering algorithm implemented in the STRUCTURE program [69], [70]. Ln likelihood values were averaged across at least 6 independent runs of the program. (0.10 MB PDF) [file pone.0001455.s006.pdf]

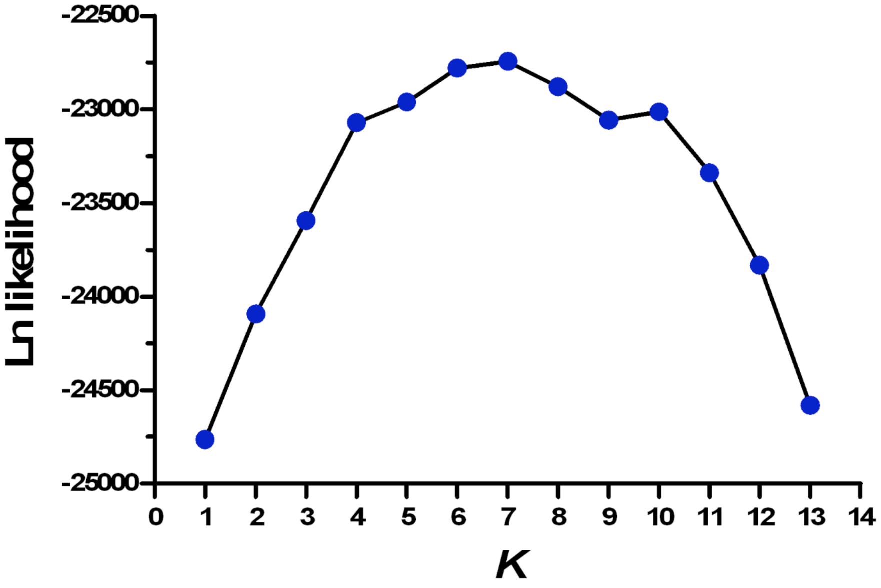

Figure S3: Plot of Ln likelihood of the data for several value of *K*, the parameter representing the number of populations in the Bayesian clustering algorithm implemented in the STRUCTURE program [69, 70]. Ln likelihood values were averaged across at least 6 independent runs of the program.
