# Supplementary material for: On the Origin and Spread of the Scab Disease of Apple: Out of Central Asia
Source: PLoS One. 2008 Jan 16;3(1):e1455. doi: 10.1371/journal.pone.0001455 (PMC2186383; doi:10.1371/journal.pone.0001455)
Supplement: Text S1 — Abstract in Chinese, French, Arabic and Portuguese (0.04 MB DOC) [file pone.0001455.s007.doc]

**Abstract in Chinese**

摘要

研究背景

苹果黑星病菌（*Venturia inaequalis*）属子囊菌亚门真菌，该病害在世界范围内严重危害苹果生长发育、产量和品质。探明苹果黑星病病原菌的起源、传播途径及群体生物学特性，利于对其进行有效的预测和检控。基于对该类病菌群体遗传框架正确推理的基础上，分析其遗传多样性，利于建立该病原菌的防控制技术信息和资料。

方法和发现

本研究对来自5个不同陆地（7个地区）28个果园的1273个黑星病菌株进行了群体微卫星遗传学分析，结果发现不同地区的受试菌株变异率达88%，该结果与过去黑星病菌在同一地区内或不同地区间的迁移习性趋于一致。此外，病菌群体结构分析发现，受试黑星病菌聚类组的划分与病菌地理来源基本一致，而且来自不同地区的不同寄主上的菌株被划分为不同的聚类组。综合比较受试病菌群体遗传变异水平及不同地区间该病菌的传播特性和聚类组遗传距离的差异，初步假定该病原菌起源于亚洲中部地区，后来随着苹果大量种植和市场贸易而逐渐传入欧洲及其它地区。在该病菌传入后，随着病菌地区适应性变异水平的增加和与其寄主品种互作而引起的变异，使该病菌的群体结构明显增加。结果表明，绝大多数黑星病菌群体具丰富的遗传多样性和随即的等位基因互换潜能，而且其繁殖特性并未因其异地传入而发生根本的变化。

结论和意义

苹果黑星病菌是一种模式病原真菌，目前在不同地区范围内病害发生危害程度十分严重，并具有丰富的群体遗传多样性，并且该类病菌主要以有性生殖方式进行繁殖和蔓延。

**Abstract in French**

*Contexte*

*Venturia inaequalis* est un champignon ascomycete responsable de la tavelure du pommier, une maladie qui a envahi presque toutes les régions du monde où le pommier est cultivé posant ainsi de graves problèmes en production. Prévenir et enrayer efficacement la réussite d’un tel succès invasif nécessite des connaissances approfondies sur l’origine, les voies d’introduction, la biologie et la génétique de ces populations invasives. En utilisant le potentiel d’inférence de la génétique des populations, l’analyse de la variation de marqueurs génétiques offre la possibilité d’accéder à ces informations.

*Méthodologie et Principaux résultats*

Ici nous présentons l’analyse de données microsatellites obtenues pour 1273 souches de *V. inaequalis* provenant de 28 vergers prélevées dans 7 régions sur les 5 continents. L’analyse de la variance moléculaire révèle que 88% de la variation se retrouve dans les vergers échantillonnés, ce qui est compatible avec d’importantes migrations historiques du champignon entre et à l’intérieur même des régions. Malgré cette très faible structuration des populations, les différentes analyses de clustering mettent en évidence un partage des populations en groupes séparés correspondant à leur origine géographique, montrant ainsi que chaque région héberge une population distincte du champignon. Ensemble, les résultats obtenus sur la comparaison du niveau de variabilité entre populations, les analyses de coalescence et les modèles de migration testés plaident en faveur d’un scénario dans lequel le champignon aurait émergé d’Asie Centrale, où le pommier a été domestiqué, avant d’être introduit en Europe puis plus récemment dans les autres continents suite à l’expansion de la culture du pommier. Les niveaux de variabilité indiquent que ces territoires ont subi des introductions multiples et que les populations portent toutes des signatures révélant de fortes expansions démographiques après leur introduction. Enfin, la forte diversité génotypique des populations et l’association aléatoire des allèles entre loci suggèrent que le champignon présente une reproduction sexuée régulière à la fois dans les régions où il a été introduit et dans sa région native.

*Conclusion et Portée.*

*Venturia inaequalis* est un modèle de champignons phytopathogène invasif qui a maintenant atteint le stade ultime du processus invasif, c’est à dire une très large distribution géographique par des populations bien établies montrant une grande diversité génétique, une reproduction sexuée régulière et une histoire d’expansion démographique.

**Abstract in Arabic**

السياق العام

يعتبر الجرب الأسود على الدراق من الفطريات الجعيبية المسؤولة عن تبقع شجرة التفاح، و هو مرض اجتاح جميع مناطق العالم التي يزرع فيها التفاح مما الحق أضرارا كبيرة بالإنتاج.

إن الوقاية من هذا المرض و الحد من انتشاره عبر تبني استراتيجيات تدخل ناجعة يتطلب معرفة جيدة بأصل المرض و طرق تنقله و انتشاره بالإضافة إلى معرفة بيولوجيا ووراثة الجمهرات المجتاحة وهي معلومات يمكن التوصل إليها بنهج طرق الاستدلال المستعملة في وراثة الجمهرات،و تحليل الواسمات الجينية .

المنهجية و النتائج

نقدم من خلال هذه الدراسة التحليل الجيني بواسطة المجهر الدقيق لجمهرة متكونة من 1273 أصل من الجرب الأسود على الدراق تنتمي إلى 28 بستانا مأخوذة من سبع مناطق تمثل خمس قارات.

يكشف تحليل شروط التغيير الذري أن 88 % من شروط التغيير توجد بالبساتين التي خضعت للمعايرة و هو ما يتوافق ( ينسجم ) مع التنقل التاريخي الهام لهذا الفطر داخل الجهات و فيما بينها.

و بالرغم من أن هذه الجمهرات تتوفر على بنيات ضعيفة فان مجموعة النتائج التي تم تحصيلها عبر مقارنة مستوى التغيير و التحاليل" العنقودية" توضح توزيع هذه المجموعات إلى أقسام منفصلة و ذلك بحسب أصلها الجغرافي، مما يدل على أن كل منطقة تأوي مجموعة مختلفة من هذا الفطر.

إن مجمل النتائج المحصل عليها من خلال مقارنة مستوى تغير الجمهرات و تحاليل أ الاندماج و نماذج التنقل التي تم اختبارها تدل على أن هذا النوع من الفطر ظهر في آسيا الوسطى

حيث نشأت زراعة التفاح وانتقلت بعد ذلك إلى أروبا ثم حديثا إلى باقي القارات حيث اتشرت هذه الزراعة. تدل مستويات التغير على أن المجموعات التي تم إدخالها خضعت لعمليات تطعيم كبيرة . و أخيرا فإن التنوع الوراثي الهام لهذه المجموعات و الجمع العشوائي للمورثات خارج مهد أصلها لم يؤثر على نظامها التكاثري.

استنتاج

يعتبر الجرب الأسود على الدراق نوعا من أمراض النباتات المنتشرة التي بلغت الآن المرحلة النهائية من صيرورة الاجتياح ( الانتشار)مع توزيع جغرافي بمجموعات محددة تشكل تنوعا وراثيا هاما ، و تكاثرا جنسيا و توسعا ديمغرافيا كذلك.

الجرب الأسود على الدراق ( FaoTerms) venturia inaequalis

**Abstract in Portuguese (the authors thank Paulo Ricardo de Oliveira for translation work)**

*Antecedentes*

*Venturia inaequalis* é um fungo ascomiceto agente causal da sarna da macieira, uma doença presente em quase todas as regiões produtoras de maçã do mundo, com os correspondentes efeitos adversos na produção dessa fruta. Monitorar e predizer a efetividade das estratégias de intervenção requer conhecimento da origem, das vias de introdução e da biologia das populações do patógeno. Análises da variação de marcadores genéticos, usando a base inferencial da genética de populações, oferecem potencial para acessar essas informações.

# Metodologia/Resultados Principais

É apresentada uma análise genética da população por variação de microsatélites em 1273 raças de *V. inaequalis*, representando 28 pomares, amostrados em 7 regiões de 5 continentes. A análise da variância molecular revelou que a maior parte da variação (88%) foi distribuída dentro de localidades, o que é consistente com extensivas migrações históricas de fungos entre e dentro de regiões. Apesar dessa estrutura populacional horizontalizada, a análise de agrupamento dividiu os dados em grupos que corresponderam grosseiramente à geografia, indicando que cada região abriga uma população distinta de fungos. A comparação dos níveis de variabilidade entre populações, juntamente com análise da coalescente de modelos de migração e estimativas das distâncias genéticas, foram consistentes com o cenário no qual o fungo surgiu na Ásia Central, onde a macieira foi domesticada, antes de ser introduzida na Europa e, mais recentemente, em outros continentes com a expansão do cultivo da maçã. Considerando a amplitude original, os níveis de variabilidade indicam múltiplas introduções e todas as populações mostram evidências de crescimentos pós-introdução em tamanho de população significativos. A maioria das populações exibiram alta diversidade genotípica e associação aleatória dos alelos, indicando que a migração dos fungos de seu local abrangência original não alterou o seu modo de reprodução.

# Conclusões/Importância

*Venturia inaequalis* é um modelo de fungo fitopatogênico invasivo que agora alcançou o estágio mais avançado do processo de invasão, com uma ampla distribuição geográfica e populações bem estabelecidas, exibindo alta variabilidade genética, reprodução sexual regular e expansão demográfica.
